# BODIPY-Based Fluorescent Probes for Sensing Protein Surface-Hydrophobicity

**DOI:** 10.1038/srep18337

**Published:** 2015-12-18

**Authors:** Nethaniah Dorh, Shilei Zhu, Kamal B. Dhungana, Ranjit Pati, Fen-Tair Luo, Haiying Liu, Ashutosh Tiwari

**Affiliations:** 1 Department of Chemistry, Michigan Technological University, Houghton, MI 49931, USA; 2 Department of Physics, Michigan Technological University, Houghton, MI 49931, USA; 3 Institute of Chemistry, Academia Sinica, Taipei, Taiwan 11529, Republic of China

## Abstract

Mapping surface hydrophobic interactions in proteins is key to understanding molecular recognition, biological functions, and is central to many protein misfolding diseases. Herein, we report synthesis and application of new BODIPY-based hydrophobic sensors (HPsensors) that are stable and highly fluorescent for pH values ranging from 7.0 to 9.0. Surface hydrophobic measurements of proteins (BSA, apomyoglobin, and myoglobin) by these HPsensors display much stronger signal compared to 8-anilino-1-naphthalene sulfonic acid (ANS), a commonly used hydrophobic probe; HPsensors show a 10- to 60-fold increase in signal strength for the BSA protein with affinity in the nanomolar range. This suggests that these HPsensors can be used as a sensitive indicator of protein surface hydrophobicity. A first principle approach is used to identify the molecular level mechanism for the substantial increase in the fluorescence signal strength. Our results show that conformational change and increased molecular rigidity of the dye due to its hydrophobic interaction with protein lead to fluorescence enhancement.

Protein folding and stability in aqueous solution is governed by a delicate balance of hydrogen bonding, electrostatic interaction, and hydrophobic interactions; hydrophobic interactions provide the major structural stability to the proteins^1^,^2^,^3^. Surface hydrophobic interactions are fundamental to protein-ligand interaction, molecular recognition^4^, and may influence intermolecular interactions and biological functions^5^,^6^. Furthermore, point mutations and (or) oxidative damage of proteins can result in increased surface hydrophobicity of proteins and have been linked to several age-related proteinopathies^7^,^8^,^9^,^10^,^11^,^12^. As a result, there has been a growing interest and need for developing probes and methods for sensing protein surface hydrophobicity^13^,^14^,^15^,^16^,^17^ as this can help to design better drug molecules based on surface properties^18^,^19^,^20^,^21^.

Many extrinsic fluorophores have been designed and used to study protein dynamics including protein folding and misfolding processes that have led to a better understanding of several proteinopathies including neurodegenerative diseases. However, only a few fluorophores that can measure protein surface hydrophobicity have been reported thus far: this includes dyes such as 8-anilino-1-naphthalene sulfonic acid (ANS), 4,4′-dianilino-1,1′-binaphthyl-5,5′-disulfonic acid (Bis-ANS), 6-propionyl-2-(N,N-dimethylamino)naphthalene (PRODAN), tetraphenylethene derivative, and Nile Red^5^,^15^,^16^,^22^,^23^. For characterization of most of these dyes, bovine serum albumin (BSA) and human serum albumin (HSA) have been used as test proteins. Of all these dyes, ANS is the most commonly used dye for measuring surface hydrophobicity. However, ANS dye is fraught with many issues such as: 1) it is an anionic dye and can contribute to fluorescence by both electrostatic as well as hydrophobic interactions leading to overestimation of fluorescence signal, and 2) it does not give measurable fluorescence signal when bound to solvent exposed hydrophobic surface of proteins due to quenching^5^,^15^,^24^,^25^,^26^. The other dye PRODAN, is a solvent-sensitive, neutral, fluorescent probe that has comparable fluorescence signal to ANS near physiological pH but has very poor solubility in water^5^,^15^. To address these problems, we have synthesized a series of 4,4-difluoro-4-bora-3a,4a-diaza-s-indacene (BODIPY) based hydrophobic sensors (HPsensors) for measuring protein hydrophobicity and tested these sensors on three proteins: BSA, myoglobin (Mb), and apomyoglobin (ApoMb). We chose BODIPY dyes for several reasons: they are highly fluorescent in non-polar media but are also fluorescent in polar (aqueous) media, have sharp and narrow emission peaks, and possess reduced solvatochromic shifts^27^,^28^. In addition, BODIPY dyes are highly tunable^29^,^30^,^31^,^32^ making them excellent candidates for the purpose of selectively reporting the hydrophobicity of proteins.

In this article we have focused our efforts on aryl substitution at 8-position (*meso*) on BODIPY dye for hydrophobic sensing of proteins. In Fig. 1, we show the structures of the synthesized HPsensors along with the **control** dye^27^,^33^,^34^ arranged in order of increasing electron donating ability. We substituted 2-methoxyethylamine group at 3,5-positions of the BODIPY core that increases water solubility. These HPsensors show weak but measurable fluorescence signal in water but are highly fluorescent in non-polar environment (Supplementary Figure 1). Furthermore, these HPsensors when tested with proteins (myoglobin (Mb), apomyoglobin (ApoMb), and BSA) show high fluorescence signal for hydrophobic proteins, BSA and ApoMb. Under same experimental conditions HPsensor **2** shows a 60-fold increase in fluorescence signal strength for BSA compared to that observed for ANS with affinity in the nanomolar range, making this dye a very sensitive indicator of protein surface-hydrophobicity (*S*_*0*_).

## Results

### Synthesis and characterization of fluorescent probes

**Figure 1:**
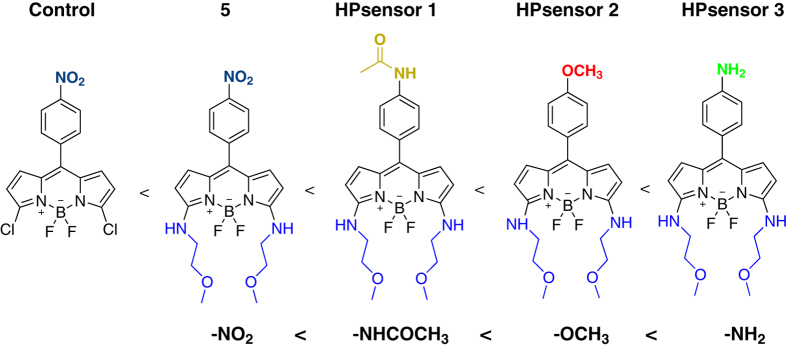
BODIPY dye structures in order of increasing electron donating ability. Schematic of dyes (**control**, dye **5**, and HPsensors **1**, **2**, and **3**) shown here were synthesized according to detailed protocol outlined in the supplementary methods.

**Figure 2:**
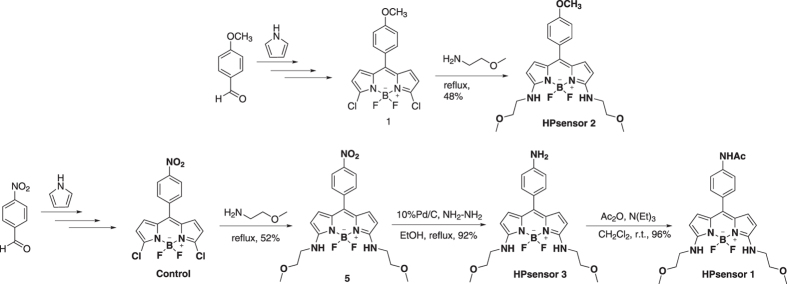
Synthetic route to HPsensors (1, 2, and 3), control dye, and dye 5. Probe **1** and **control** were prepared by using 4-methoxybenzaldehyde and 4-nitrobenzaldehyde. Dye **5** and HPsensor **2** were prepared by replacing the chlorine groups at 3,5-positions of dyes **1** and **control** with a nucleophile, 2-methoxyethan-1-amine. HPsensor **3** was prepared by reducing a nitro group at meso-position of BODIPY dye **5** via catalytic hydrogenation using palladium-on-carbon in the presence of hydrazine. HPsensor **1** was prepared by reacting an amino group at meso-position of HPsensor **3** with acetic anhydride at room temperature to form an amide bond.

The synthesis of dyes (Figs 1 and 2; see supplementary methods for details) was done by aryl substitutions at the *meso* position of the BODIPY core that increases dye sensitivity to solvent polarity and protein hydrophobicity; and substitution of chloro groups with 2-methoxyethylamine groups at the 3,5-positions enhances water solubility (Fig. 2). All dyes synthesized were fluorescent except for dye **5** (Supplementary Fig. 1). We calculated the quantum yield of each dye in three different solvents water, ethanol, and dichloromethane (Supplementary Table 1; Supplementary Figs 2 to 5). Quantum yield data on the HPsensors showed the greatest yield in ethanol and dichloromethane with the yield in water being the lowest which was similar to that of the **control** dye. We then determined the extinction coefficient of HPsensors **1**, **2**, **3**, and **control** dye in ethanol. The measurements indicated an extinction coefficient of 14880 μM^−1^ cm^−1^ for **control** dye. In contrast, for the HPsensors **1**, **2**, and **3** extinction coefficients were 50990, 31930 and 53920 μM^−1^ cm^−1^, respectively (Supplementary Table 1). The dyes were tested for the effect of pH on fluorescence intensity using Carmody buffer series in pH range from 2 to 12 (Supplementary Figs 6 and 7). The HPsensors (**1**, **2**, and **3**) are highly fluorescent for pH values ranging from 7.0 to 9.0 with maximum fluorescence observed in 60% ethanol (ethanol-water mixture) (Supplementary Figs 1 and 6). In addition, the dyes showed negligible response to ions (Na^+^, Mg^2+^, Fe^2+^, Fe^3+^, Ca^2+^, Zn^2+^) commonly found in buffer solutions (Supplementary Fig. 8).

### Response of dyes to protein hydrophobicity

**Figure 3:**
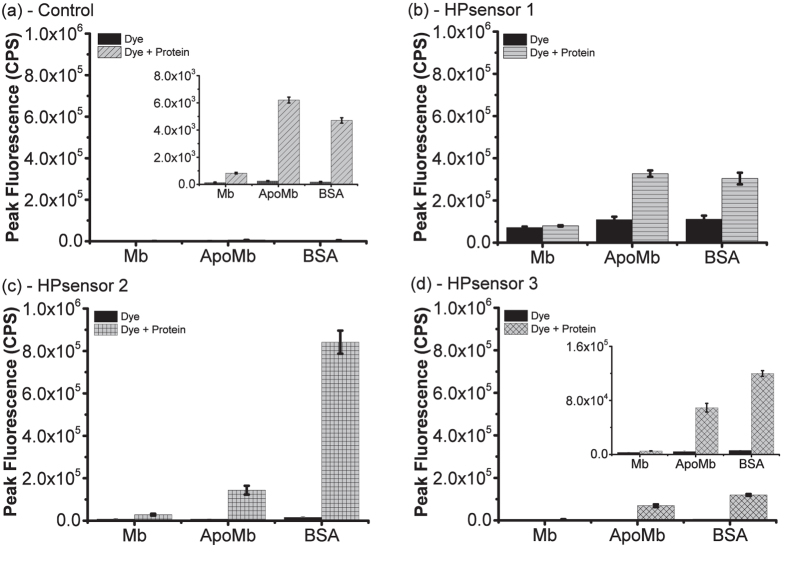
Mean peak fluorescence intensity of **control** (**a**), HPsensors 1 (**b**), 2(**c**), and 3(**d**) with Mb, ApoMb, and BSA proteins compared to free dye in water. All bar graphs were plotted on the same scale for ease of comparison. For **control** dye and HPsensor **3**, an inset bar graph with a smaller scale is also shown. All experiments were done in triplicate. Error bars indicate ± SD. Peak mean fluorescence used for plotting bar graphs are as follows: control dye at 540 nm, HPsensor **1** at 584 nm, HPsensor **2** at 579 nm, and HPsensor **3** at 578 nm.

**Figure 4:**
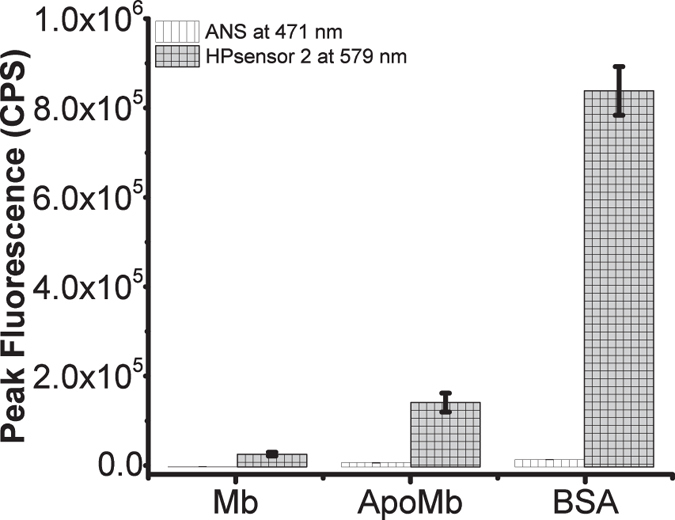
Mean peak fluorescence intensity of ANS and HPsensor 2 with Mb, ApoMb, and BSA proteins shown at the indicated wavelengths. All experiments were done in triplicate. Error bars indicate ± SD. Excitation wavelength used for ANS is 350 nm and for HPsensor **2** is 528 nm.

**Figure 5:**
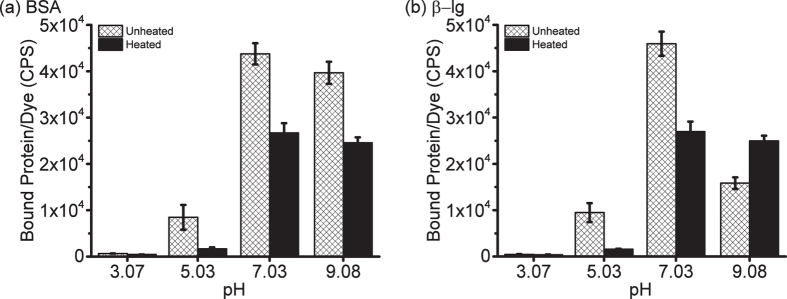
HPsensor 2 sensitivity to change in proteins surface polarity. 0.1** **μM of proteins (**a**) BSA and (**b**) beta lactoglobulin (β-lg) were incubated with 0.5 μM of HPsensor **2** at 25 °C in Carmody Buffer at pH 3, 5, 7 or 9. All experiments were done in triplicate and average peak fluorescence at 579 nm was used to calculate bound protein/dye. Error bars indicate ± SD. Excitation wavelength used for HPsensor **2** was 528 nm.

We first tested the dyes with BSA to determine the appropriate concentration to be used for protein studies. The dyes show a linear fluorescence response for 2 μM of BSA at low dye concentration i.e. 1:1 or 1:2 protein:dye ratio (Supplementary Fig. 9). Therefore, for measuring the relative protein hydrophobicity, HPsensors were tested with BSA, ApoMb, and Mb at 1:1 ratio of dye to protein (2 μM each) (Fig. 3, Supplementary Figs 10–12). In the presence of proteins, dyes exhibited a strong fluorescence signal for ApoMb and BSA but a weak signal for Mb (Fig. 3, Supplementary Figs 10–12). All three HPsensors showed a progressive 3- to 11-fold increase in fluorescence signal for ApoMb and a 3- to 33-fold increase for BSA when compared to Mb for the respective dyes (Fig. 3); HPsensor **2** showed the greatest fluorescence increase for BSA. The signal for HPsensor **1** was nearly half of the signal observed for HPsensors **2** (Fig. 3). In comparison, the **control** dye showed very weak fluorescence signal for proteins (Fig. 3). When compared to ANS, a well-known hydrophobic dye for proteins under similar conditions, HPsensor **2** gave 10- to 60-fold higher signal for the test proteins Mb, ApoMb, and BSA (Fig. 4, Supplementary Fig. 13). Therefore, we measured the dissociation constant (*K*_*d*_) for HPsensor **2** for the three proteins and determined it to be 1.2 μM for Mb, 0.33 μM for ApoMb, and 0.034 μM for BSA (Supplementary Fig. 16). In addition, we measured the surface hydrophobicity (*S0*) of proteins in presence of HPsensor **2** and determined it to be 2934 for Mb, 65212 for ApoMb, and 658608 for BSA (Supplementary Fig. 17). To ascertain if change in surface polarity of proteins also affects HPsensor binding, we tested HPsensor **2** with two well studied proteins BSA and beta-lactoglobulin (β-lg) (Fig. 5). HPsensor **2** shows reduced fluorescence signal at low pH (Fig. 5). In comparison, heated proteins (both BSA and β-lg) showed even lower fluorescence signal than respective unheated proteins (Fig. 5). The only exception was pH 9 fluorescence signal for β-lg (Fig. 5).

**Figure 6:**
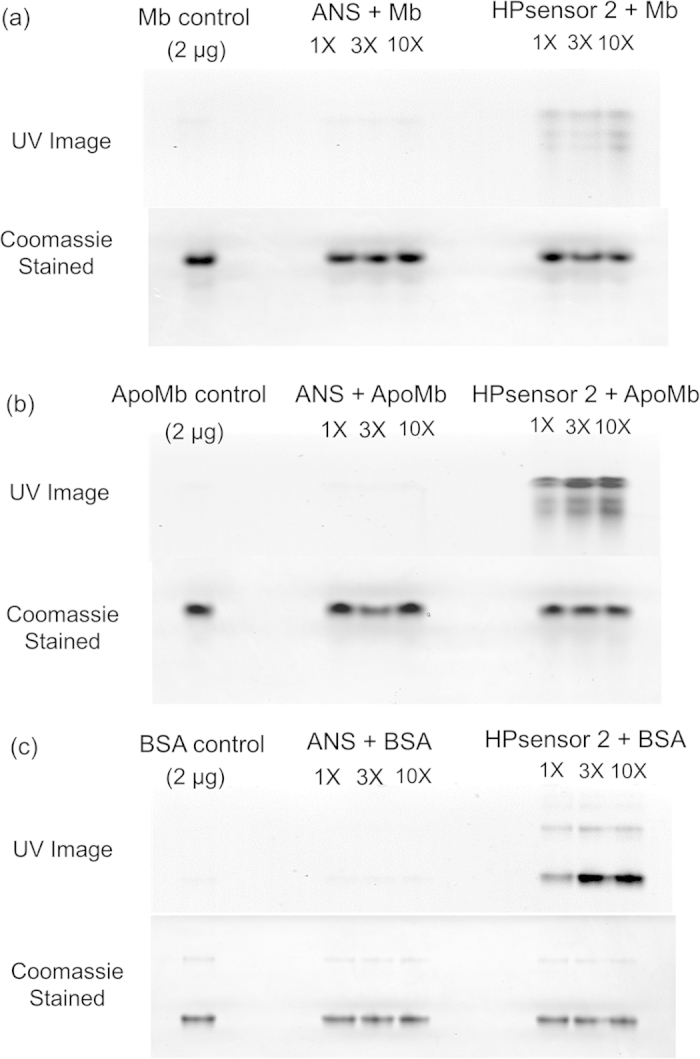
Native PAGE of 2 μg proteins with 1X, 3X, and 10X Dye (ANS or HPsensor 2). 2 μg of proteins Mb (**a**), ApoMb (**b**), and BSA (**c**) were incubated with 1X, 3X, and 10X concentration of dyes (ANS or HPsensor **2**) for 1 h at 25 °C. The BSA protein was run on a 10% gel for 3 h and Mb and ApoMb proteins were run on a 15% gel for 6 h at 80 V. Full length gels are included in supplementary figures 30–32. Brightness and contrast settings of gels were adjusted for aesthetic purposes.

Finally, we tested the three proteins Mb, ApoMb, and BSA with ANS and HPsensor **2** on a native PAGE. The UV gel image showed that HPsensor **2** exhibited a much stronger signal than ANS (Fig. 6) upon UV exposure for all three proteins. It was interesting to see that HPsensor **2** showed decreased signal with ApoMb and the least signal with Mb after exposure to UV light (Supplementary Fig. 18) which is in line with the fluorescence data.

## Discussion

The novel BODIPY dyes with aryl substitutions (with NH_2_, NHAc, or OCH_3_ groups) at the *meso* position for sensing protein hydrophobicity and 2-methoxyethylamine substitution at the 3,5-positions for increasing water solubility were synthesized. The **control** dye (nitroaryl substitution at *meso* position) has been reported in an earlier study and is known to have weak fluorescence^34^^,11c^ primarily due to free rotation of the nitroaryl group resulting in high non-radiative decay rate unlike the methylaryl counterpart that gives quantum yields of 63%^33^.

**Figure 7:**
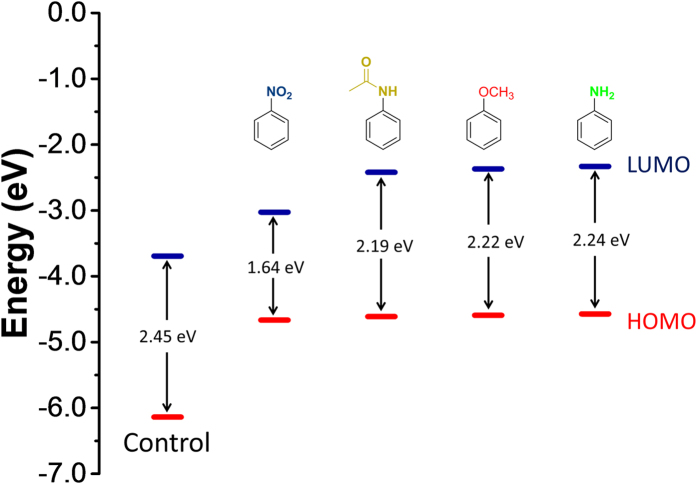
Schematic energy level diagrams of frontier molecular orbitals of control dye, dye 5, and HPsensors (1, 2, and 3) showing their HOMO-LUMO energy gap (eV) in ethanol. The data were taken from Supplementary Table 2.

With previous literature suggesting that the *meso* aryl substitution has a profound effect on fluorescence characteristics irrespective of the lack of π-conjugation^35^, we sought to investigate the role of the electron donating ability on fluorescence. The three donor groups (NH_2_, NHAc, and OCH_3_), substituted to aryl group at the *meso* position have been known to cause an enhancement in fluorescence^36^ and thus served as an important starting point for our dyes. We used the 3,5-positions for 2-methoxyethylamine substitution to increase stability and solubility of **control** dye in polar environment by enhancing the hydrogen bonding ability. Interestingly, addition of the 2-methoxyethylamine groups to the **control** dye led to quenching of fluorescence as noted for dye **5** (Fig. 1). The fluorescence quenching may be due to photo-induced electron transfer with nitrophenyl group functioning as an electron acceptor. While decrease in fluorescence quantum yield was expected due to free rotation of aryl substituents at the *meso* position by non-radiative decay processes (k_nr_)^28^, a total loss of fluorescence (quenching) was unexpected. Quantum mechanical calculations for HOMO and LUMO gap by first-principle density functional theory showed a significant decrease in HOMO-LUMO gap for dye 5 (1.639 eV) compared to **control** dye (2.445 eV) (Fig. 7 and Supplementary Table 2). This decrease in HOMO-LUMO gap due to 2-methoxyethylamine substitution at 3,5-positions in combination with free rotation of nitroaryl substituent at *meso* position can account for quenching of fluorescence for the dye **5**. However, the other substitutions (NH_2_, NHAc, and OCH_3_) showed a significant increase in fluorescence of the HPsensors upon binding to hydrophobic proteins (ApoMb and BSA) (Figs 3 and 4). The HPsensors **1**, **2**, and **3** showed a red-shift in excitation (561—569 nm) and emission (577–587 nm) compared to the **control** dye (Ex 517 nm and Em 540 nm), with increase in fluorescence quantum yield (Φ_f_) in different solvents (Supplementary Table 1). This shift in excitation and emission maxima towards longer wavelength with decreasing solvent polarity could be due to slight decrease (~0.22 eV) in the energy gap for HPsensors (Fig. 7 and Supplementary Table 2) compared to **control** dye leading to increased conjugation of π-system of the chromophore^37^.

**Figure 8:**
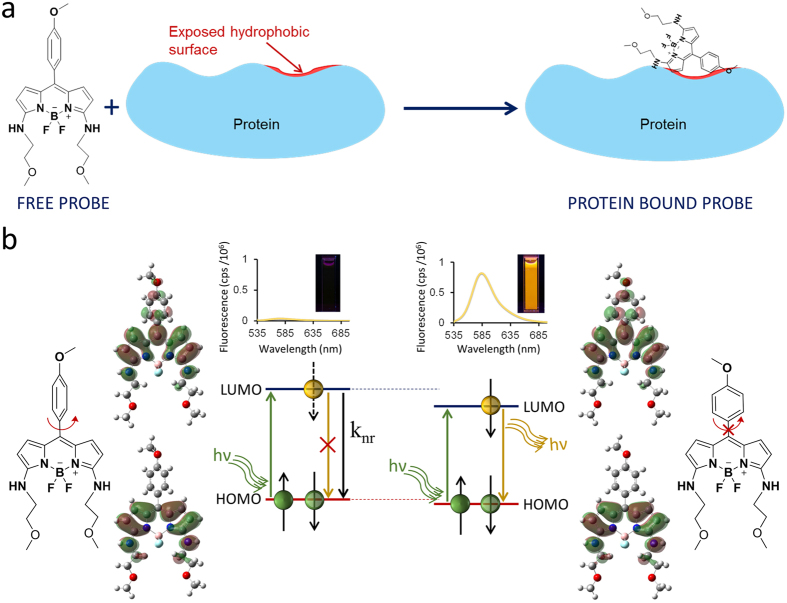
Plausible model for increase in fluorescence of HPsensors. (**a**) Cartoon shows that free HPsensor **2** has very weak fluorescence in aqueous environment. However, upon binding to proteins HPsensor **2** shows marked enhancement in fluorescence due to binding of dye to proteins hydrophobic surface resulting in molecular twisting and increased rigidity due to steric hindrance; (**b**) Molecular mechanism shows that *meso* aryl substitution in ethanol can twist resulting in decrease in HOMO and LUMO gap; that combined with increased rigidity of dye inhibits free rotation of aryl substituents leading to decrease in non-radiative decay. This decrease in HOMO and LUMO gap combined with increased molecular rigidity leads to enhancement in fluorescence.

The amphiphilic nature of HPsensors is critical for surface hydrophobicity measurements in proteins as surface hydrophobic regions on proteins are exposed to solvent (aqueous) and require a balance of hydrophobic as well as hydrophilic interaction for achieving efficient binding of dye. The results show that increasing the electron donating ability of substituent aryl groups enhances the hydrophobic sensing of the HPsensors and help differentiate the degree of hydrophobicity in proteins. BSA had the highest level of surface hydrophobicity, followed by ApoMb and then Mb as measured by HPsensors (Fig. 3). This increase in fluorescence of HPsensors can be attributed to aryl substituents^38^ (with NH_2_, NHAc, or OCH_3_ groups) restricting free rotation at the *meso* position. In addition, increased rigidity of dye due to binding of ring structure to protein’s hydrophobic surface and increased hydrogen bonding of 2-methoxyethylamine group with aqueous phase can reduce non-radiative deactivation resulting in fluorescence enhancement (Fig. 8)^39^. In addition, HPsensor **2** showed remarkable reporting ability of hydrophobicity with signal strength 10- to 60-fold higher compared to ANS when tested with Mb, ApoMb and BSA under identical conditions (Fig. 4 and Supplementary Fig. 13). We evaluated the relative surface hydrophobicity of the three proteins (Mb, ApoMb, and BSA) using HPsensor **2** and showed BSA to be the most hydrophobic and Mb to be the least hydrophobic (Supplementary Fig. 17). In addition, evaluation of the surface electrostatic and hydrophobic maps for proteins using computational modeling software SPDB^40^ showed BSA to be the most hydrophobic (Supplementary Fig. 19–22) compared to the other proteins tested. However, the difference in calculated hydrophobicity for Mb and ApoMb is negligible (Supplementary Fig. S19 and S20), suggesting limitations of such calculations and delineation from the experimental evidence^41^,^42^,^43^. Independent studies show that ApoMb is partially unfolded and more flexible due to loss of heme group resulting in loosening of helical structure when compared to Mb^41^,^42^. Therefore, this loosening of structure due to loss of metal ion can lead to increase in aberrant surface hydrophobicity of ApoMb in a manner similar to that seen for other metalloproteins^12^.

To further evaluate the strength of the hydrophobic interaction between HPsensor **2** and proteins, we carried out native PAGE. Due to the large difference in isoelectronic point of BSA (pI ~ 4.5) and ApoMb/Mb (pI ~ 7.5 – 8.5), and their size, the amount of time required to sufficiently resolve proteins on the respective cross-linked percentage gels (10% for BSA and 15% for ApoMb and Mb) were adjusted accordingly. With BSA, ApoMb and Mb, the signal strength of HPsensor **2** was much greater than that of ANS under similar conditions as seen by UV imaging (Fig. 6). In addition, the signal intensity increased and was in line with the predicted level of exposed surface hydrophobicity of these proteins with BSA showing the highest hydrophobicity (Supplementary Fig. 18).

We also evaluated the response of the most sensitive dye, HPsensor **2**, to change in surface polarity of BSA and β-lg upon heating and compared it to unheated proteins at different pHs (Fig. 5). Thermal denaturation of proteins at different pHs^15^,^44^,^45^ have been shown to influence the extent of surface hydrophobic exposure of BSA and β-lg as measured by dyes such as PRODAN and ANS^15^. ANS being an anionic probe overestimates hydrophobicity at acidic pH due to electrostatic interactions whereas PRODAN being uncharged is not influenced by changes in pH^15^. Our results show that HPsensor **2** was more responsive to change in surface hydrophobicity (Fig. 5). Furthermore, these surface hydrophobicity measurements of BSA and β-lg by HPsensor **2** are in line with the uncharged dye PRODAN. Considering all the properties of the dyes above, HPsensor **2** is an ideal dye for evaluating protein surface hydrophobicity (*S*_*0*_) and can be used as a sensitive hydrophobic probe for proteins.

## Conclusion

We report novel HPsensors for mapping proteins surface hydrophobicity that show a 10- to 60-fold stronger signal compared to commonly used fluorophore ANS with affinity for proteins in the nanomolar range. The strong signal to noise ratio suggests that these dyes can be useful for applications even with a minute quantity of hydrophobic protein. Thus, this work provides a framework for synthesis of future amphiphilic dyes that can be used for specifically reporting protein surface hydrophobicity with higher sensitivity. We expect these dyes in combination with other techniques such as reverse phase-high performance liquid chromatography (RP-HPLC) have the potential for characterizing protein surface properties. This will help us better understand protein-ligand interactions, molecular recognition, and their biological functions.

## Methods

### Materials

Unless otherwise indicated, all reagents and solvents were obtained from commercial suppliers (Sigma and Fisher) and used without further purification. Protein samples of BSA and equine myoglobin were purchased from Sigma. ApoMb was prepared from equine myoglobin (Sigma) as per a modified protocol of Breslow (1965)^46^ and Adams (1977)^47^ outlined in supplementary information.

### Instrumentation

^1^H NMR and ^13^C NMR spectra were procured on a 400 MHz Varian Unity Inova spectrophotometer instrument. FTIR spectra were acquired using a Perkin Elmer Spectrum One FTIR. UV spectra were measured using the Perkin Elmer Lambda 35 UV/VIS spectrometer and the fluorescence spectra were measured using the Jobin Yvon Fluoromax-4 spectrofluorometer.

### Spectroscopic studies

Fluorescence quantum yields of BODIPY dyes were measured in dichloromethane, ethanol and water and calculated using the previously reported method^30^ outlined in the supporting methods. The sulforhodamine 101 dye (Φ_n_ = 95% using an excitation wavelength of 577 nm in ethanol)^48^ was used as the fluorescence standard to measure fluorescence quantum yields of the new BODIPY dyes. The absorption spectra were measured from 300 nm to 800 nm in applicable solvents at 1 nm intervals. The emission spectra for fluorescent dyes were measured at 1 nm intervals using excitation wavelengths of 520 nm for HPsensors **1** and **3** and 528 nm for HPsensor **2** with both excitation and emission band widths at 2 nm.

The absorption and emission spectra of 2 μM of each BODIPY dye were acquired in ethanol-water mixture with increasing concentration of ethanol (20% increments ranging from 0 to 100% ethanol) to check their fluorescence sensitivity to change in solvent polarity. These dyes were also investigated for their pH sensitivity using Carmody buffer series^49^ from pH 2 to pH 12. Dyes were incubated at a concentration of 2 μM in increasing pH for 30 mins after which the emission spectra were acquired in triplicate. To check for dye stability with pH, fluorescence of 2 μM of dyes with change in pH from 3 to 8 and then back to pH 3 was measured using 5 M NaOH and 5 M HCl, respectively. Extinction coefficient was calculated for each dye in 100% ethanol using increasing dye concentrations from 5 μM to 30 μM.

In addition, the sensitivity of these dyes were investigated for ions (Na^+^, Mg^2+^, Zn^2+^, Fe^2+^, Fe^3+^, Ca^2+^) commonly found in aqueous solutions and buffers. The fluorescence emission spectra of dyes at 2 μM concentrations in distilled water (adjusted to pH 8 using 0.1 M NaOH) with increasing concentration of each test ion (0 to 150 μM) at room temperature was acquired in triplicate. For experiments with proteins, dye concentration dependence was investigated by measuring florescence of increasing concentration of dyes (0 to 100 μM range) in the presence and absence of 2 μM BSA. All proteins were tested in water (adjusted to pH 8 using 0.1 M NaOH) because ApoMb is prone to aggregation in buffer salts. Fluorescence emission spectra of dyes with all three proteins (Mb, ApoMb, and BSA) were collected by incubating 2 μM dyes with 2 μM proteins (1:1 ratio) for 1 hour before acquiring the absorption and emission spectra. ANS dye was similarly tested at 2 μM concentration with Mb, ApoMb and BSA for comparison to these BODIPY based dyes (Supplementary Fig. 13). All protein and dye samples were freshly prepared and incubated at room temperature for 1 h before acquiring the fluorescence spectra. All spectra were plotted using OriginPro 9.1 and schemes were drawn using ChemBioDraw 14.

### Surface Hydrophobicity and Binding Affinity

Surface hydrophobicity (*S0*) measurements and binding affinity of proteins (Mb, ApoMb and BSA) were determined using HPsensor **2** as per established protocol^5^,^45^. For the *S0* measurements, 0.5 μM HPsensor **2** in distilled water (adjusted to pH 8 using 0.1 M NaOH) was incubated with 8–10 concentrations of each test protein (0.1–1 μM for Mb; 0.002–0.1 μM for ApoMb; and 0.006–0.1 μM for BSA) for 1 h at 25 °C before acquiring the emission spectra. The net relative fluorescence intensity (RFI) was then calculated by subtracting the fluorescence of protein in water from protein + HPsensor **2**. The slope (linear regression fit) of net RFI (at 579 nm) vs protein concentration gave the surface hydrophobicity of each protein (Supplementary Fig. 17). To measure the binding affinity of HPsensor **2** for the proteins, fluorescence titration curves were acquired using 21–28 different concentrations of protein in the presence of 0.5 μM HPsensor **2**. The range of protein concentrations used were: 0.1–3.0 μM for Mb, 0.002–3 μM for ApoMB, and 0.006–8.5 μM for BSA. The data was analyzed by a non-linear regression method using the MichaelisMenten model included in OriginPro 9.1. Finally, for evaluating the probe sensitivity to increasing polarity on protein’s surface, an established protocol^15^ was used with the following modification. The fluorescence of bound protein/dye was plotted against pH as opposed to the surface hydrophobicity. The proteins were prepared in 2 forms: either heated (80 °C for 30 mins) or unheated for analysis. To begin with, 0.1 μM of each protein (heated/unheated BSA or β-lg) tested was incubated with HPsensor **2** (0.5 μM) for ~30 mins in Carmody buffer series^49^ at pH 3, 5, 7 or 9. Bound protein/dye fluorescence was determined by the difference of fluorescence for protein + HPsensor **2** to protein alone at 579 nm. All spectra were plotted using OriginPro 9.1.

### Native PAGE of proteins

2 μg each of BSA, ApoMb, and Mb proteins were incubated in the presence of increasing concentration (1X, 3X, and 10X) of dyes (ANS and HPsensor **2**) for 1 h at room temperature. In addition, 5 μg of BSA was also incubated with the two dyes (ANS and HPsensor **2**) at increasing concentration (1X, 5X. and 25X) for 1 h at room temperature. Proteins incubated with dye were then mixed (1:1) with native sample buffer before polyacrylamide gel electrophoresis (PAGE) at 80 V. The proteins were run on different percentage gels for separation by electrophoresis on native PAGE. BSA protein was run on a 10% gel for 3 h and ApoMb and Mb were run on a 15% gel for 6 h. UV images of gels were acquired using the Bio Doc-It imaging system before staining with Coomassie blue.

### Surface Electrostatic and Hydrophobic Molecular Modeling

In order to evaluate the differences between proteins surface properties used in this study, surface electrostatic maps were generated for Mb (PDB ID: 3RJ6), ApoMb (Modified from 3RJ6), beta lactoglobulin (PDB ID: 2Q2M) and BSA (PDB ID: 3V03) using the APBS software (http://www.poissonboltzmann.org/) at pH 8^50^,^51^,^52^. This was then displayed using the included web viewer Jmol_S. In addition, the Swiss-Prot software SPDB (http://spdbv.vital-it.ch/) was used to generate surface hydrophobic maps for each of these proteins^40^.

### Computational methods

To identify the mechanism responsible for the selective enhancement of fluorescence behavior of the HPsensors, we have used a first-principles density functional theory (DFT)^53^ that employs a range separated hybrid functional HSEH1PBE for the exchange and correlation^54^ to carry out the electronic structure calculations. This functional has been used recently to study the electronic structure of various materials including organic molecules^55^,^56^. An all electron Gaussian basis set^54^, 6–311g**, is used for the calculations. To include the solvent effect due to water or ethanol, we have used a polarizable continuum model (PCM) using Gaussian 09 suite program^54^.

## Additional Information

**How to cite this article**: Dorh, N. *et al.* BODIPY-Based Fluorescent Probes for Sensing Protein Surface-Hydrophobicity. *Sci. Rep.* **5**, 18337; doi: 10.1038/srep18337 (2015).

## Supplementary Material

Supplementary Information

## References

[b1] Dill K. A. Dominant Forces in Protein Folding Biochemistry 29, 7133–7154 (1990). 2207096 10.1021/bi00483a001

[b2] Malleshappa Gowder S., Chatterjee J., Chaudhuri T. & Paul K. Prediction and analysis of surface hydrophobic residues in tertiary structure of proteins. Scientific World Journal 2014, 971258, doi: 10.1155/2014/971258 (2014). 24672404 PMC3930195

[b3] Nick Pace C., Scholtz J. M. & Grimsley G. R. Forces stabilizing proteins. FEBS Lett. 588, 2177–2184, doi: 10.1016/j.febslet.2014.05.006 (2014). 24846139 PMC4116631

[b4] Jamadagni S. N., Godawat R. & Garde S. In Annual Review of Chemical and Biomolecular Engineering, Vol. 2 Annual Review of Chemical and Biomolecular Engineering (ed Prausnitz J. M.) 147–171 (2011). 10.1146/annurev-chembioeng-061010-114156 22432614

[b5] Haskard C. A. & Li-Chan E. C. Y. Hydrophobicity of Bovine Serum Albumin and Ovalbumin Determined Using Uncharged (PRODAN) and Anionic (ANS-) Fluorescent Probes. J. Agric. Food Chem. 46, 2671–2677 (1998).

[b6] Young L., Jernigan R. L. & Covell D. G. A role for surface hydrophobicity in protein-protein recognition. Protein Sci. 3, 717–729 (1994). 8061602 10.1002/pro.5560030501 PMC2142720

[b7] Wang B., Guo C., Lou Z. & Xu B. Following the aggregation of human prion protein on Au(111) surface in real-time. Chem. Commun. (Cambridge, U.K.) 51, 2088–2090, doi: 10.1039/c4cc09209k (2015). 25535923

[b8] Xu G., Stevens S. M. Jr., Moore B. D., McClung S. & Borchelt D. R. Cytosolic proteins lose solubility as amyloid deposits in a transgenic mouse model of Alzheimer-type amyloidosis. Hum. Mol. Genet. 22, 2765–2774, doi: 10.1093/hmg/ddt121 (2013). 23512986 PMC3690965

[b9] Knowles T. P. J., Vendruscolo M. & Dobson C. M. The amyloid state and its association with protein misfolding diseases. Nat. Rev. Mol. Cell. Biol. 15, 384–396, doi: 10.1038/nrm3810 (2014). 24854788

[b10] Ross C. A. & Poirier M. A. Protein aggregation and neurodegenerative disease. Nat. Med. 10, S10–17, doi: 10.1038/nm1066 (2004). 15272267

[b11] Tiwari A., Xu Z. & Hayward L. J. Aberrantly Increased Hydrophobicity Shared by Mutants of Cu,Zn-Superoxide Dismutase in Familial Amyotrophic Lateral Sclerosis. J. Biol. Chem. 280, 29771–29779, doi: 10.1074/jbc.M504039200 (2005). 15958382

[b12] Tiwari A. *et al.* Metal deficiency increases aberrant hydrophobicity of mutant superoxide dismutases that cause amyotrophic lateral sclerosis. J. Biol. Chem. 284, 27746–27758, doi: 10.1074/jbc.M109.043729 (2009). 19651777 PMC2785702

[b13] Adams M. M. & Anslyn E. V. Differential Sensing Using Proteins: Exploiting the Cross-Reactivity of Serum Albumin To Pattern Individual Terpenes and Terpenes in Perfume. J. Am. Chem. Soc. 131, 17068–17069 (2009). 19904949 10.1021/ja908319m

[b14] Lee S. *et al.* Rational design of a structural framework with potential use to develop chemical reagents that target and modulate multiple facets of Alzheimer’s disease. J. Am. Chem. Soc. 136, 299–310, doi: 10.1021/ja409801p (2014). 24397771 PMC4096303

[b15] Alizadeh-Pasdar N. & Li-Chan E. C. Y. Comparison of Protein Surface Hydrophobicity Measured at Various pH Values Using Three Different Fluorescent Probes. J. Agric. Food Chem. 48, 328–334 (2000). 10691636 10.1021/jf990393p

[b16] Cardamone M. & Puri N. K. Spectrofluorimetric assessment of the surface hydrophobicity of proteins. Biochem. J. 282, 589–593 (1992). 1546973 10.1042/bj2820589 PMC1130822

[b17] Scarsi M., Majeux N. & Caflisch A. Hydrophobicity at the Surface of Proteins. Proteins: Struct., Funct., Bioinf. 37, 565–575 (1999). 10.1002/(sici)1097-0134(19991201)37:4<565::aid-prot7>3.0.co;2-v 10651272

[b18] Balch W. E., Fau M. R., Fau D. A. & Kelly J. W. Adapting proteostasis for disease intervention. Science 319, 916–919, doi: 10.1126/science.1141448 (2008). 18276881

[b19] Giovambattista N., Lopez C. F., Rossky P. J. & Debenedetti P. G. Hydrophobicity of protein surfaces: Separating geometry from chemistry. Proc. Natl. Acad. Sci. USA 105, 2274–2279, doi: 10.1073/pnas.0708088105 (2008). 18268339 PMC2268126

[b20] Raschke T. M., Tsai J. & Levitt M. Quantification of the hydrophobic interaction by simulations of the aggregation of small hydrophobic solutes in water. Proc. Natl. Acad. Sci. USA 98, 5965–5969, doi: 10.1073/pnas.111158498 (2001). 11353861 PMC33406

[b21] Saito R. *et al.* Peptide-conjugated pterins as inhibitors of ricin toxin A. J. Med. Chem. 56, 320–329, doi: 10.1021/jm3016393 (2013). 23214944 PMC3552522

[b22] Hawe A., Sutter M. & Jiskoot W. Extrinsic fluorescent dyes as tools for protein characterization. Pharm. Res. 25, 1487–1499, doi: 10.1007/s11095-007-9516-9 (2008). 18172579 PMC2440933

[b23] Peng L. *et al.* A ratiometric fluorescent probe for hydrophobic proteins in aqueous solution based on aggregation-induced emission. Analyst 138, 2068–2072, doi: 10.1039/c3an36634k (2013). 23435163

[b24] Gasymov O. K. & Glasgow B. J. ANS Fluorescence: Potential to Augment the Identification of the External Binding Sites of Proteins. Biochim. Biophys. Acta 1774, 403–411 (2007). 17321809 10.1016/j.bbapap.2007.01.002 PMC2039916

[b25] Togashi D. M. & Ryder A. G. A fluorescence analysis of ANS bound to bovine serum albumin: binding properties revisited by using energy transfer. J. Fluoresc. 18, 519–526, doi: 10.1007/s10895-007-0294-x (2008). 18097738

[b26] Matulis D. & Lovrien R. 1-Anilino-8-Naphthalene Sulfonate Anion-Protein Binding Depends Primarily on Ion Pair Formation. Biophys. J. 74 422–429 (1998). 9449342 10.1016/S0006-3495(98)77799-9 PMC1299394

[b27] Li L., Nguyen B. & Burgess K. Functionalization of the 4,4-difluoro-4-bora-3a,4a-diaza-s-indacene (BODIPY) core. Bioorg. Med. Chem. Lett. 18, 3112–3116, doi: 10.1016/j.bmcl.2007.10.103 (2008). 18037291 PMC2430868

[b28] Loudet A. & Burgess K. BODIPY Dyes and Their Derivatives: Syntheses and Spectroscopic Properties. Chem. Rev. 107, 4891–4932 (2007). 17924696 10.1021/cr078381n

[b29] Zhang Y. *et al.* Photoactivatable BODIPYs Designed To Monitor the Dynamics of Supramolecular Nanocarriers. J. Am. Chem. Soc. 137, 4709–4719, doi: 10.1021/ja5125308 (2015). 25794143

[b30] Zhu S. *et al.* Highly water-soluble neutral near-infrared emissive BODIPY polymeric dyes. J. Mater. Chem. 22, 2781–2790, doi: Doi 10.1039/C2jm14920f (2012).

[b31] Zhu S. *et al.* Highly water-soluble, near-infrared emissive BODIPY polymeric dye bearing RGD peptide residues for cancer imaging. Anal. Chim. Acta 758, 138–144, doi: 10.1016/j.aca.2012.10.026 (2013). 23245906 PMC3527847

[b32] Zhu S. *et al.* Highly water-soluble BODIPY-based fluorescent probes for sensitive fluorescent sensing of zinc(II). J. Mater. Chem. B 1, 1722, doi: 10.1039/c3tb00249g (2013). 32260703

[b33] Leen V. *Synthesis and application of reactive BODIPY dyes* PhD thesis, Katholieke Universiteit Leuven, (2010).

[b34] Li L., Han J., Nguyen B. & Burgess K. Syntheses and spectral properties of functionalized, water-soluble BODIPY derivatives. J. Org. Chem. 73, 1963–1970, doi: 10.1021/jo702463f (2008). 18271598

[b35] Zhang X.-F. The effect of phenyl substitution on the fluorescence characteristics of fluorescein derivatives via intramolecular photoinduced electron transfer. Photochem. Photobiol. Sci. 9, 1261, doi: 10.1039/c0pp00184h (2010). 20714676

[b36] Skoog D. A., Holler F. J. & Crouch S. R. Principles of Instrumental Analysis. 357 ( Thomson Brooks/Cole, 2007).

[b37] Baruah M., Qin W., Basaric N., Borggraeve W. M. D. & Boens N. BODIPY-Based Hydroxyaryl Derivatives as Fluorescent pH Probes. J. Org. Chem. 70, 4152–4157 (2005). 15876108 10.1021/jo0503714

[b38] Yamada K., Toyota T., Takakura K., Ishimaru M. & Sugawara T. Preparation of BODIPY probes for multicolor fluorescence imaging studies of membrane dynamics. New J. Chem. 25, 667–669, doi: Doi 10.1039/B100757m (2001).

[b39] Benniston A. C. & Copley G. Lighting the way ahead with boron dipyrromethene (BODIPY) dyes. Phys Chem Chem Phys 11, 4124–4131, doi: 10.1039/b901383k (2009). 19458813

[b40] Guex N. & Peitsch M. C. SWISS-MODEL and the Swiss-Pdb Viewer: An environment for comparative protein modeling. ELECTROPHORESIS 18, 2714–2723, doi: 10.1002/elps.1150181505 (1997). 9504803

[b41] Harrison S. C. & Blout E. R. Reversible conformational changes of myoglobin and apomyoglobin. J. Biol. Chem. 240, 299–303 (1965). 14253427

[b42] Lee D. W. & Cho B. Y. Investigation of the Retention Behavior and Structural Change of Proteins in Reversed Phase and Hydrophobic Interaction Chromatography. J. Liq. Chromatogr. 17, 2541–2558, doi: 10.1080/10826079408013396 (1994).

[b43] Dreyer S., Salim P. & Kragl U. Driving forces of protein partitioning in an ionic liquid-based aqueous two-phase system. Biochem. Eng. J. 46, 176–185, doi: 10.1016/j.bej.2009.05.005 (2009).

[b44] Kato A. & Nakai S. Hydrophobicity determined by a fluorescence probe method and its correlation with surface properties of proteins. Biochim. Biophys. Acta 624, 13–20 (1980). 7407231 10.1016/0005-2795(80)90220-2

[b45] Nakai S., Li-Chan E. & Arteaga G. E. In Methods of testing protein functionality (ed Hall G. M.) Ch. 8, 226–255 (Blackie A & P, 1996).

[b46] Breslow E. Changes in Side Chain Reactivity Accompanying the Binding of Heme to Sperm Whale Apomyoglobin. J. Biol. Chem. 239, 486–496 (1964). 14169149

[b47] Adams P. A. The Kinetics of the Recombination Reaction between Apomyoglobin and Alkaline Haematin. Biochem. J. 163, 153–158 (1977). 17391 10.1042/bj1630153 PMC1164671

[b48] Velapoldi R. A. & Tønnesen H. H. Corrected Emission Spectra and Quantum Yields for a Series of Fluorescent Compounds in the Visible Spectral Region. J. Fluoresc. 14, 465–472 (2004). 15617389 10.1023/b:jofl.0000031828.96368.c1

[b49] Carmody W. R. An Easily Prepared Wide Range Buffer Series. J. Chem. Educ. 38, 559–560 (1961).

[b50] Baker N. A., Sept D., Joseph S., Holst M. J. & McCammon J. A. Electrostatics of nanosystems: application to microtubules and the ribosome. Proc. Natl. Acad. Sci. USA 98, 10037–10041 (2001). 11517324 10.1073/pnas.181342398 PMC56910

[b51] Dolinsky T. J. *et al.* PDB2PQR: expanding and upgrading automated preparation of biomolecular structures for molecular simulations. Nucleic acids research 35, W522–525, doi: 10.1093/nar/gkm276 (2007). 17488841 PMC1933214

[b52] Dolinsky T. J., Nielsen J. E., McCammon J. A. & Baker N. A. PDB2PQR: an automated pipeline for the setup of Poisson-Boltzmann electrostatics calculations. Nucleic acids research 32, W665–667, doi: 10.1093/nar/gkh381 (2004). 15215472 PMC441519

[b53] Parr R. G. & Yang W. Density-functional theory of atoms and molecules. Vol. 16 (Oxford University Press, 1994).

[b54] Frisch M. J. *et al.* GAUSSIAN09, Gaussian, Inc., Wallingford, CT, USA, (2009), Available at: http://www.gaussian.com/g_tech/g_ur/m_citation.htm. (Accessed: 4th September 2015).

[b55] Chevrier V. L., Ong S. P., Armiento R., Chan M. K. Y. & Ceder G. Hybrid density functional calculations of redox potentials and formation energies of transition metal compounds. Phys. Rev. B: Condens. Matter Mater. Phys. 82, 1–11, doi: 10.1103/PhysRevB.82.075122 (2010).

[b56] Tamer Ö. *et al.* Synthesis, structural and spectroscopic evaluations and nonlinear optical properties of 3,5-bis(4-methoxyphenyl)-4,5-dihydro-1H-pyrazole-1-carbothioic O-acid. Spectrochim. Acta, Part A, doi: 10.1016/j.saa.2014.08.111 (2014). 25306134

