# Supplementary material for: BODIPY-Based Fluorescent Probes for Sensing Protein Surface-Hydrophobicity
Source: Sci Rep. 2015 Dec 18;5:18337. doi: 10.1038/srep18337 (PMC4683377; doi:10.1038/srep18337)
Supplement: Supplementary Information [file srep18337-s1.pdf]

Supplementary Information

BODIPY-Based Fluorescent Probes for Sensing Protein Surface-Hydrophobicity

Nethaniah Dorh[a], Shilei Zhu[a,†], Kamal B. Dhungana[b], Ranjit Pati[b], Fen-Tair Luo[c], Haiying Liu[a], Ashutosh Tiwari[a]*.

[a]Department of Chemistry, Michigan Technological University, Houghton, MI 49931, USA

[b]Department of Physics, Michigan Technological University, Houghton, MI 49931, USA

[c]Institute of Chemistry, Academia Sinica, Taipei, Taiwan 11529, Republic of China

[†]Department of Chemistry & Biochemistry, University of Maryland, College Park, MD 20742, USA

*Corresponding Author: tiwari@mtu.edu

Supplementary Information/Figures	Page
**I. Experimental**	**S3 – S5**
Apomyoglobin preparation	S3
Dye Synthesis	S3-S4
Quantum yield determination of dyes	S5
Quantum yield and extinction coefficients (Table 1)	S5
**II. Emission spectra of dyes in ethanol-water dilutions** (Figure 1)	S6
**III. Normalized spectra of dyes in ethanol, water and dichloromethane**	**S7-S12**
Normalized Spectra of HPsensor **1** (Figure 2a-d)	S7-S8
Normalized Spectra of HPsensor **2** (Figure 3a-d)	S9-S10
Normalized Spectra of HPsensor **3** (Figure 4a-d)	S11-S12
**IV. Absorption Spectra of dyes in ethanol-water dilutions** (Figure 5a-d)	**S13-S14**
**V. Dye sensitivity to pH** (Figure 6)	**S15**
**VI. Mean fluorescence of dyes with changing pH** (Figure 7)	**S16**
**VII. Bar graphs of Dyes with ions (Mg^{2+}, Ca^{2+}, Fe^{2+}, Fe^{3+}, Na^{+}, Zn^{2+})** (Figure 8)	**S17**
**VIII. Mean fluorescence of increasing concentration of dyes in presence and absence of BSA** (Figure 9)	**S18**
**IX. Emission spectra of HPsensor 1, 2 and 3 with proteins** (Figure 10 - 12)	**S19 – S21**
**X. ANS and HPsensor 2 with Proteins** (Figure 13)	**S22**
**XI. Normalized emission spectra of dyes with proteins** (Figure 14)	**S23**
**XII. Native PAGE of 5 μg BSA with ANS and HPsensor 2** (Figure 15)	**S24**

Supplementary Information/Figures	Page
**XIII. Binding affinity of test proteins (Mb, ApoMb, BSA)** (Figure 16)	**S25**
**XIV. Surface hydrophobicity of test proteins (Mb, ApoMb, BSA)** (Figure 17)	**S26**
**XV. Native PAGE of 2 µg proteins with HPsensor 2** (Figure 18)	**S27**
**XVI. Electrostatic and Hydrophobic patch maps of Myoglobin** (Figure 19)	**S28**
**XVII. Electrostatic and Hydrophobic patch maps of Apomyoglobin** (Figure 20)	**S29**
**XVIII. Electrostatic and Hydrophobic patch maps of beta lactoglobulin** (Figure 21)	**S30**
**XIX. Electrostatic and Hydrophobic patch maps of Bovine Serum Albumin** (Figure 22)	**S31**
**XX. HOMO-LUMO images of dyes in ethanol** (Figure 23)	**S32**
**XXI. HOMO-LUMO images of dyes in water** (Figure 24)	**S33**
**XXII. HOMO-LUMO energy gap calculation of dyes using (HSEH1PBE) and 6-311g** basis.** (Table 2 –3)	**S34**
**XXIII. ^{1}H and ^{13}C NMR spectra of dyes** (Figure 25 – 29)	**S35-S44**
**XXIV. Full Length Native gels of proteins with ANS and HPsensor 2** (Figure 30 – 34)	**S45 – S49**
Full length gel of Mb (2 µg) (Figure 30)	S45
Full length gel of ApoMb(2 µg) (Figure 31)	S46
Full length gel of BSA (2 µg) (Figure 32)	S47
Full length gel of BSA (5 µg) (Figure 33)	S48
Full length gel of Mb, ApoMb and BSA (2 µg) on 10% gel (Figure 34)	S49
**XXV. References**	**S50**

Apomyoglobin preparation:

Myoglobin was dissolved in water at (1 to 3 % w/v) and then incubated 4 °C. To the solution, 1 M HCl was added until pH 2.0 was achieved. Then an equal volume of -20 °C 2-butanone was added followed by thorough mixing. After phase separation at 4 °C the top layer of ketone supernatant containing heme was then removed and discarded. This was repeated two more times until the remaining solution was pale yellow to whitish. The solution was then dialyzed against buffer for a total of nine washes using the Spectra/Por 7 dialysis tubing, 6-8K MWCO. Protein concentration was determined using absorbance at 280 nm (ε_{280} equine apomyoglobin: 15,700 $M^{-1}cm^{-1}$).[1]

Buffer solutions used to dialyze apomyoglobin solution in order of progression:

- Deionized MQ water (pH 2.5, adjusted with 1M HCl; 1 mM EDTA) at 4°C for 1 h and then repeat for 2 h.
- 20 mM glycine HCl buffer (pH 2.5) with 2 mM EDTA at 4°C for 4 h.
- 20 mM glycine HCl buffer (pH 2.5) with 2 mM EDTA at 4°C for 8 h.
- 20 mM glycine HCl buffer (pH 2.5; with chelex) at 4°C for 1 h initially and then for 6 h for two runs.
- 20 mM citrate buffer at pH 5.5 with chelex at 4°C.
- Deionized MilliQ water overnight.

Dye Synthesis

Instrumentation and materials. ^{1}H NMR and ^{13}C NMR spectra were taken on a 400 MHz Varian Unity Inova spectrophotometer instrument. ^{1}H and ^{13}C NMR spectra were recorded in $CDCl_3$, chemical shifts (δ) are given in ppm relative to solvent peaks (^{1}H: δ 7.26; ^{13}C: δ 77.3) as internal standard. Unless otherwise indicated, all reagents and solvents were obtained from commercial suppliers (Aldrich, Sigma, Fluka, Acros Organics, Fisher Scientific, and Lancaster) and used without further purification.

Compound 1 was prepared according to a reported procedure (J. Org. Chem., 2008, 73 (5), 1963–1970). ^{1}H NMR (400 MHz, $CDCl_3$): δ 7.43 (d, *J* = 8.8 Hz, 2H), 7.02 (d, *J* = 8.8 Hz, 2H), 6.86 (d, *J* = 5.2 Hz, 2H), 6.41 (d, *J* = 5.2 Hz, 2H), 3.89 (s, 3H). ^{13}C NMR (100 MHz, $CDCl_3$): δ 162.5, 144.1, 133.8, 132.6, 131.8, 124.8, 118.8, 114.5, 114.4, 55.8.

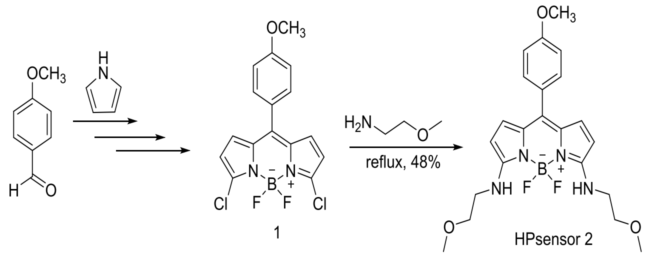

HPsensor 2: The mixture of compound 1 (40 mg, 0.11 mmol) in 2-methoxyethylamine (10 mL) was refluxed overnight under nitrogen atmosphere, and concentrated under reduced pressure. The residues were purified by column chromatography using hexanes/CH_2Cl_2/EtOAc (3:2:1, v/v) to yield 2 as oil (23 mg, 48%). ^{1}H NMR (400 MHz, $CDCl_3$): δ 7.36 (d, *J* = 8.4 Hz, 2H), 6.92 (d, *J* = 8.4 Hz, 2H), 6.54 (d, *J* = 4.4 Hz, 2H), 5.72 (d, *J* = 4.4 Hz, 2H), 3.84 (s, 3H), 3.57 (t, *J* = 5.6 Hz, 4H), 3.42 (t, *J* = 5.6 Hz, 4H), 3.39 (s, 6H). . ^{13}C NMR (100 MHz, $CDCl_3$): δ 160.2, 156.7, 131.8, 131.6, 129.3, 128.6, 127.6, 113.6, 101.2, 71.4, 59.2, 55.5, 44.5. IR (cm^{-1}): 3417, 3132, 2923, 2300, 1732, 1593, 1542, 1504, 1472, 1423, 1390, 1368, 1337, 1304, 1290, 1275, 1247, 1194, 1175, 1155, 1094, 1055, 1011, 969, 918, 886, 836, 781, 764, 750, 726, 702, 680. HRMS (ESI) calcd for $C_{22}H_{27}BF_2N_4O_3Na$ $[M+Na]^+$ 467.2042; found 467.2039.

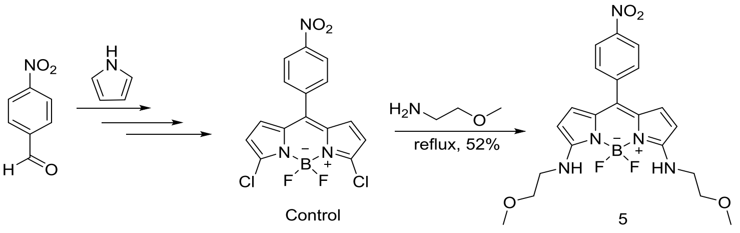

Control was prepared according to a reported procedure (*J. Org. Chem.*, **2008**, *73* (5), 1963–1970).

Compound 5 was prepared from control in 52% yields according to the method for HPsensor **2**. ^{1}H NMR (400 MHz, $CDCl_3$): δ 8.25 (d, *J* = 8.4 Hz, 2H), 7.59 (d, *J* = 8.4 Hz, 2H), 6.42 (d, *J* = 4.8 Hz, 2H), 5.77 (d, *J* = 4.8 Hz, 2H), 3.58 (t, *J* = 5.6 Hz, 4H), 3.44 (t, *J* =5.6 Hz, 4H), 3.39 (s, 6H). ^{13}C NMR (100 MHz, CDCl3): δ 157.1, 148.2, 142.1, 131.4, 128.7, 128.1, 127.9, 123.5, 102.3, 71.3, 59.3, 44.5. IR (cm^{-1}): 3410, 3316, 3106, 2919, 1590, 1546, 1475, 1427, 1344, 1098, 1013, 971, 848, 788, 764, 735, 707, 674. HRMS (ESI) calcd for $C_{21}H_{24}BF_2N_5O_4Na$ $[M+Na]^+$ 482.1787; found 482.1789.

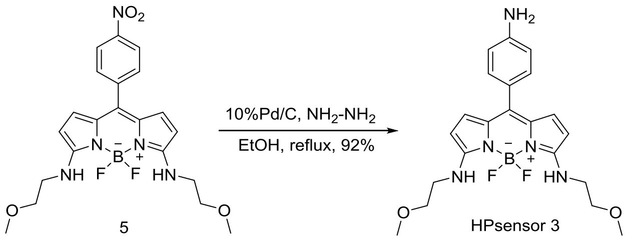

HPsensor 3 was prepared according to a reported procedure (*J. Org. Chem.*, **2008**, *73* (5), 1963–1970). ^{1}H NMR (400 MHz, $CDCl_3$): δ 7.20 (d, *J* = 8.4 Hz, 2H), 6.65 (d, *J* = 8.4 Hz, 2H), 6.58 (d, *J* = 4.4 Hz, 2H), 5.70 (d, *J* = 4.4 Hz, 2H), 3.56 (t, *J* = 5.6 Hz, 4H), 3.42-3.35 (m, 10H). ^{13}C NMR (100 MHz, $CDCl_3$): δ 156.5, 147.4, 132.3, 131.8, 129.1, 128.6, 125.1, 114.6, 100.9, 71.4, 59.2, 44.4. IR (cm^{-1}): 3413,3229, 3129, 2924, 1729, 1589, 1539, 1422, 1337, 1263, 1156, 1093, 1052, 1010, 965, 884, 835, 781, 764, 728, 679. HRMS (ESI) calcd for $C_{21}H_{26}BF_2N_5O_2Na$ $[M+Na]^+$ 430.2226; found 430.2227.

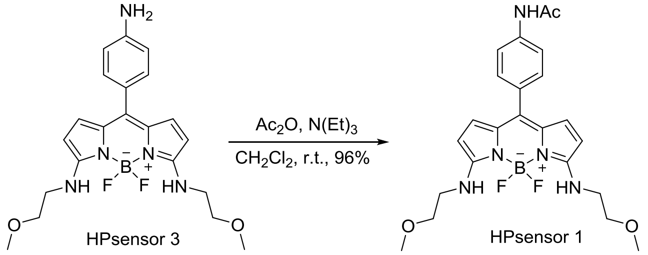

HPsensor 1: The solution of HPsensor 3 (20 mg, 0.047 mmol), acetic anhydride (0.2 mL), triethylamine (0.5 mL) and 4-DMAP(cat.) in CH_2Cl_2 (10 mL) was stirred under ice bath for 2 h, diluted by EtOAc, washed by H_2O, aqueous NH_4Cl, saturated aqueous $NaHCO_3$ and brine respectively, and dried (anhydrous Na_2SO_4), concentrated by rotated evaporation and purified by column chromatography using hexanes/CH_2Cl_2/EtOAc (3:2:1, v/v) to yield HPsensor 1 as oil (21 mg, 96%). ^{1}H NMR (400 MHz, $CDCl_3$): δ 7.64 (br, 1H), 7.50 (d, *J* = 8.4 hz, 2H), 7.32 (d, *J* = 8.4 Hz, 2H), 6.48 (d, *J* = 4.4 Hz, 2H), 5.70 (d-br, *J* =4.8 Hz, 4H), 3.56 (t, *J* = 5.2 Hz, 4H), 3.37-3.30 (m, 10H), 2.16 (s, 3H). ^{13}C NMR (100 MHz, $CDCl_3$): δ 168.9, 156.7, 138.7, 131.2, 131.0, 129.0, 128.6, 119.4, 101.4, 71.3, 59.2, 44.4, 29.5, 24.7. IR (cm^{-1}): 3421, 3308, 3180, 3111, 2925, 2893, 1731, 1665, 1594, 1545, 1474, 1425, 1339, 1260, 1086, 1055, 1009, 894, 846, 781, 762, 696. HRMS (ESI) calcd for $C_{23}H_{28}BF_2N_5O_3Na$ $[M+Na]^+$ 494.2151; found 494.2148.

Quantum yields of dyes in various solvents:

Quantum yield measurements were conducted in accordance with previously published protocol from Zhu et al 2012.[2] Quantum yields of dyes were calculated from absorption and emission measurements of dyes in dichloromethane, ethanol and water corrected for quantum yield of the dye standard at test wavelengths. Quantum yields of dyes were calculated using the following equation where st = standard; x = test dye; Grad – gradient of fitted slope; Q = quantum yield and **η** = refractive index of test solvent.

$$Q_x = Q_{st}(\frac{Grad_x}{Grad_{st}})(\frac{\eta_x^2}{\eta_{st}^2}) \qquad \text{(equation 1)}$$

Supplementary Table 1. Absorption and emission peak maxima of control dye, HPsensors 1, 2 and 3 with corresponding fluorescence quantum yield and the extinction coefficient in ethanol.

Dye	Solvent	Absorption peak (nm)	Emission peak (nm)	Fluorescence Quantum Yield (%)	Extinction coefficient (ethanol)
Control	Ethanol	517	540	7.99	14880 $M^{-1}cm^{-1}$ (at 517 nm)
	Water	518	540	0.15	
	CH_2Cl_2	521	545	5.58	
HPsensor **1**	Ethanol	565	585	23.99	50990 $M^{-1}cm^{-1}$ (at 565 nm)
	Water	564	584	6.77	
	CH_2Cl_2	569	587	19.92	
HPsensor **2**	Ethanol	564	581	45.03	31930 $M^{-1}cm^{-1}$ (at 564 nm)
	Water	563	580	1.27	
	CH_2Cl_2	567	584	42.21	
HPsensor **3**	Ethanol	562	577	36.17	53920 $M^{-1}cm^{-1}$ (at 562 nm)
	Water	561	579	0.25	
	CH_2Cl_2	566	582	35.39	

Response of dyes to change in solvent polarity.

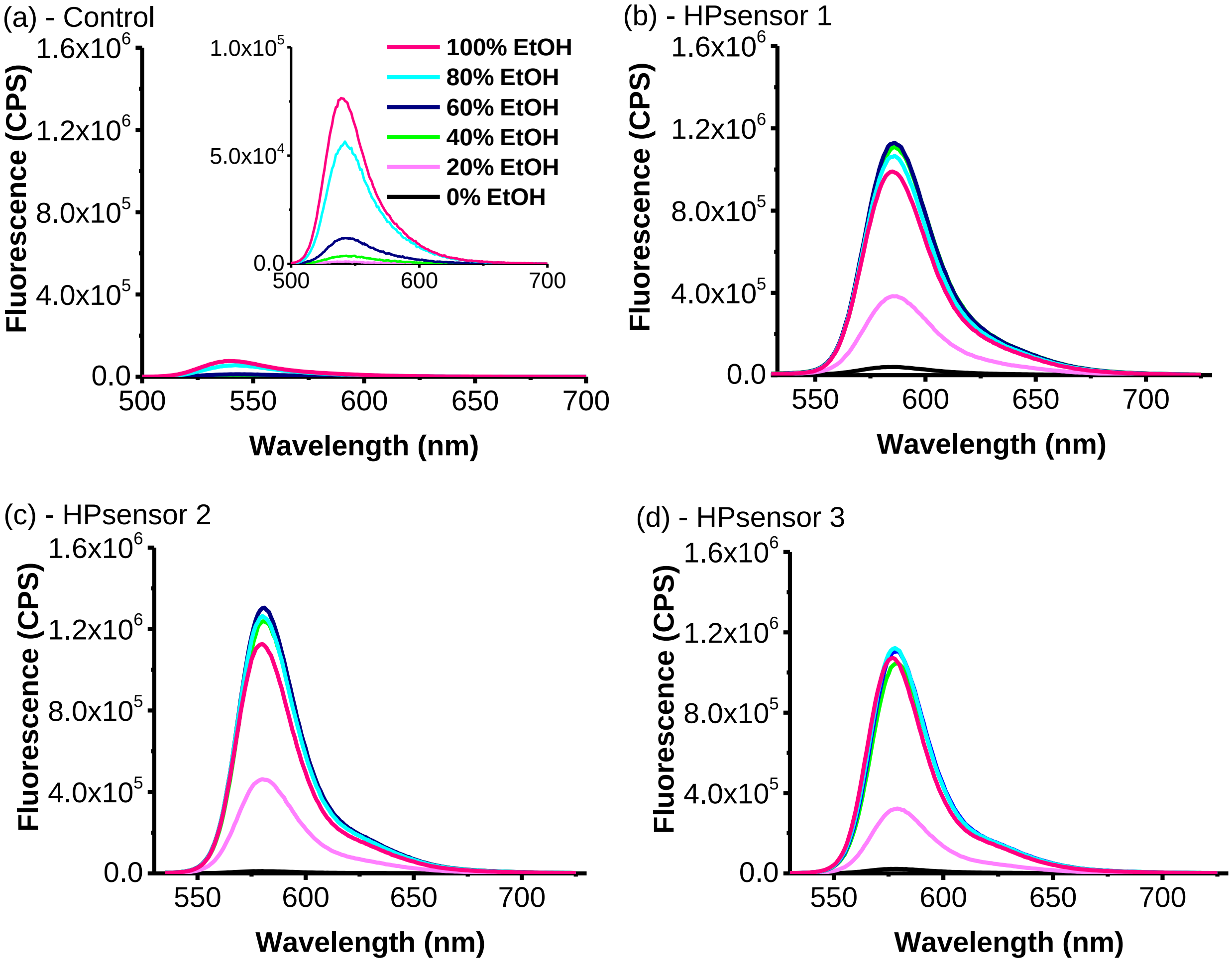

Supplementary Figure 1. Fluorescence spectra of 2 µM of control dye (a) and HPsensors 1 (b), 2 (c), and 3 (d) in ethanol-water mixture. HPsensors 1, 2, and 3 show maximum fluorescence at 60% ethanol whereas the control dye shows increase in fluorescence proportional to decrease in polarity. The emission spectra were collected after excitation at 520 nm for HPsensors 1 and 3, at 528 nm for HPsensor 2, and at 475 nm for the control dye.

Response of dyes to change in solvent polarity. The absorption and emission spectra of dyes were measured in solvents with different polarity (water, ethanol and dichloromethane). These measurements showed that all dyes were fluorescent with the exception of dye **5** that exhibited no fluorescence either in high or low polarity solvents. For the dyes that were fluorescent (HPsensors **1**, **2**, and **3**), the initial characterization showed a small red shift (2 to 5 nm) in absorbance and emission maxima with decreasing polarity (see Supplementary Figs. 2, 3, 4, and 5) which was similar to the control dye with the strong electron withdrawing substitution. To further investigate how polarity impacted the fluorescence spectra of each of these dyes, we measured the fluorescence in ethanol-water mixture with increasing concentration of ethanol (20% increments ranging from 0 to 100% ethanol) (see Supplementary Fig. 1). The results show that HPsensors **1**, **2**, and **3** responded similarly to the change in solvent conditions with maximum fluorescence in 60% ethanol. The exception was the control dye that showed a linear increase in fluorescence with increasing ethanol concentration (from 0% to 100% ethanol) (see Supplementary Fig. 1).

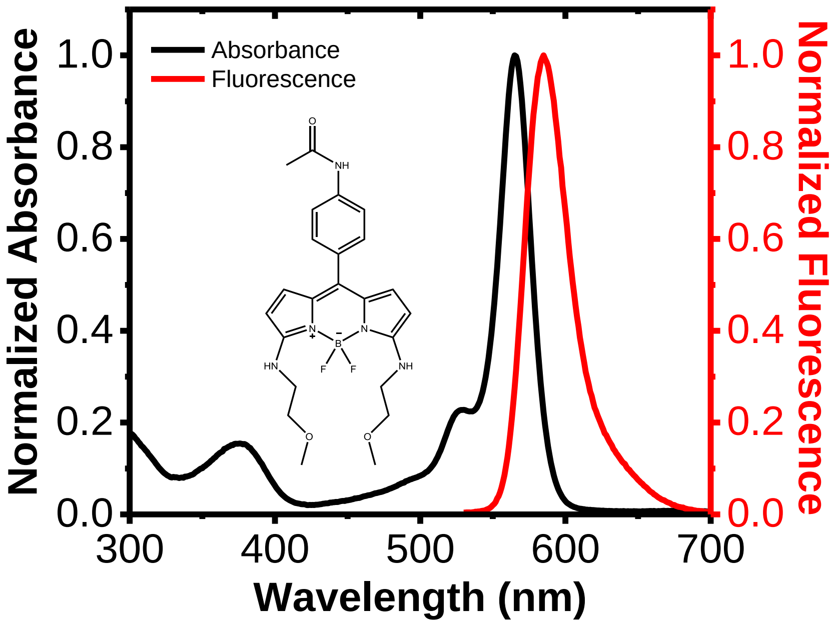

Supplementary Figure 2a Normalized absorption and emission spectra for HPsensor **1** in ethanol. Ex λ = 520 nm.

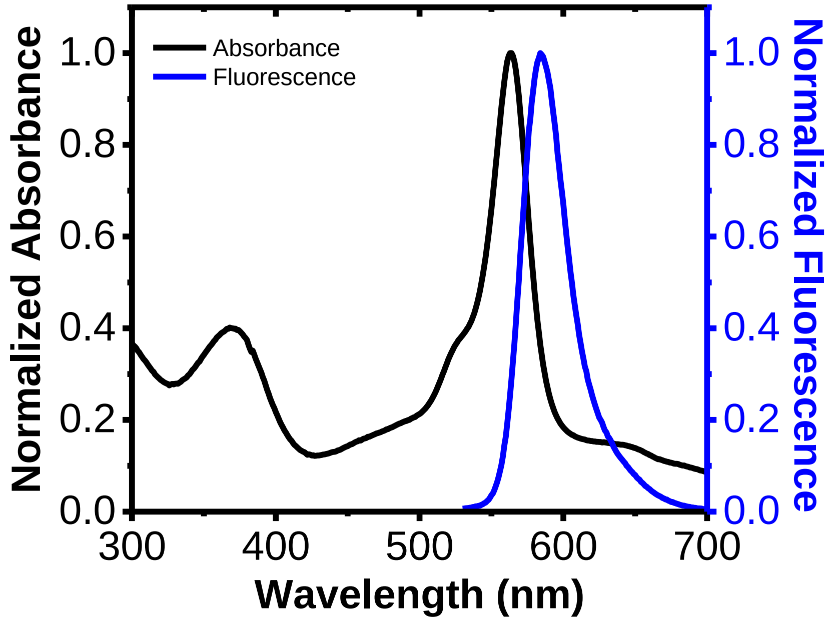

Supplementary Figure 2b Normalized absorption and emission spectra for HPsensor **1** in H_2O. Ex λ = 520 nm.

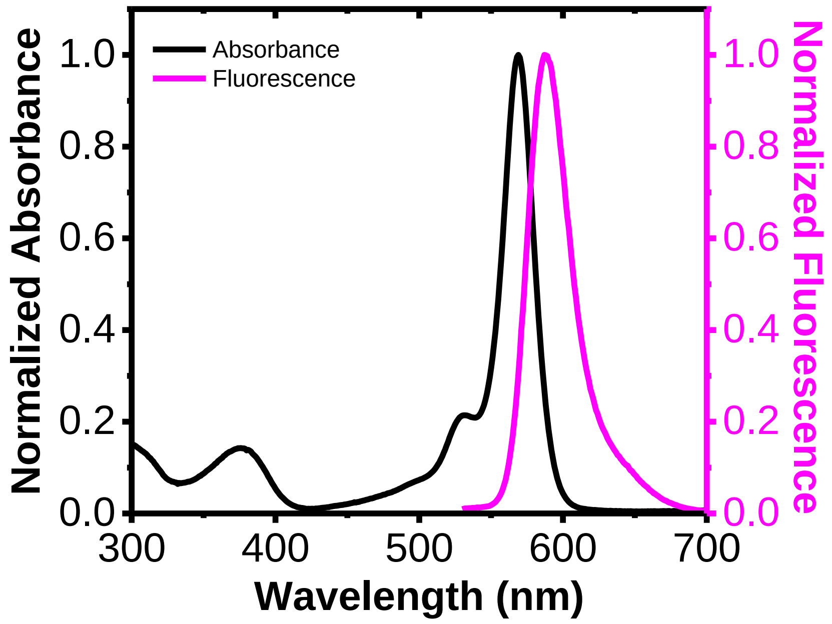

Supplementary Figure 2c Normalized absorption and emission spectra for HPsensor **1** in CH_2Cl_2. Ex λ = 520 nm.

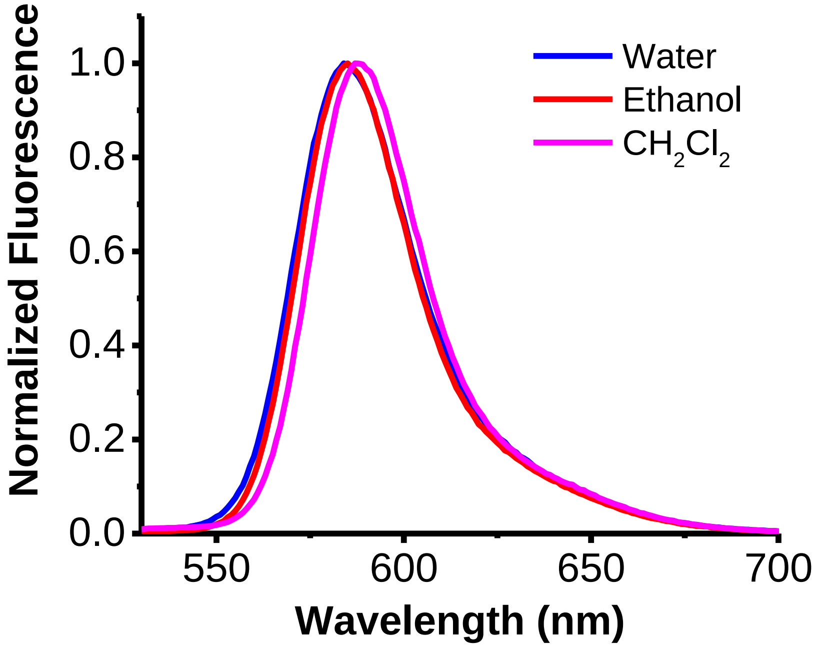

Supplementary Figure 2d Normalized emission spectra for HPsensor **1** in ethanol, H_2O, and CH_2Cl_2. Ex λ = 520 nm.

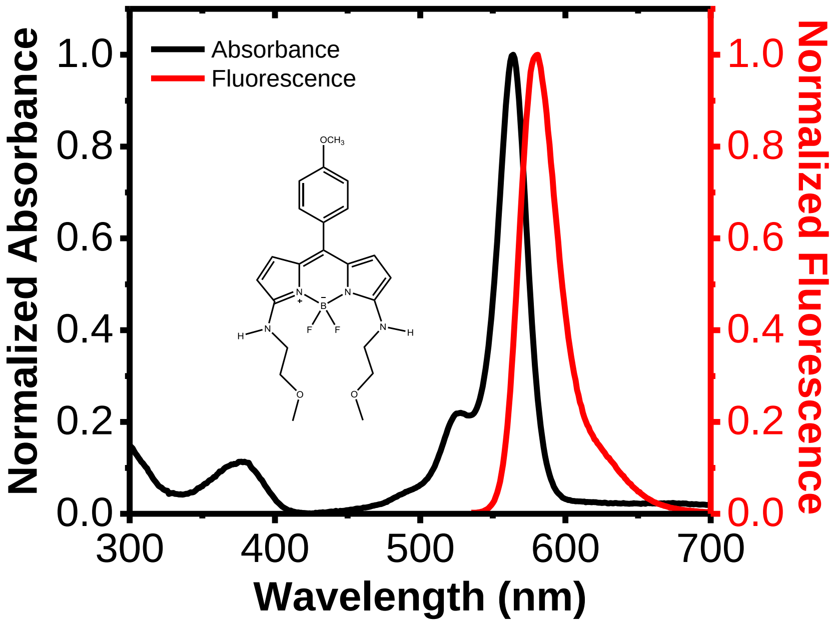

Supplementary Figure 3a Normalized absorption and emission spectra for HPsensor **2** in ethanol. Ex λ = 528 nm.

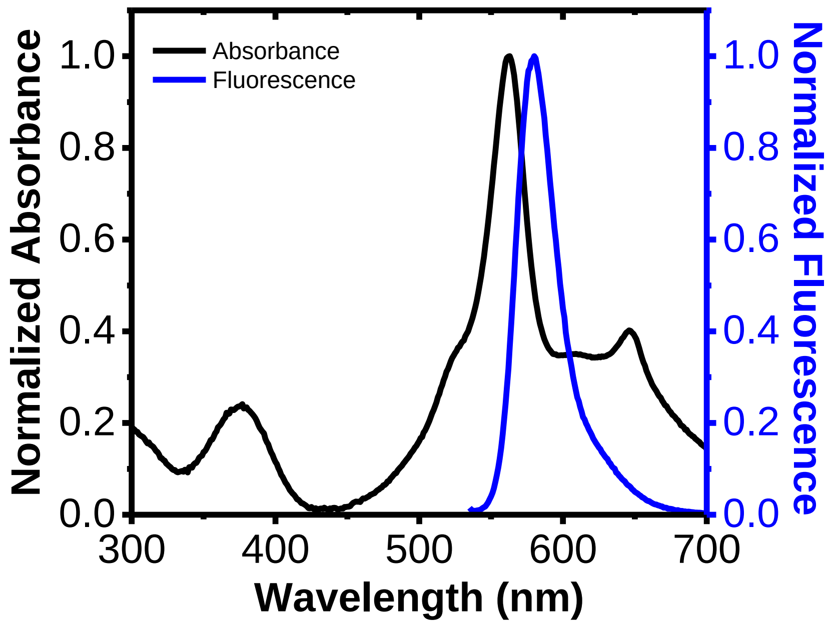

Supplementary Figure 3b Normalized absorption and emission spectra for HPsensor **2** in H_2O. Ex λ = 528 nm.

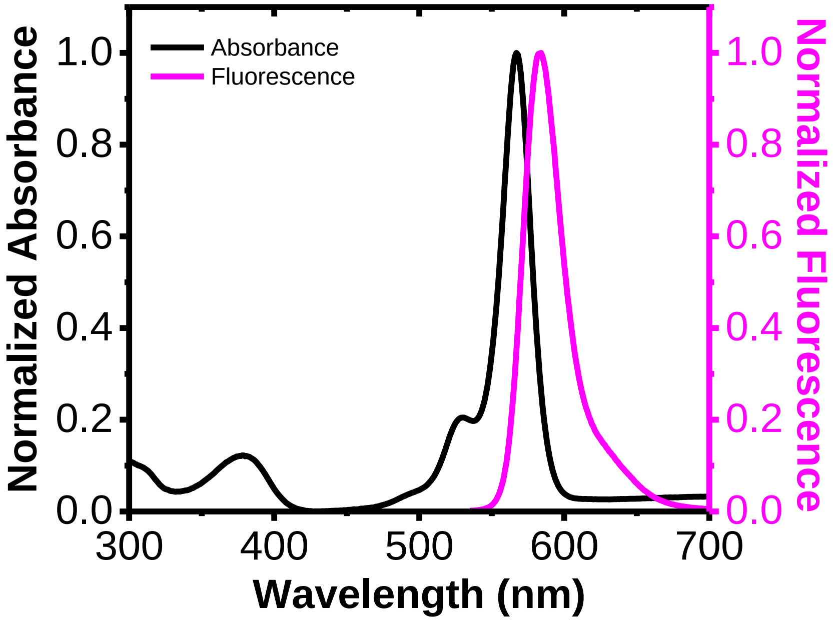

Supplementary Figure 3c Normalized absorption and emission spectra for HPsensor **2** in CH_2Cl_2. Ex λ = 528 nm.

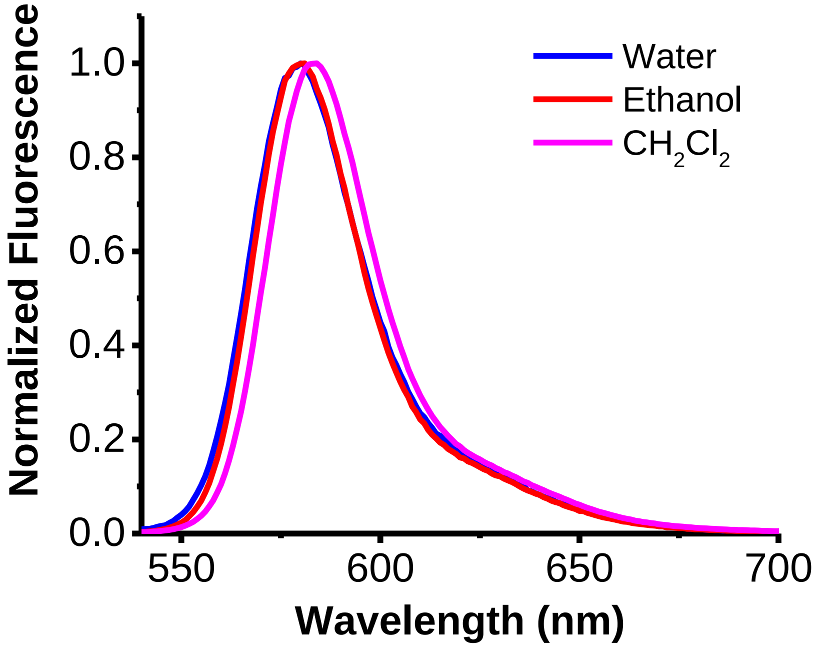

Supplementary Figure 3d Normalized emission spectra for HPsensor **2** in ethanol, H_2O, and CH_2Cl_2. Ex λ = 528 nm.

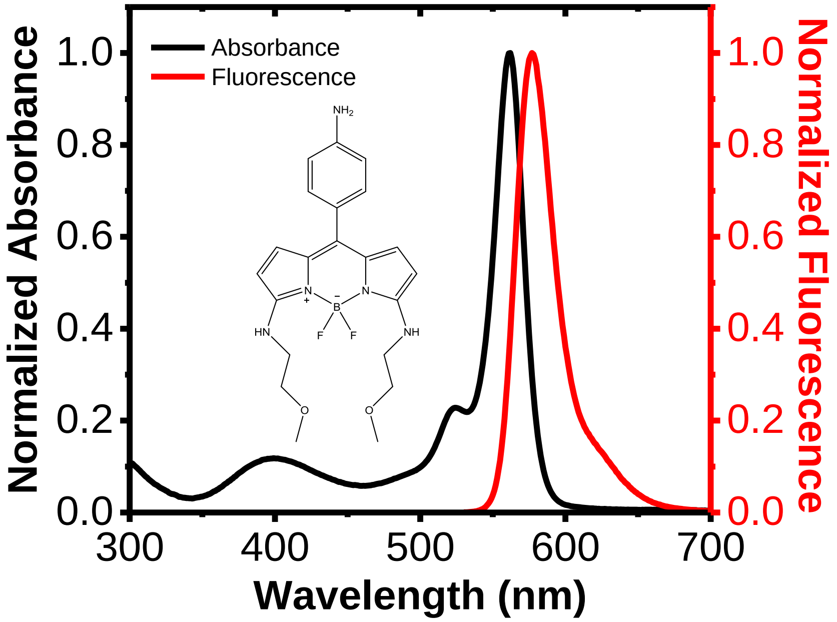

Supplementary Figure 4a Normalized absorption and emission spectra for HPsensor **3** in ethanol. Ex λ = 520 nm.

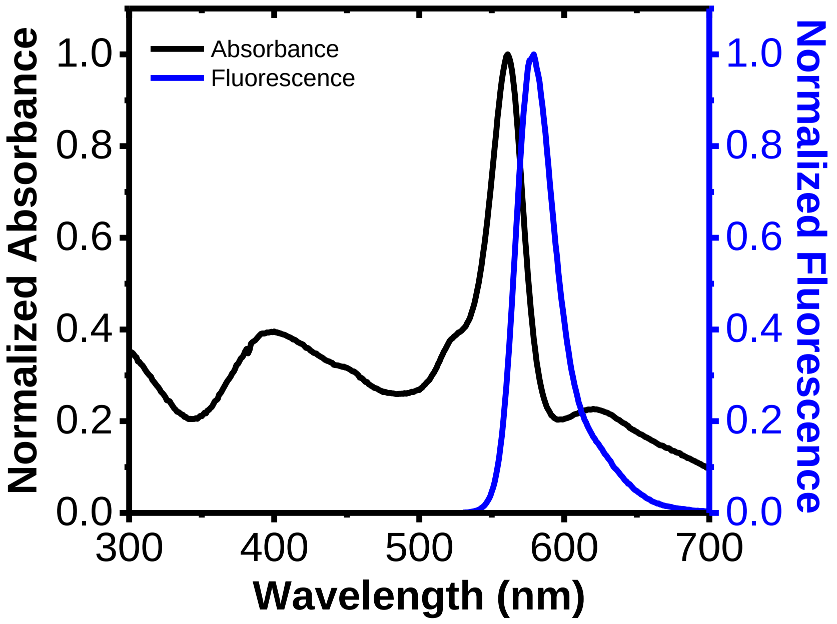

Supplementary Figure 4b Normalized absorption and emission spectra for HPsensor **3** in H_2O. Ex λ = 520 nm.

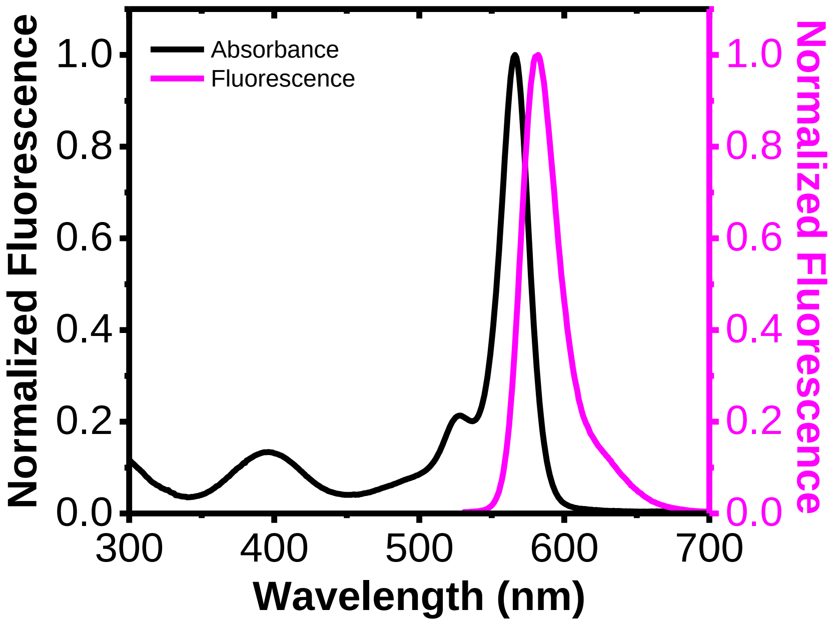

Supplementary Figure 4c Normalized absorption and emission spectra for HPsensor **3** in CH_2Cl_2. Ex λ = 520 nm.

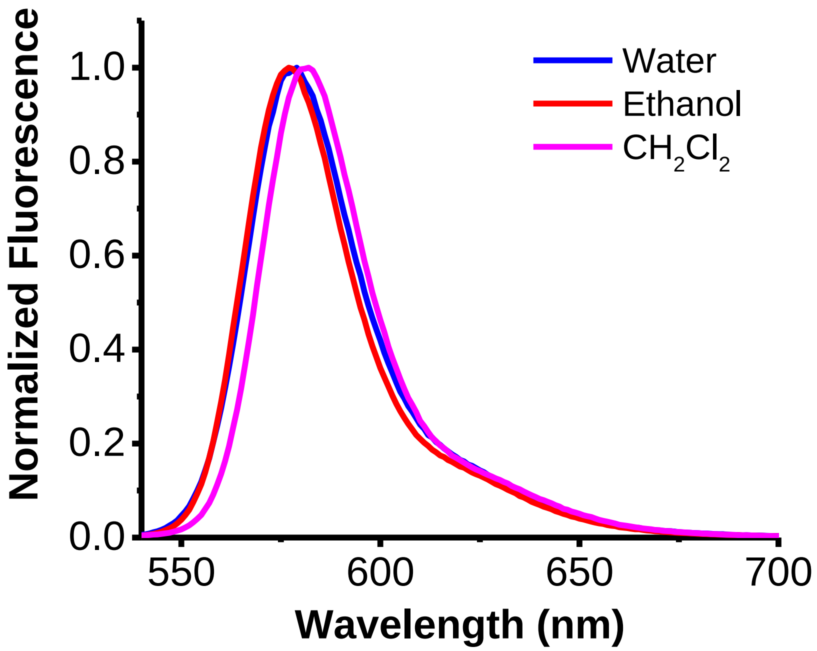

Supplementary Figure 4d Normalized emission spectra for HPsensor **3** in ethanol, H_2O, and CH_2Cl_2. Ex λ = 520 nm.

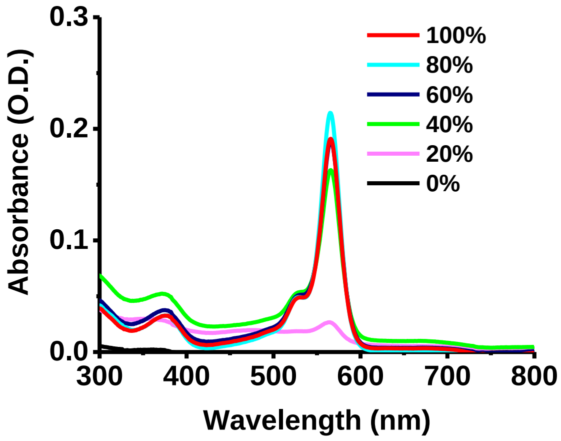

Supplementary Figure 5a Absorption spectra of 2 µM of HPsensor **1** in ethanol-water mixture from 0% to 100% ethanol at 20% intervals.

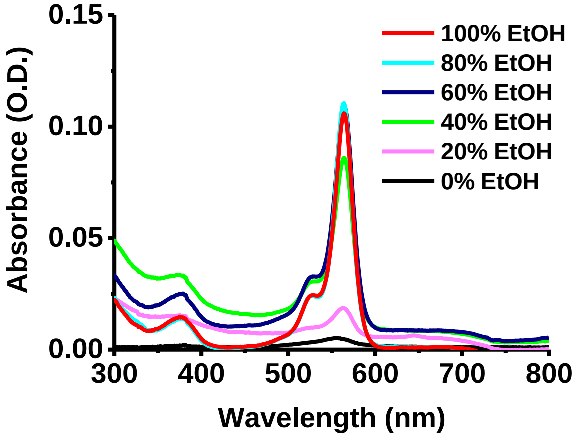

Supplementary Figure 5b Absorption spectra of 2 µM of HPsensor 2 in ethanol-water mixture from 0% to 100% ethanol at 20% intervals.

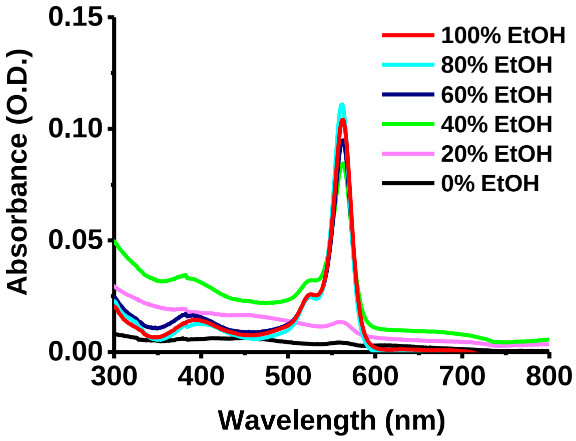

Supplementary Figure 5c Absorption spectra of 2 µM of HPsensor **3** in ethanol-water mixture from 0% to 100% ethanol at 20% intervals.

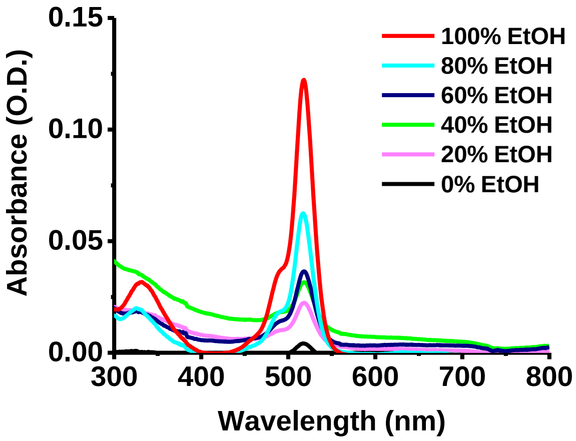

Supplementary Figure 5d Absorption spectra of 2 µM of control dye in ethanol-water mixture from 0% to 100% ethanol at 20% intervals.

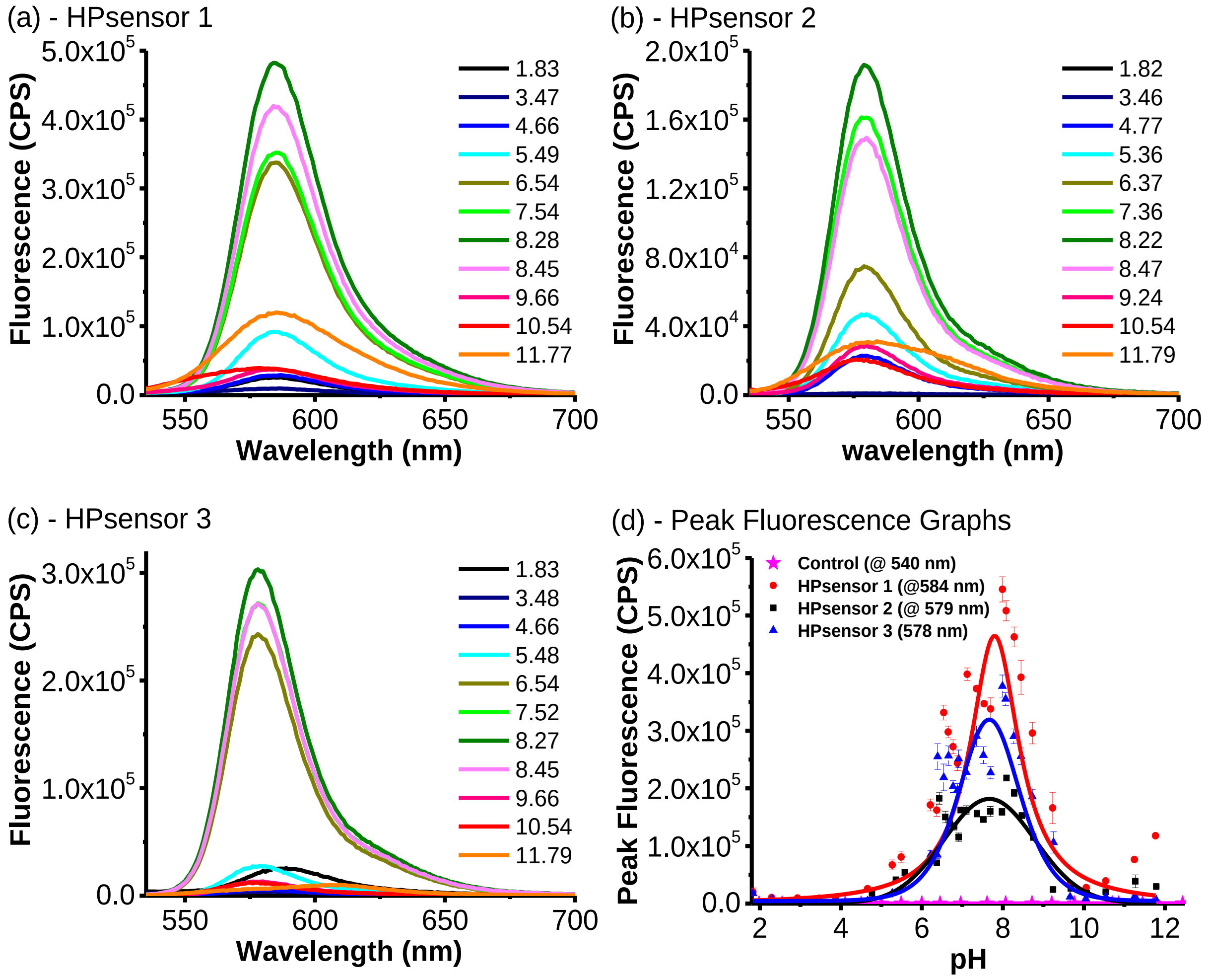

Supplementary Figure 6. Fluorescence spectra of control and HPsensors **1**, **2** and **3** show sensitivity to change in pH. Select plots of HPsensors **1, 2** and **3** with increasing pH (a – c). The mean peak intensity plotted at the indicated wavelength vs pH for all HPsensors and control dye (d). Dyes were incubated at 2 µM concentration at room temperature in Carmody buffer with pH ranging from ~ 2 to 12 before acquiring the emission spectra. The emission spectra were collected after excitation at 520 nm for HPsensors **1** and **3**, at 528 nm for HPsensor **2** and at 475 nm for the control dye.

Effect of pH on fluorescence of dyes. The dyes were tested for the effect of pH on fluorescence intensity using Carmody buffer series in pH range from 2 to 12. The fluorescence spectra for 2 µM concentration of control and HPsensors were acquired at different pH values in triplicate and a mean peak intensity vs pH for each dye was plotted (Supplementary Fig. 6). Mean peak fluorescence intensity for the control dye was at 540 nm, while for HPsensors **1**, **2** and **3** it was at 584, 579 and 578 nm, respectively. While the HPsensors showed most sensitivity in the pH range from 6.5 to 9, the control dye did not show any pH sensitivity (Supplementary Fig. 6). When tested for pH stability all HPsensors (**1**, **2** and **3**) showed an increase in fluorescence as the pH increased from 3 to 8; when the pH was decreased from 8 to 3, a comparable decrease in fluorescence was observed (Supplementary Fig. 7).

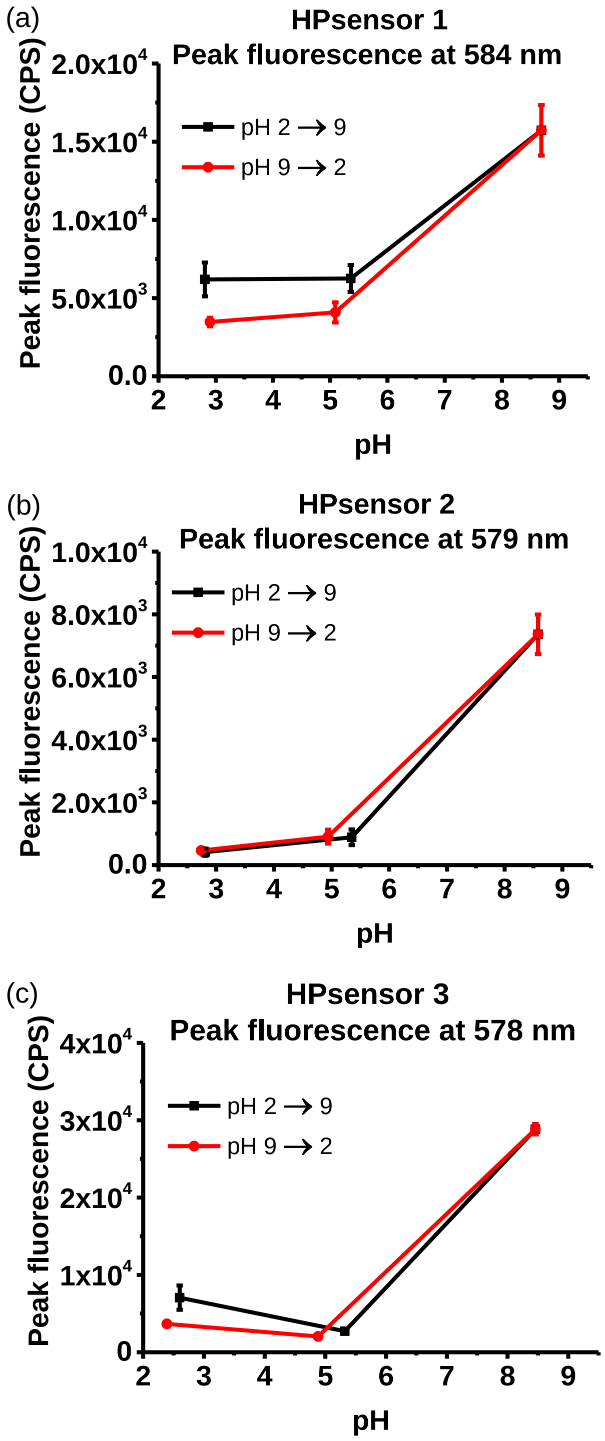

Supplementary Figure 7. Mean peak fluorescence of HPsensors with pH changes. Mean peak fluorescence of each HPsensor (at 2 µM) is plotted with increasing pH (pH ~2.0 to pH ~ 9.0) followed by decrease in pH (pH ~ 9.0 to pH ~ 2.0). All experiments were done in triplicate. Error bars indicate ± SD. The excitation and emission wavelength for each dye used is: HPsensor **1**, Ex 520 nm, Em 584 nm; HPsensor **2**, Ex 528 nm, Em 579 nm; HPsensor **3**, Ex 520 nm, Em 578 nm.

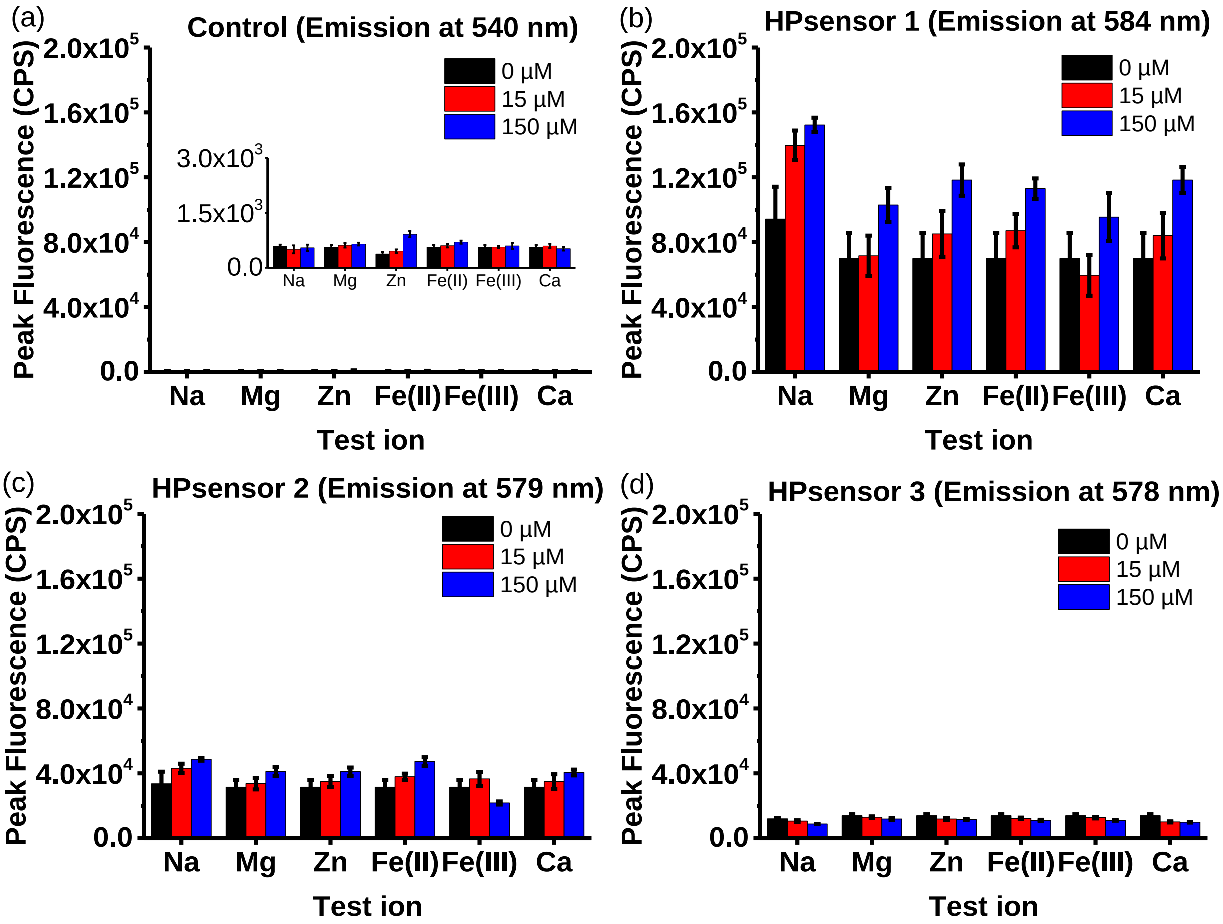

Supplementary Figure 8. Mean peak fluorescence of control and HPsensors with test ions (Na^{+}, Mg^{2+}, Fe^{2+}, Fe^{3+}, Ca^{2+}, Zn^{2+}) in water. Mean peak fluorescence of each HPsensor (at 2 µM) is plotted in the presence of increasing concentration of ions (0 to 150 µM). All experiments were done in triplicate. Error bars indicate ± SD. The excitation and emission wavelength for each dye used is: Control dye, Ex 475 nm, Em 540 nm; HPsensor **1**, Ex 520 nm, Em 584 nm; HPsensor **2**, Ex 528 nm, Em 579 nm; HPsensor **3**, Ex 520 nm, Em 578 nm.

Effect of ions on dye fluorescence**.** Dyes were tested for their selectivity to protein hydrophobicity over different ions (Na+, Mg2+, Zn2+, Fe2+, Fe3+, Ca2+) commonly found in buffers or as impurities in solutions. The investigation of the dyes with ions showed that the HPsensors are insensitive to the metal ions because their fluorescence was not significantly enhanced or quenched in the presence of ions even up to physiologically relevant concentrations of 150 µM[3,4].

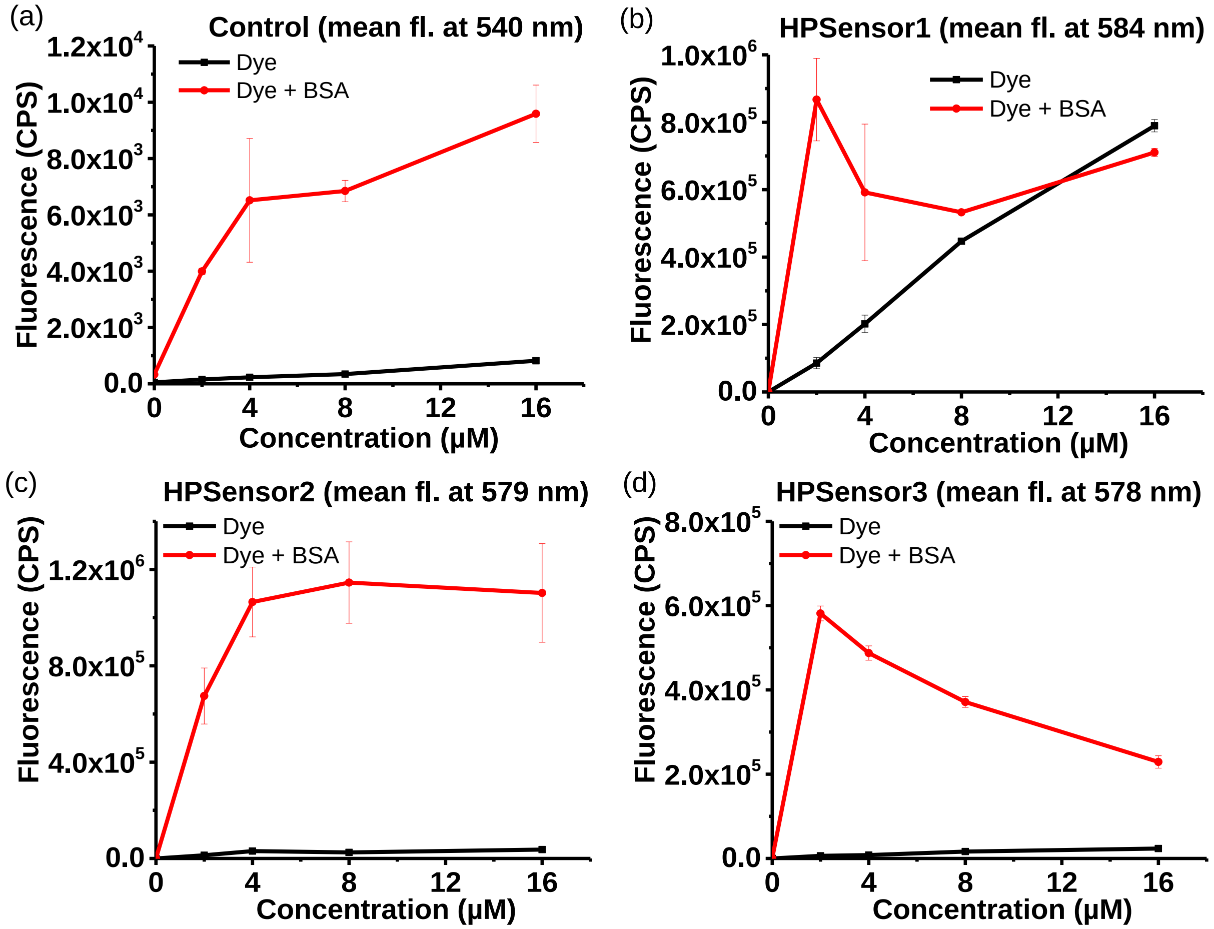

Supplementary Figure 9. Mean peak fluorescence of increasing concentration of control and HPsensors in presence and absence of BSA in water (pH adjusted to 8.0). Mean peak fluorescence of each dye in the absence and presence of BSA (2 μM) is plotted with increasing concentration of control or HPsensor (0 – 16 μM). All experiments were done in triplicate. Error bars indicate ± SD. The excitation and emission wavelength for each dye used is: Control dye, Ex 475 nm, Em 540 nm; HPsensor **1**, Ex 520 nm, Em 584 nm; HPsensor **2**, Ex 528 nm, Em 579 nm; HPsensor **3**, Ex 520 nm, Em 578 nm.

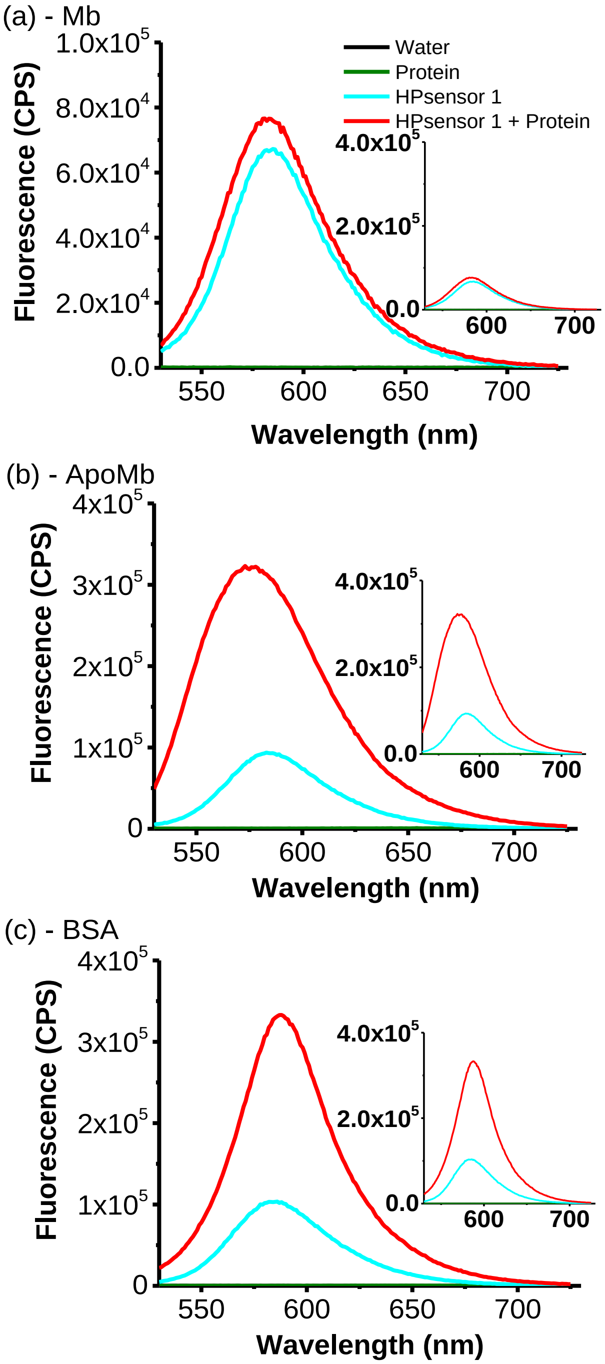

Supplementary Figure 10. Fluorescence emission spectra for HPsensor **1** incubated with (a) myoglobin, (b) apomyoglobin, and (c) BSA. Insets are shown on the same scale for ease of comparison between relative protein signals. Dye was incubated with protein at 1:1 ratio (2 µM each) for 1 hour at 25 °C with appropriate controls before spectra were acquired. Excitation wavelength was 520 nm.

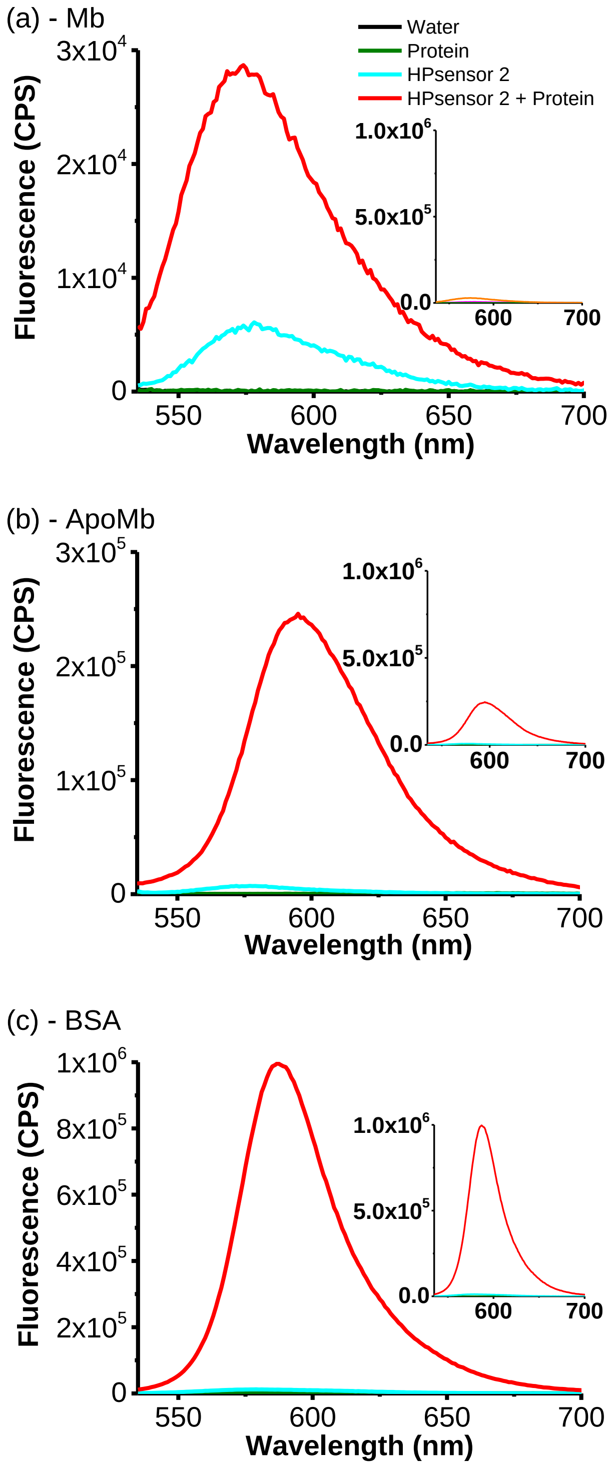

Supplementary Figure 11. Fluorescence emission spectra for HPsensor **2** incubated with (a) myoglobin, (b) apomyoglobin, and (c) BSA. Insets are shown on the same scale for easy comparison of relative protein signals. Dye was incubated with protein at 1:1 ratio (2 μM each) for 1 h at 25 °C with appropriate controls before spectra were acquired. The emission spectra for HPsensor **2** were collected after excitation at 528 nm.

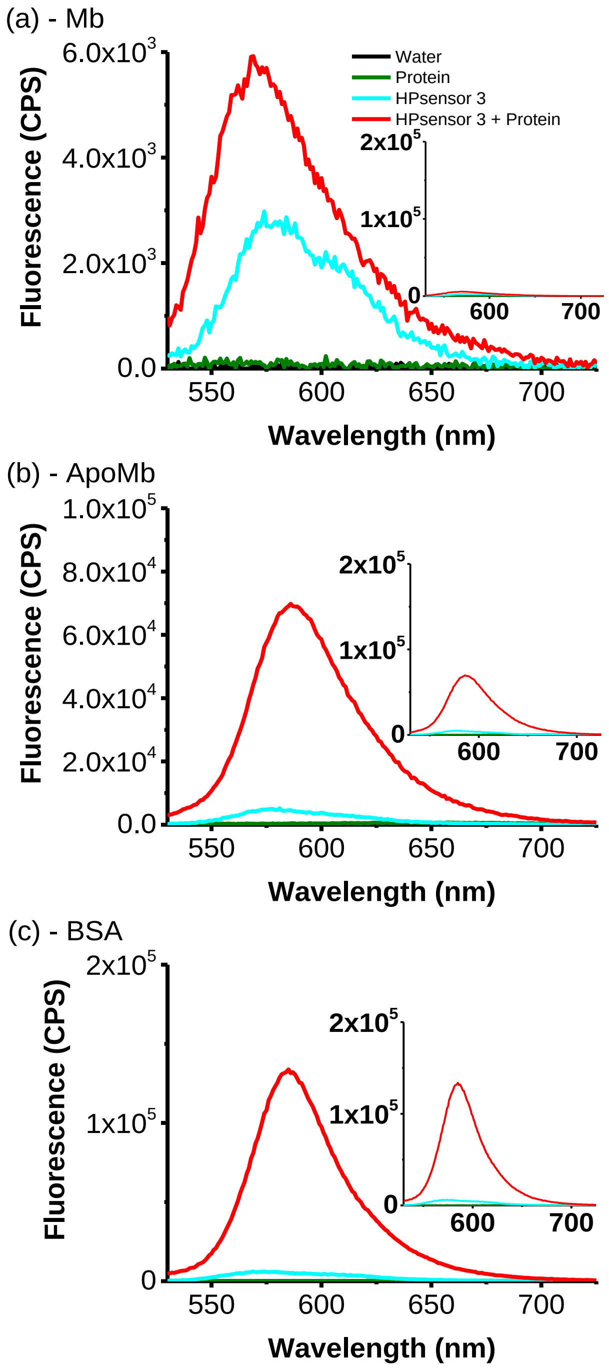

Supplementary Figure 12. Fluorescence emission spectra for HPsensor **3** incubated with (a) myoglobin, (b) apomyoglobin, and (c) BSA. Insets are shown on the same scale for ease of comparison between relative protein signals. Dye was incubated with protein at 1:1 ratio (2 µM each) for 1 hour at 25 °C with appropriate controls before spectra were acquired. Excitation wavelength was 520 nm.

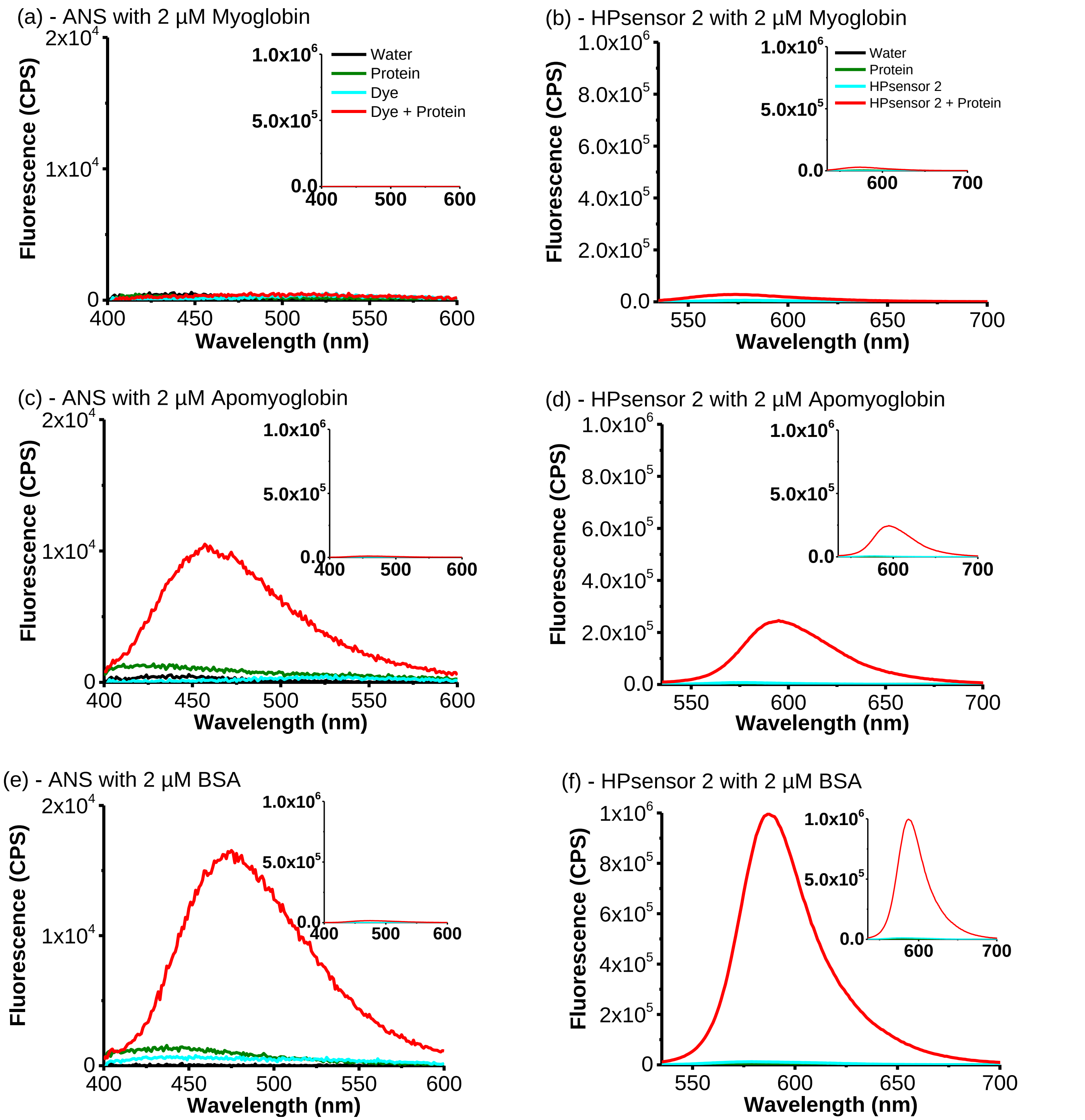

Supplementary Figure 13. Fluorescence emission spectra for ANS and HPsensor **2** incubated with (a - b) myoglobin, (c - d) apomyoglobin, and (e - f) BSA, respectively. Insets are shown on the same scale for ease of comparison of relative protein + dye signal.   Excitation wavelengths used are 350 nm for ANS and 528 nm for HPsensor **2**.

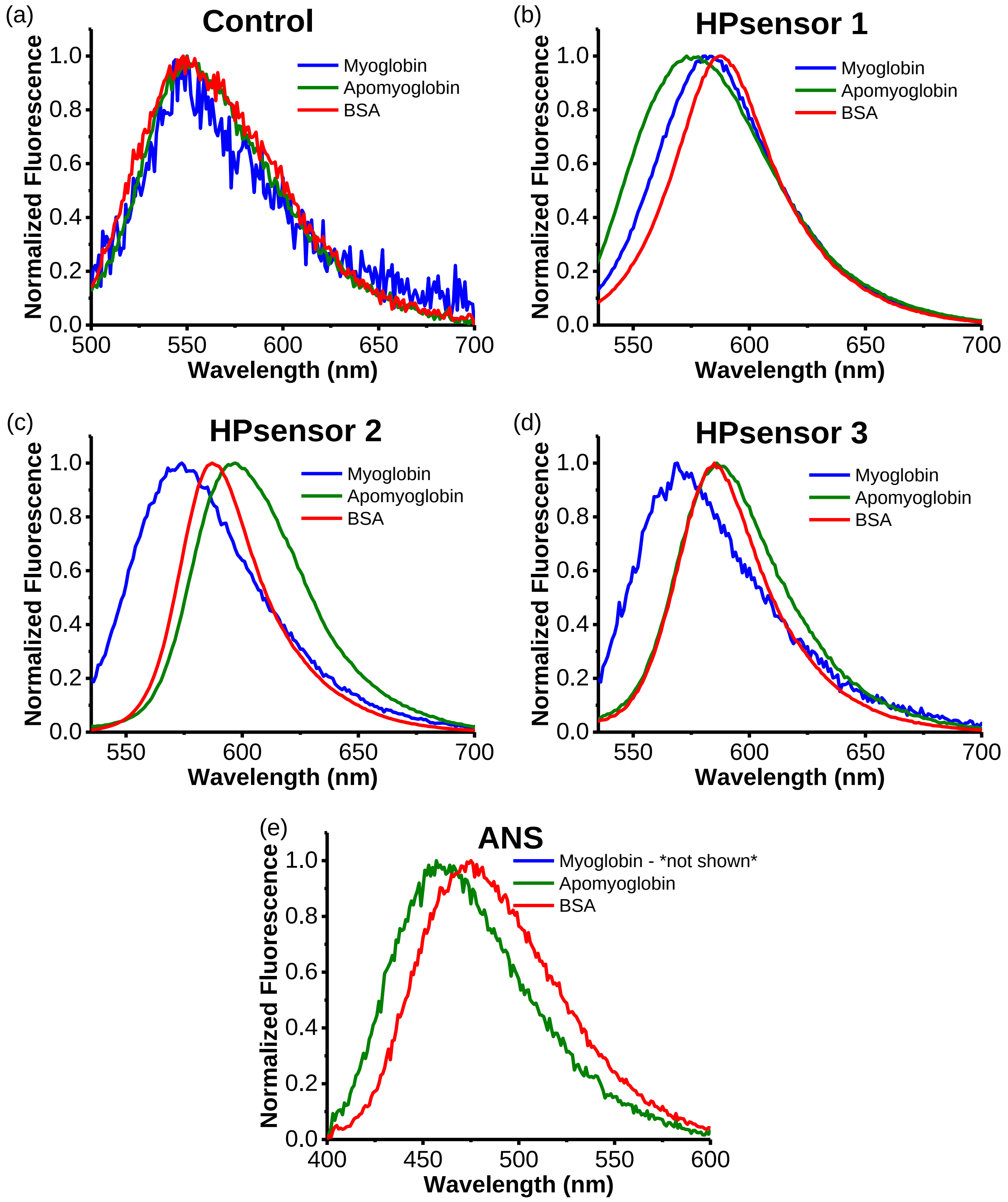

Supplementary Figure 14. Normalized emission spectra for control and HPsensors incubated with myoglobin (blue), apomyoglobin (green), and BSA (red). (a) Control dye, (b) HPsensor 1 dye, (c) HPsensor **2**, (d) HPsensor **3** and (e) ANS were all incubated with protein at 1:1 ratio (2 µM) for 1 hour at 25 °C with appropriate controls before emission spectra were acquired. For ANS with Mb, no significant fluorescence signal was measured, and as a result, the normalized plot was not included.

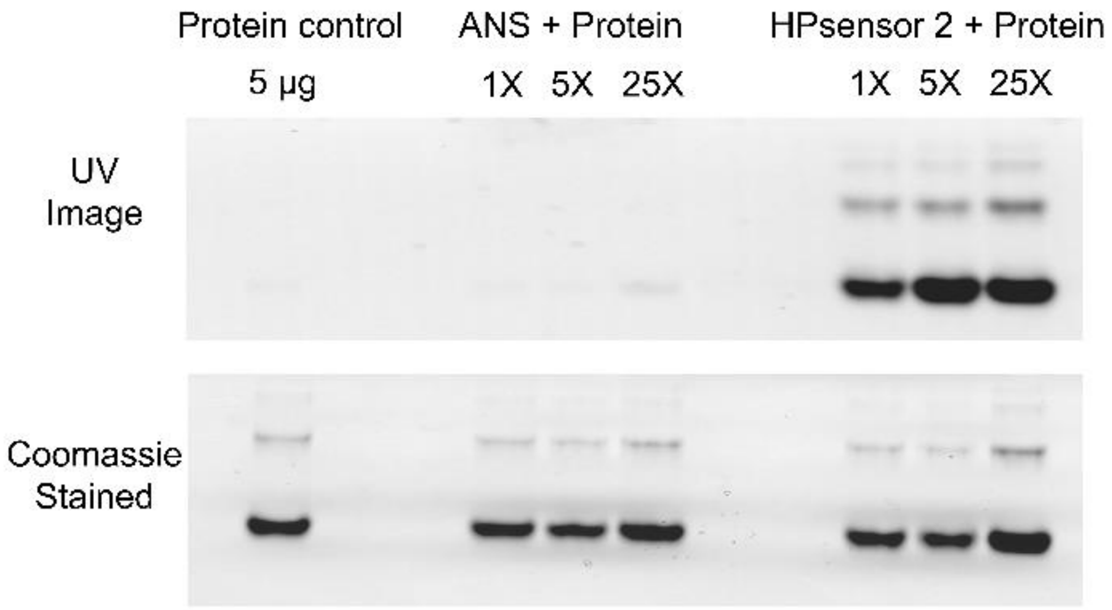

Supplementary Figure 15. Native PAGE of BSA (5 µg) with ANS and HPsensor **2**. 5 µg of BSA was incubated with dyes (ANS or HPsensor **2**) at 1X, 5X, and 25X concentration for 1 h at room temperature. BSA was then run on 10% Tris-HCl gel for 3 h at 80 V before exposure to UV light or Coomassie blue. Full length gel is included in supplementary figure 33. Brightness and contrast settings were adjusted for aesthetic purposes.

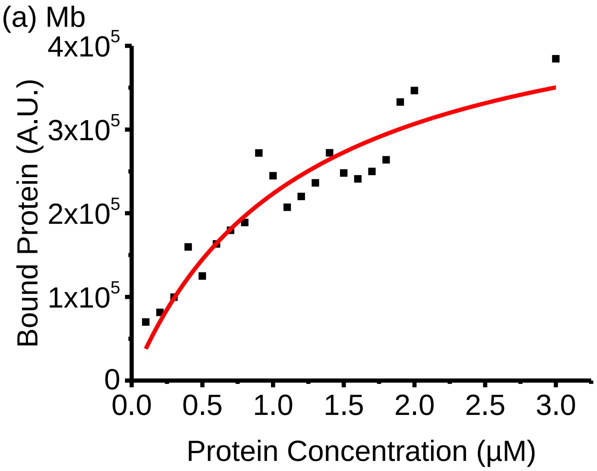

Model	MichaelisMenten		
Equation	y = Vmax * x / (Km + x)		
Reduced Chi-Sqr	9.13E+08		
Adj. R-Square	0.87047		
		Value	Standard Error
Bound Protein	Vmax	489495	58307.66
Bound Protein	Km	1.19197	0.30083

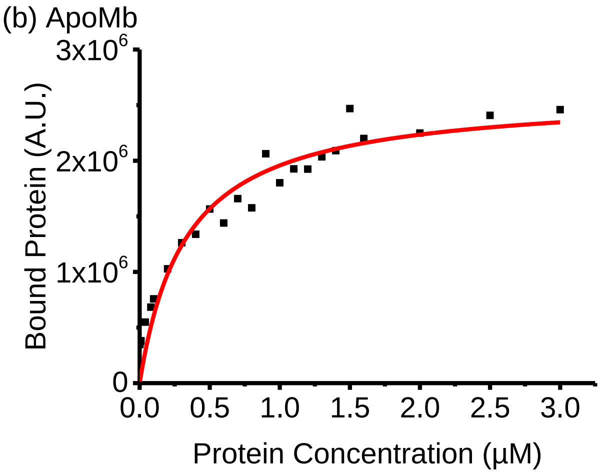

Model	MichaelisMenten		
Equation	y = Vmax * x / (Km + x)		
Reduced Chi-Sqr	3.93E+10		
Adj. R-Square	0.92582		
		Value	Standard Error
Bound Protein	Vmax	2.60E+06	137983.9
Bound Protein	Km	0.3305	0.06527

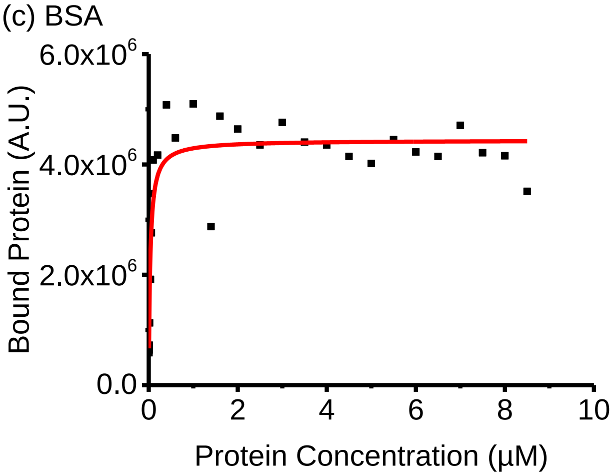

Model	MichaelisMenten		
Equation	y = Vmax * x / (Km + x)		
Reduced Chi-Sqr	2.76E+11		
Adj. R-Square	0.85572		
		Value	Standard Error
Bound Protein	Vmax	4.44E+06	126271
Bound Protein	Km	0.03395	0.00754

Supplementary Figure 16. **Binding affinity of test proteins (Mb, ApoMb, BSA).** Plot of Bound protein vs protein concentration for Mb (a), ApoMb (b) and BSA (c) with 0.5 µM HPsensor **2**. Plots show non-linear regression using the MichaelisMenten model. Fitting parameters are indicated in tabular form next to each plot.

(a) - Mb

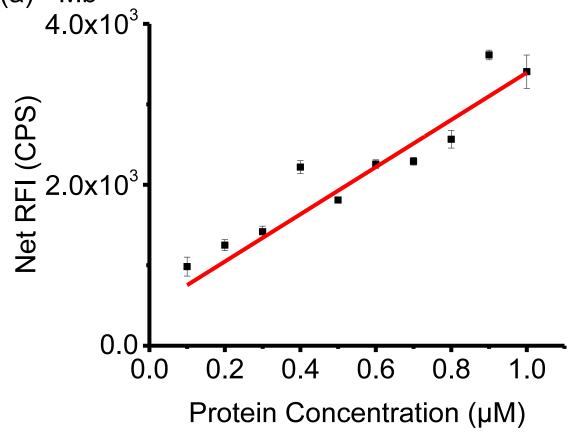

Equation	y = a + b*x		
Weight	Instrumental		
Residual Sum of Squares	190.9159		
Pearson's r	0.914497		
Adj. R-Square	0.815844		
		Value	Standard Error
Net RFI	Intercept	459.6181	258.7922
Net RFI	Slope	2934.333	458.9853

(b) - ApoMb

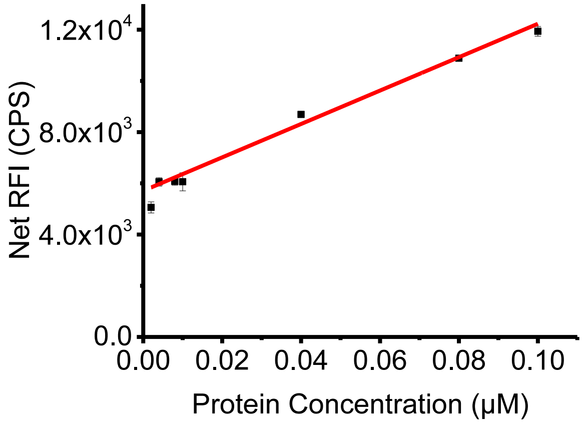

Equation	y = a + b*x		
Weight	Instrumental		
Residual Sum of Squares	32.61916		
Pearson's r	0.992156		
Adj. R-Square	0.981249		
		Value	Standard Error
Net RFI	Intercept	5708.557	230.1198
Net RFI	Slope	65212.15	3674.367

(c) - BSA

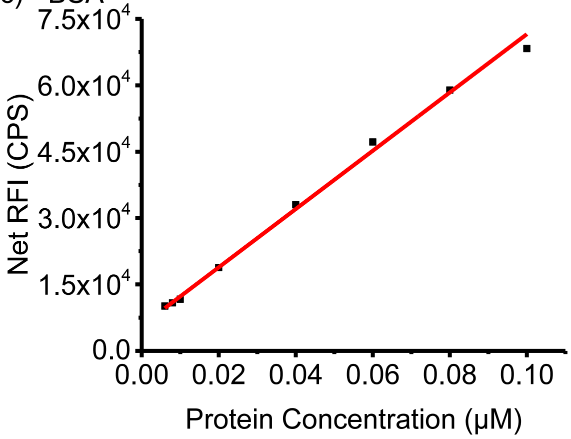

Equation	y = a + b*x		
Weight	Instrumental		
Residual Sum of Squares	249.0748		
Pearson's r	0.999324		
Adj. R-Square	0.998422		
		Value	Standard Error
Net RFI	Intercept	5701.874	505.8403
Net RFI	Slope	658608.5	9895.112

Supplementary Figure 17. **Surface hydrophobicity of test proteins (Mb, ApoMb, BSA).** Plot of Net relative fluorescence intensity (RFI) vs protein concentration with 0.5 μM HPsensor **2**. Plots show linear regression for Mb (a), ApoMb (b) and BSA (c) at increasing concentration plotted with the Net RFI (at 579 nm). Fitting parameters are indicated in tabular form next to each plot.

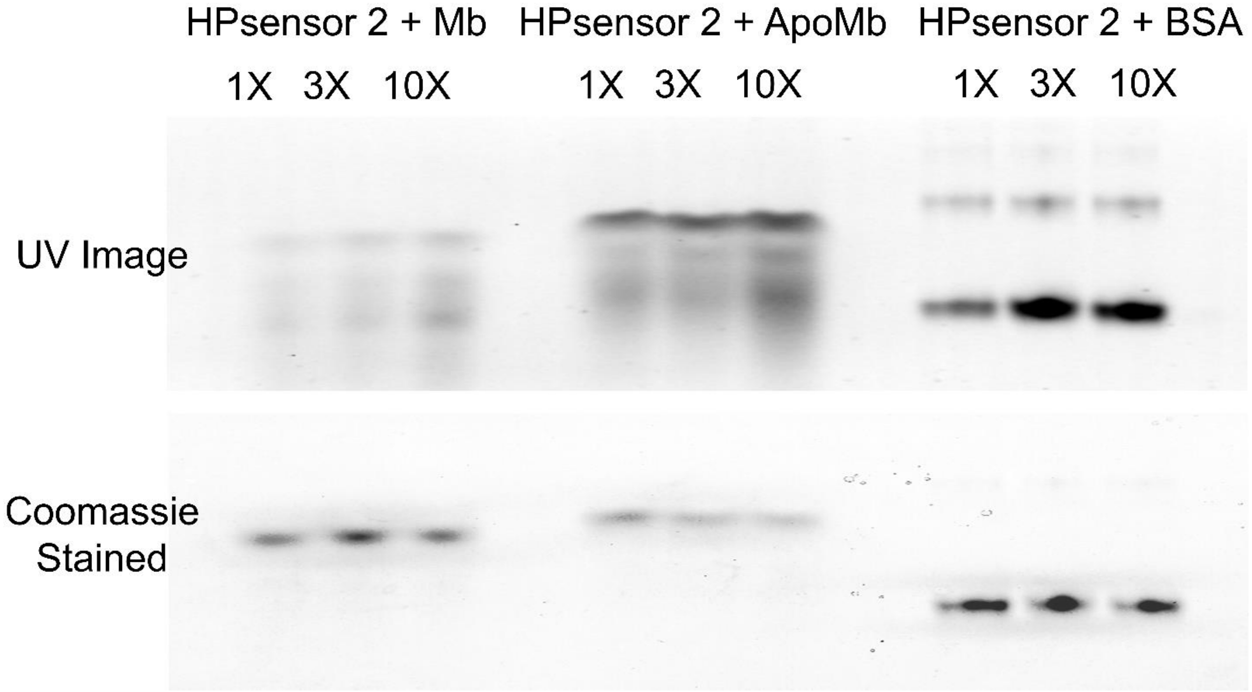

Supplementary Figure 18. Native PAGE of 2 µg of Proteins [Myoglobin (Mb), Apomyoglobin (ApoMb), BSA] with HPsensor **2**. 2 µg of each protein was incubated with HPsensor **2** at 1X, 3X, and 10X concentration for 1 h at room temperature. Proteins were then run on 10% Tris-HCl gel for 4 h at 80 V before exposure to UV light or Coomassie blue. Full length gel is included in supplementary figure 34. Brightness and contrast settings were adjusted for aesthetic purposes.

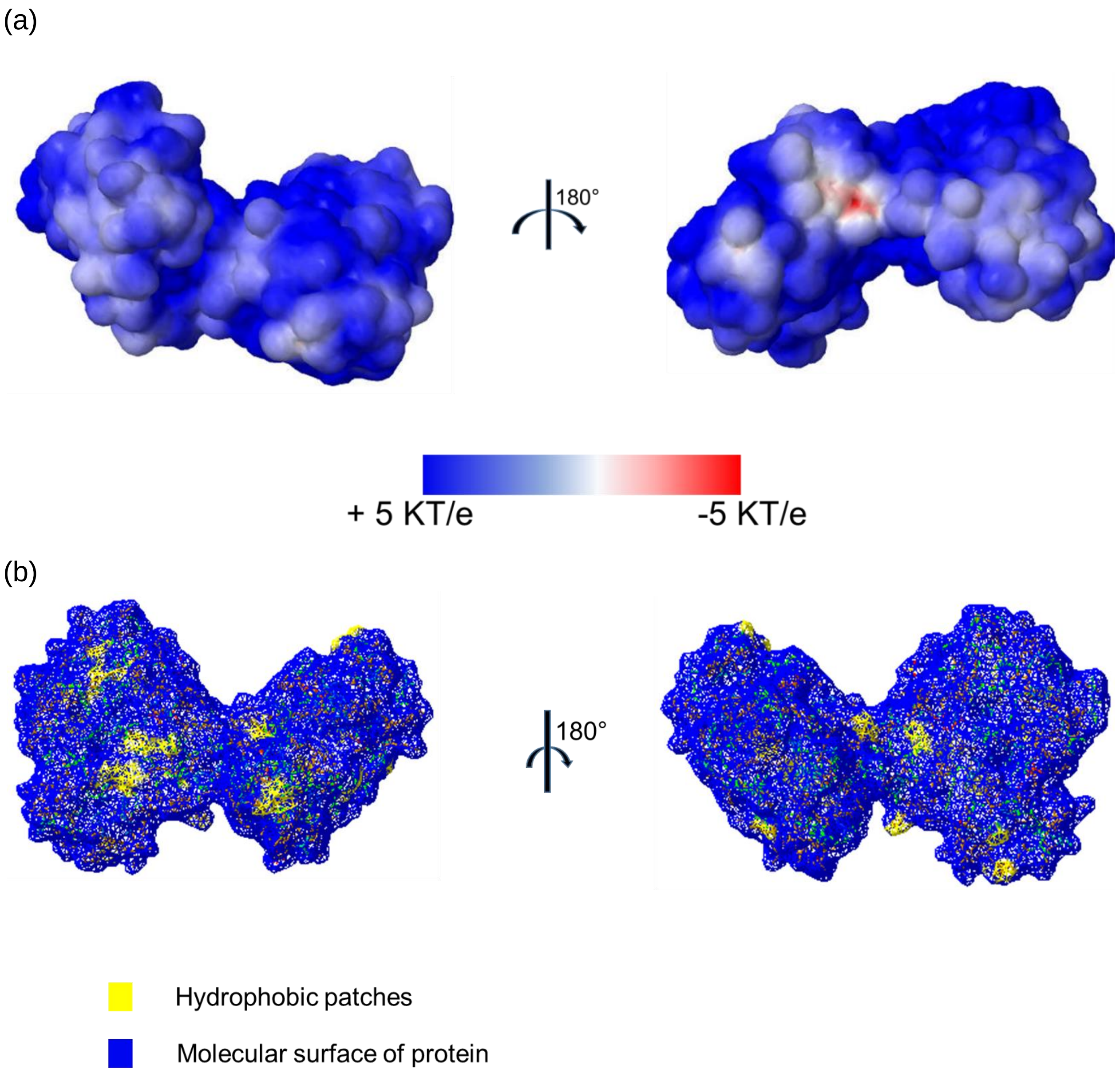

Supplementary Figure 19. **Electrostatic and Hydrophobic patch maps of Myoglobin (Mb: PDB ID 3RJ6).** Maps show (a) the electrostatic surface potentials of Mb visualized as isocontours at +5.0 kT/e (blue) and -5.0 kT/e (red) using APBS and (b) predicted hydrophobic patches (yellow) visualized against the molecular surface (blue) using SPDB software.

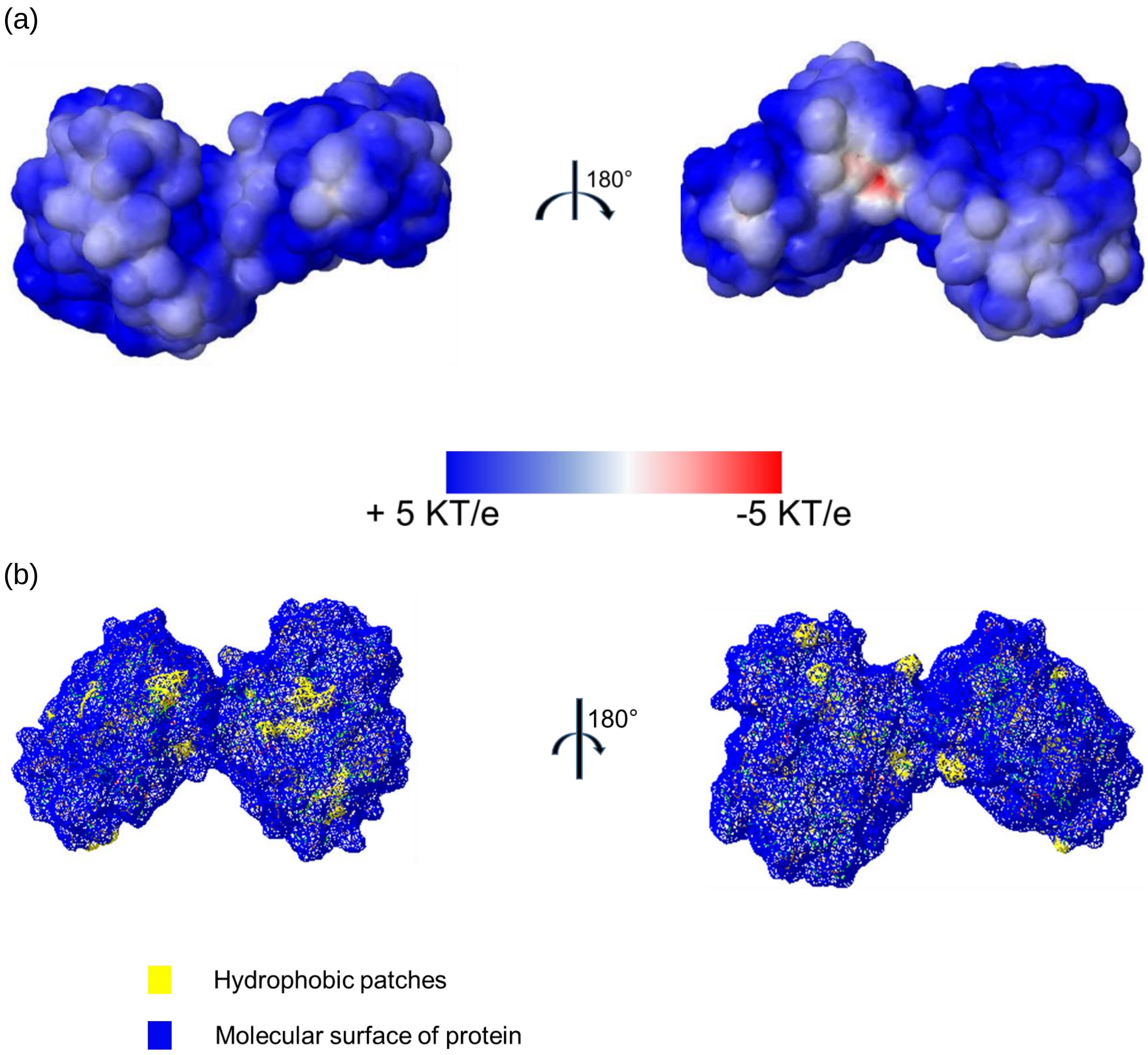

Supplementary Figure 20. **Electrostatic and Hydrophobic patch maps of Apomyoglobin (ApoMb: modified from PDB ID 3RJ6).** Maps show (a) the electrostatic surface potentials of ApoMb visualized as isocontours at +5.0 kT/e (blue) and -5.0 kT/e (red) using APBS and (b) predicted hydrophobic patches (yellow) visualized against the molecular surface (blue) using SPDB software.

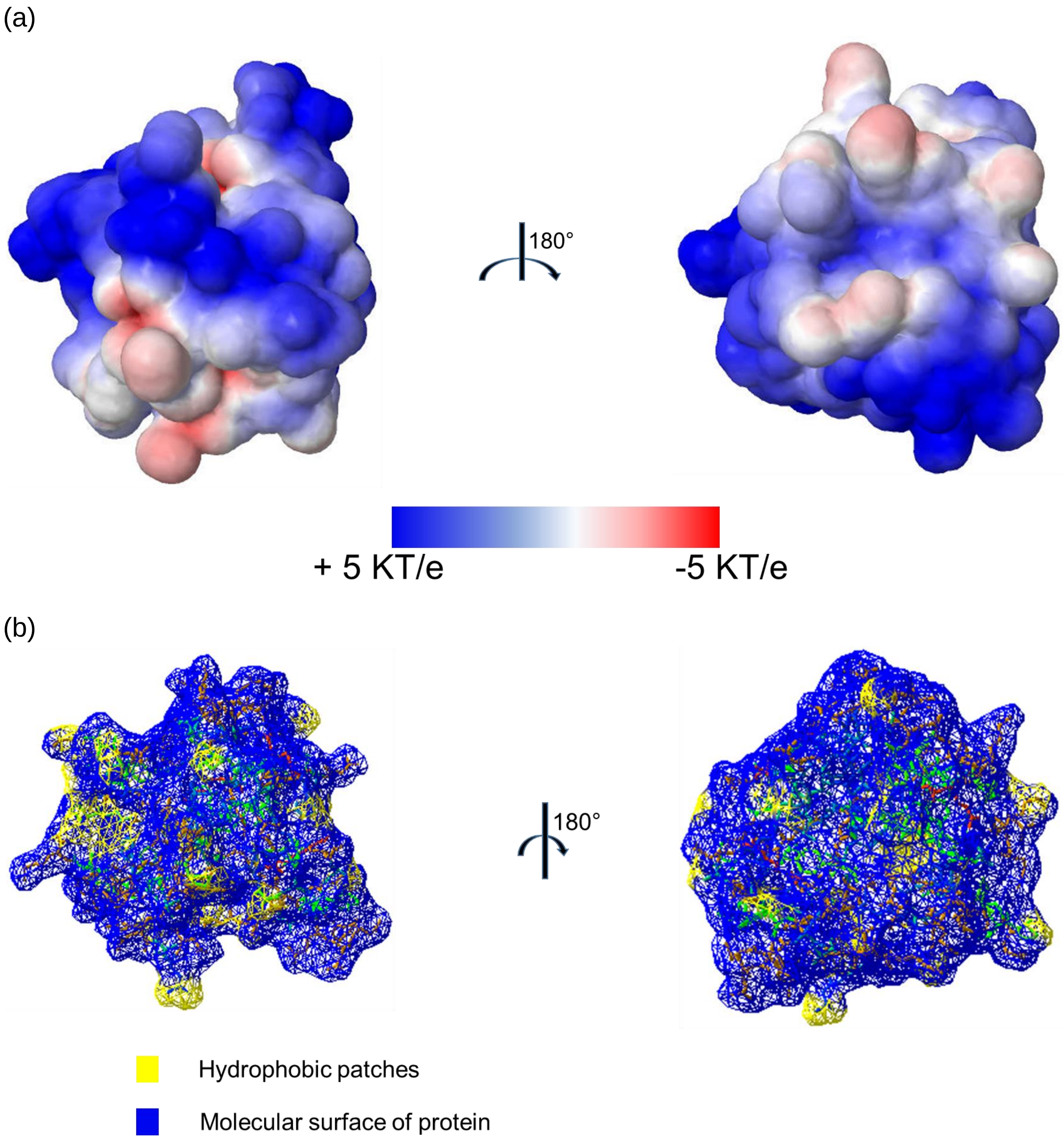

Supplementary Figure 21. **Electrostatic and Hydrophobic patch maps of beta lactoglobulin (β-lg: PDB ID 2Q2M).** Maps show (a) the electrostatic surface potentials of β-lg visualized as isocontours at +5.0 kT/e (blue) and -5.0 kT/e (red) using APBS and (b) predicted hydrophobic patches (yellow) visualized against the molecular surface (blue) using SPDB software.

(a)

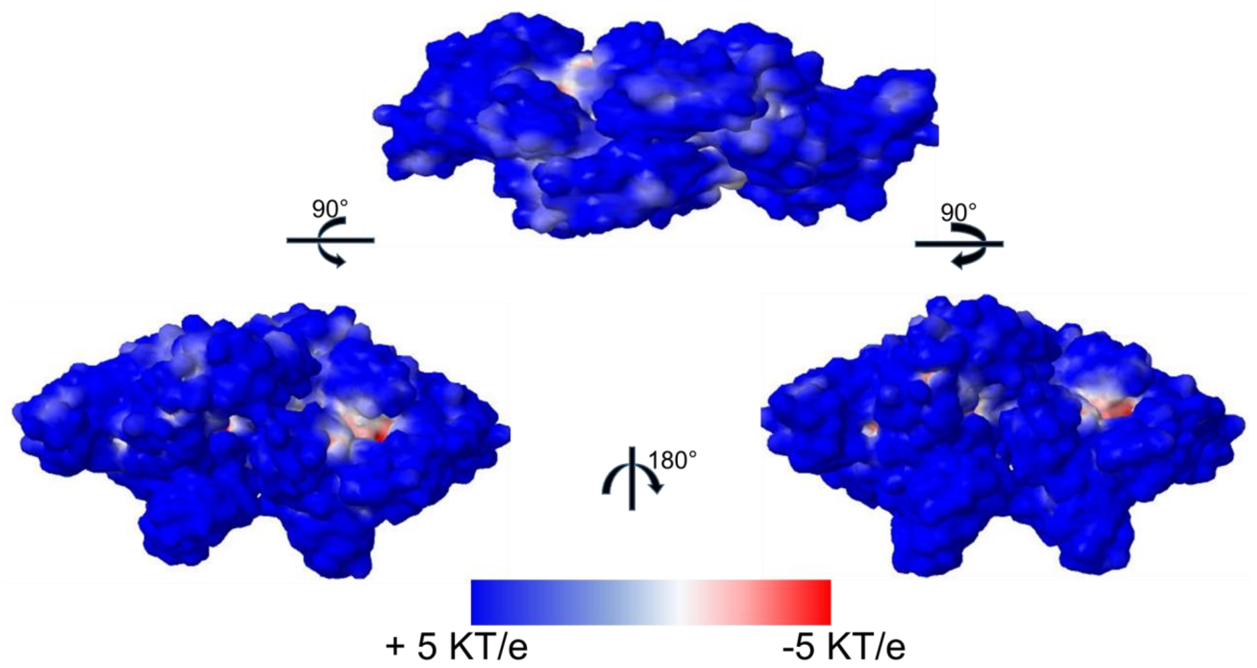

(b)

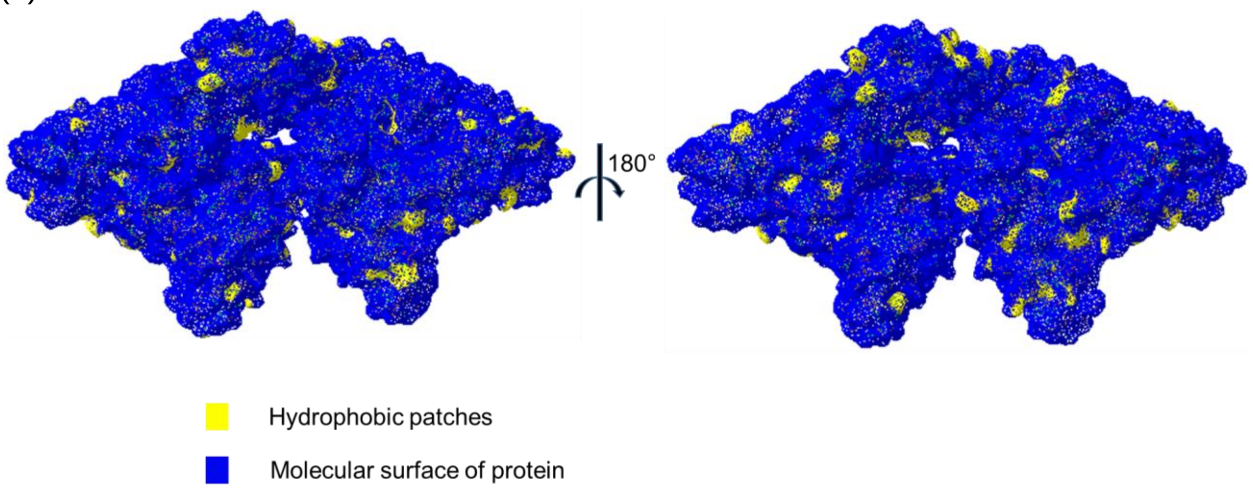

Supplementary Figure 22. **Electrostatic and Hydrophobic patch maps of bovine serum albumin (BSA: PDB ID 3V03).** Maps show (a) the electrostatic surface potentials of BSA visualized as isocontours at +5.0 kT/e (blue) and -5.0 kT/e (red) using APBS and (b) predicted hydrophobic patches (yellow) visualized against the molecular surface (blue) using SPDB software.

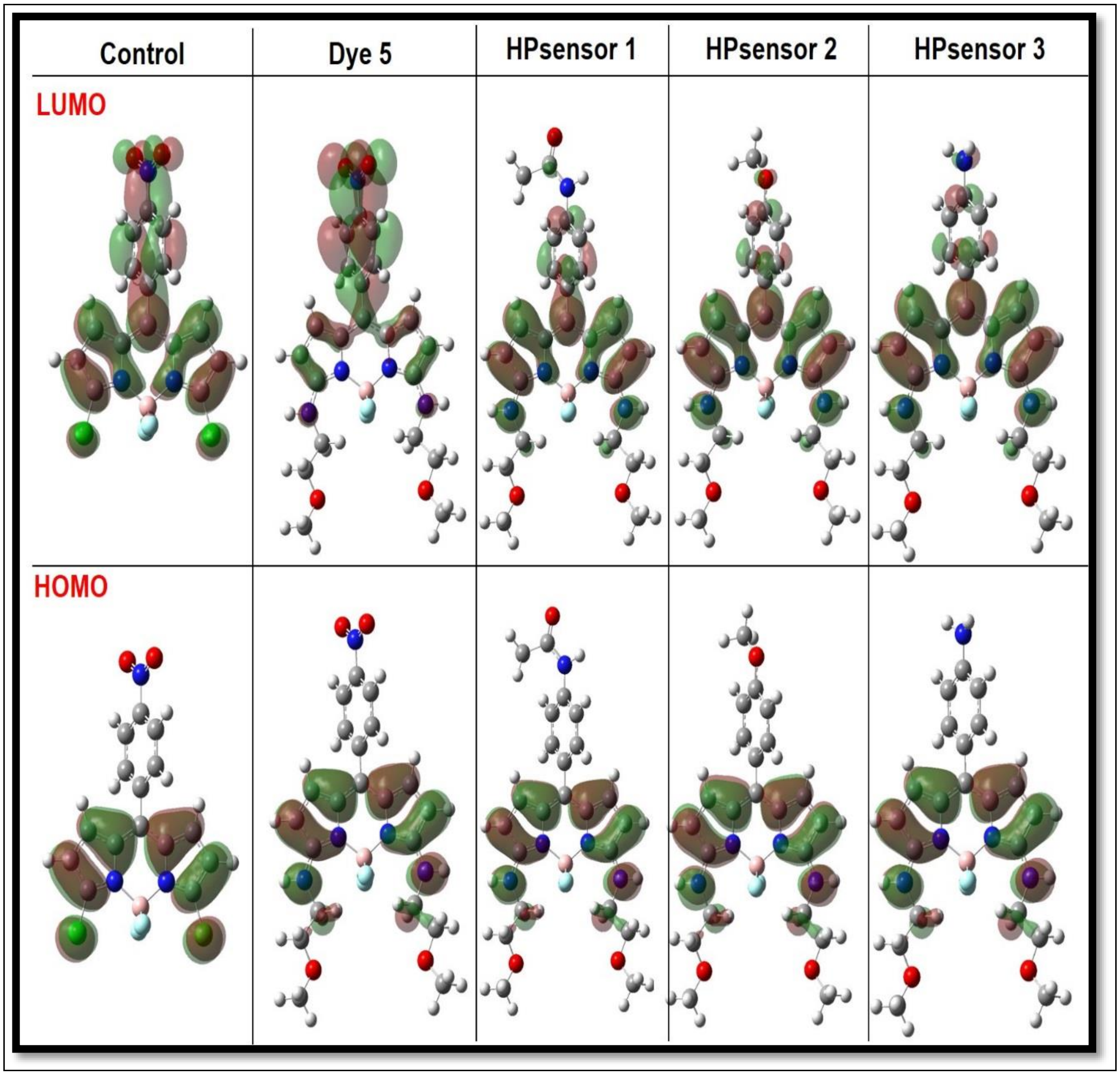

Supplementary Figure 23. Calculated frontier molecular orbitals for dyes in ethanol. (Top panel) LUMO energy distribution for control, dye 5, and HPsensors **1**, **2**, and **3**. (Bottom panel) HOMO energy distribution for control, dye **5** and HPsensors **1**, **2**, and **3**.

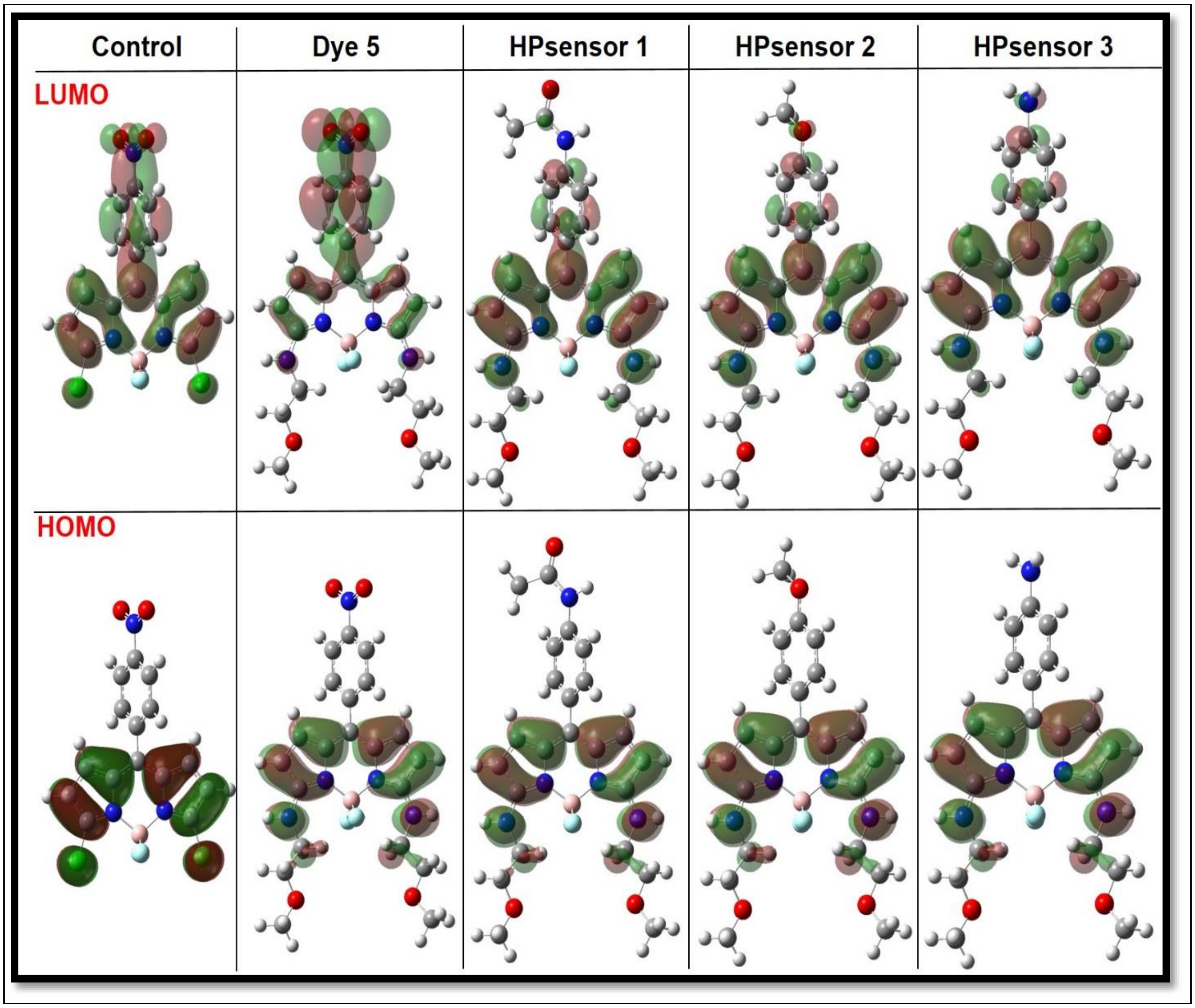

Supplementary Figure 24. Calculated frontier molecular orbitals for dyes in water. (Top panel) LUMO energy distribution for control, dye **5**, and HPsensors **1**, **2**, and **3**. (Bottom panel) HOMO energy distribution for control, dye **5** and HPsensors **1**, **2**, and **3**.

Supplementary Table 2. HOMO-LUMO energy gap calculation and associated wavelength of dyes (control, dye **5**, HPsensors **1**, **2**, and **3**) in vacuum, ethanol and water with range separated functional (HSEH1PBE) and 6-311g** basis set.

Vacuum					
**Molecule**	**energy**	**H0 (a.u.)**	**L0 (a.u.)**	**Gap (eV)**	**Wavelength (nm)**
Control	-2035.022	-0.23003	-0.14207	2.393532336	518.76
Dye **5**	-1612.674	-0.16895	-0.1044	1.75650878	706.06
HPsensor **1**	-1616.161	-0.16294	-0.08283	2.179921276	569
HPsensor **2**	-1522.741	-0.1572	-0.07547	2.224004068	557.48
HPsensor **3**	-1463.623	-0.15493	-0.07279	2.235160824	554.74
**Ethanol**					
**Molecule**	**energy**	**H0 (a.u.)**	**L0 (a.u.)**	**Gap (eV)**	**Wavelength (nm)**
Control	-2035.038	-0.22556	-0.1357	2.445234376	507.09
Dye **5**	-1612.696	-0.17143	-0.1112	1.638954668	756.92
HPsensor **1**	-1616.187	-0.16947	-0.08897	2.1905338	566.14
HPsensor **2**	-1522.762	-0.16877	-0.08705	2.223731952	557.56
HPsensor **3**	-1463.646	-0.16801	-0.08563	2.241691608	553.25
**Water**					
**Molecule**	**energy**	**H0 (a.u.)**	**L0 (a.u.)**	**Gap (eV)**	**Wavelength (nm)**
Control	-2035.039	-0.22707	-0.13734	2.441696868	508.13
Dye **5**	-1612.698	-0.17184	-0.11255	1.613375764	770.9
HPsensor **1**	-1616.189	-0.17022	-0.09014	2.179104928	569
HPsensor **2**	-1522.764	-0.16979	-0.08846	2.213119428	560.25
HPsensor **3**	-1463.648	-0.16932	-0.08731	2.231623316	555.98

Supplementary Table 3. HOMO-LUMO energy gap calculation and associated wavelength of dyes (control, dye **5**, HPsensors **1**, **2**, and **3**) in ethanol and with range separated functional (HSEH1PBE) and 6-311g** basis and internal rotation of up to 58°.

Rotation of HPsensor 2 in ethanol					
**Degree**	**energy**	**H0 (a.u.)**	**L0 (a.u.)**	**Gap (eV)**	**Wavelength (nm)**
5	-1522.686	-0.1709	-0.09822	1.977739088	626.91
15	-1522.716	-0.17039	-0.09635	2.014746864	615.61
25	-1522.741	-0.1697	-0.09334	2.077877776	596.71
35	-1522.755	-0.16915	-0.09043	2.142097152	578.82
45	-1522.761	-0.16885	-0.08835	2.1905338	566.14
58	-1522.762	-0.16877	-0.08705	2.223731952	557.56
**Rotation of HPsensor 3 in ethanol**					
**Degree**	**energy**	**H0 (a.u.)**	**L0 (a.u.)**	**Gap (eV)**	**Wavelength (nm)**
5	-1463.574	-0.16971	-0.09547	2.020189184	613.78
15	-1463.603	-0.16926	-0.09365	2.057469076	602.74
25	-1463.627	-0.16864	-0.0908	2.118150944	585.38
35	-1463.64	-0.16819	-0.08816	2.177744348	569.34
45	-1463.645	-0.16799	-0.08643	2.219378096	558.74
56	-1463.646	-0.16801	-0.08563	2.241691608	553.25

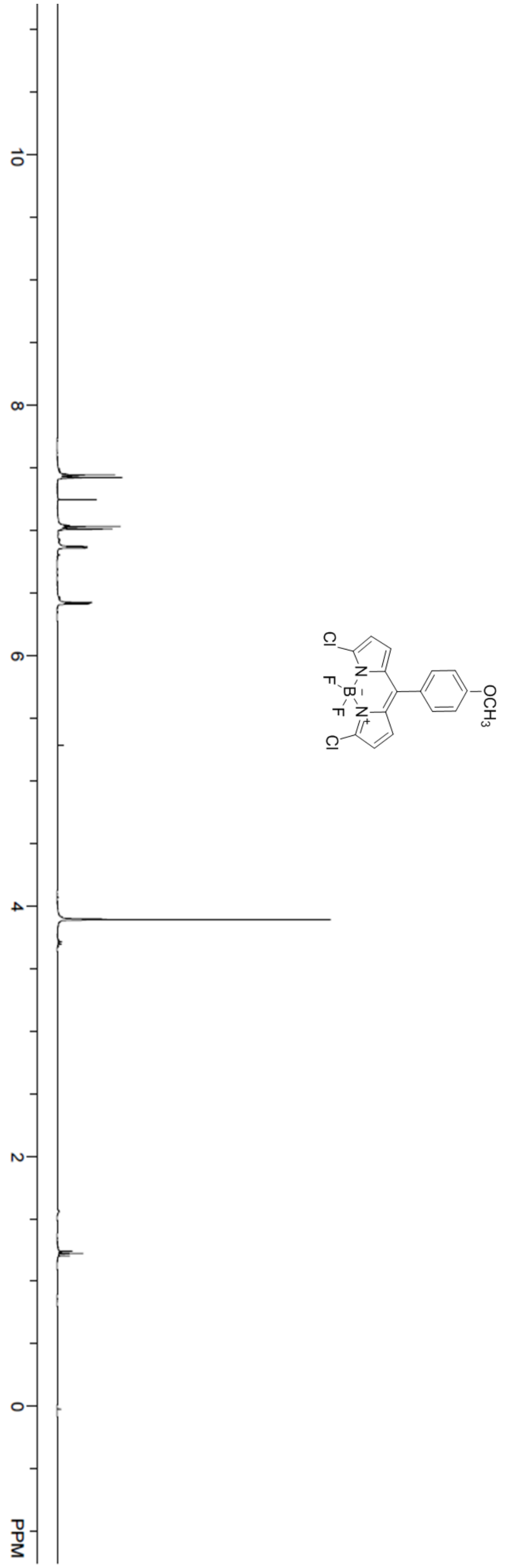

Supplementary Figure 25a ^{1}H NMR spectrum of control dye in $CDCl_3$ solution.

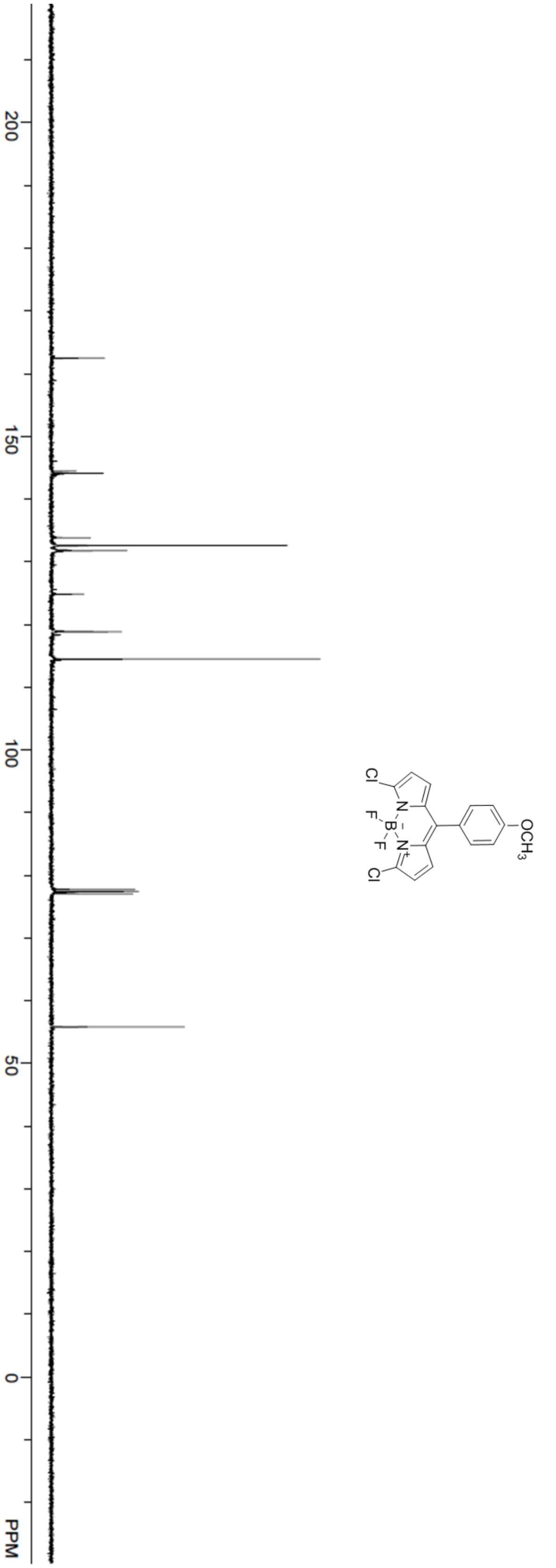

Supplementary Figure 25b ^{13}C NMR spectrum of control dye in $CDCl_3$ solution.

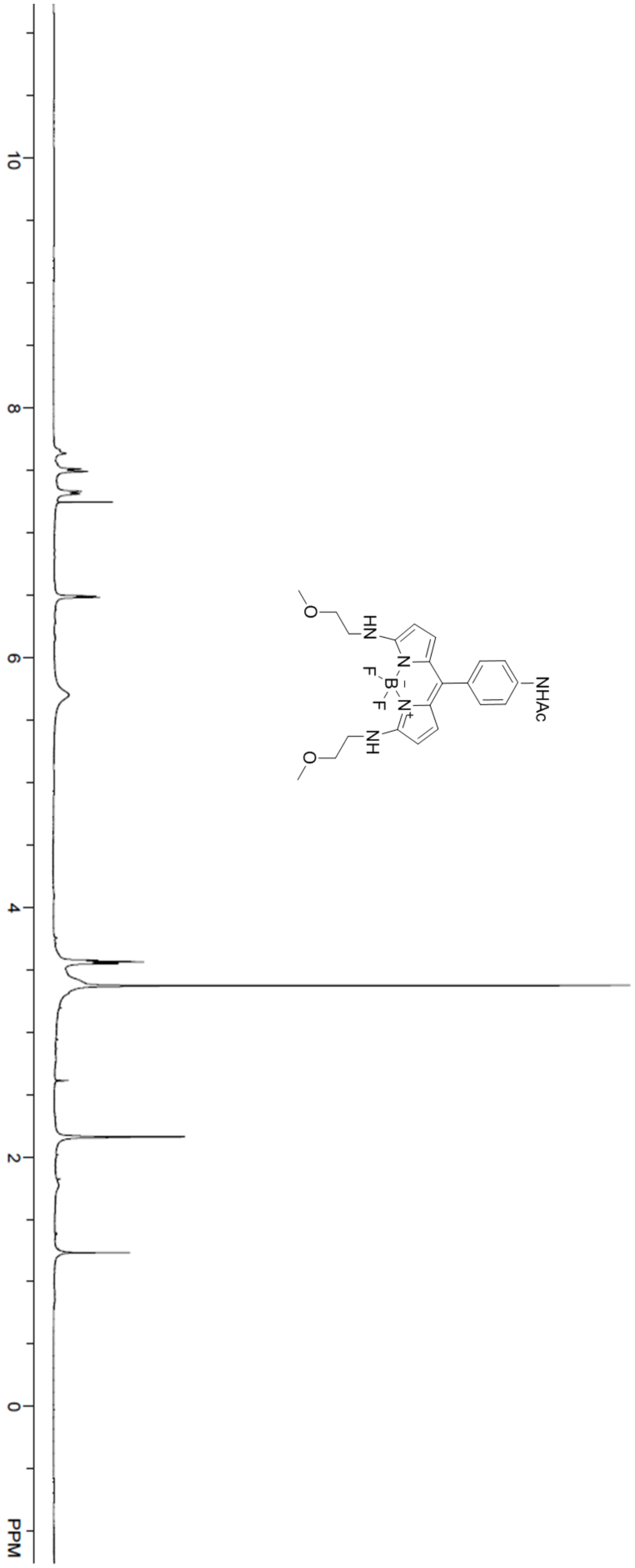

Supplementary Figure 26a ^{1}H NMR spectrum of HPsensor **1** in $CDCl_3$ solution.

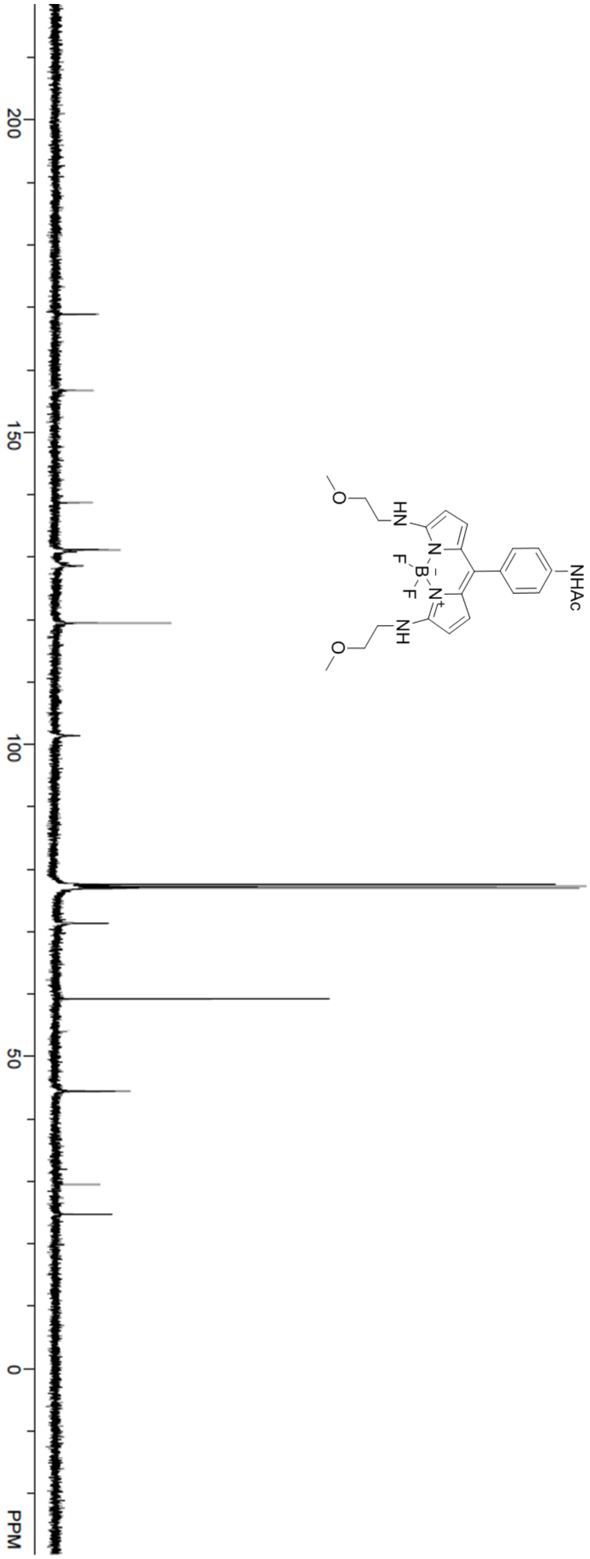

Supplementary Figure 26b ^{13}C NMR spectrum of HPsensor **1** in $CDCl_3$ solution.

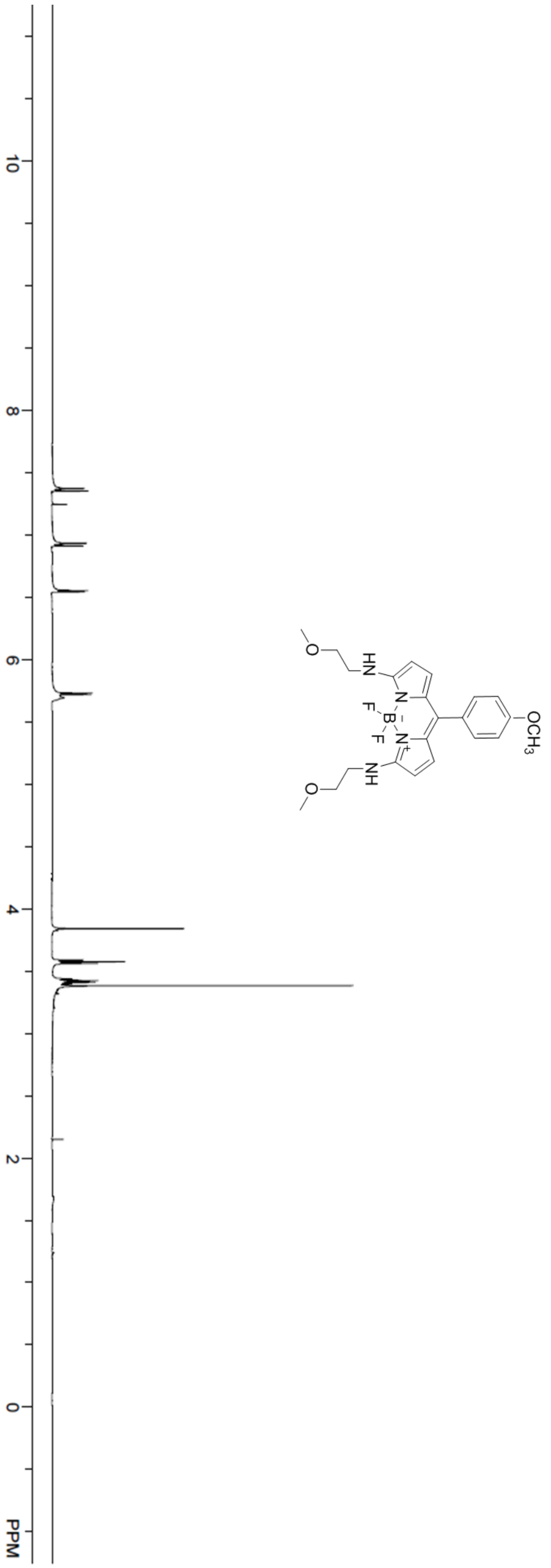

Supplementary Figure 27a ^{1}H NMR spectrum of HPsensor **2** in $CDCl_3$ solution.

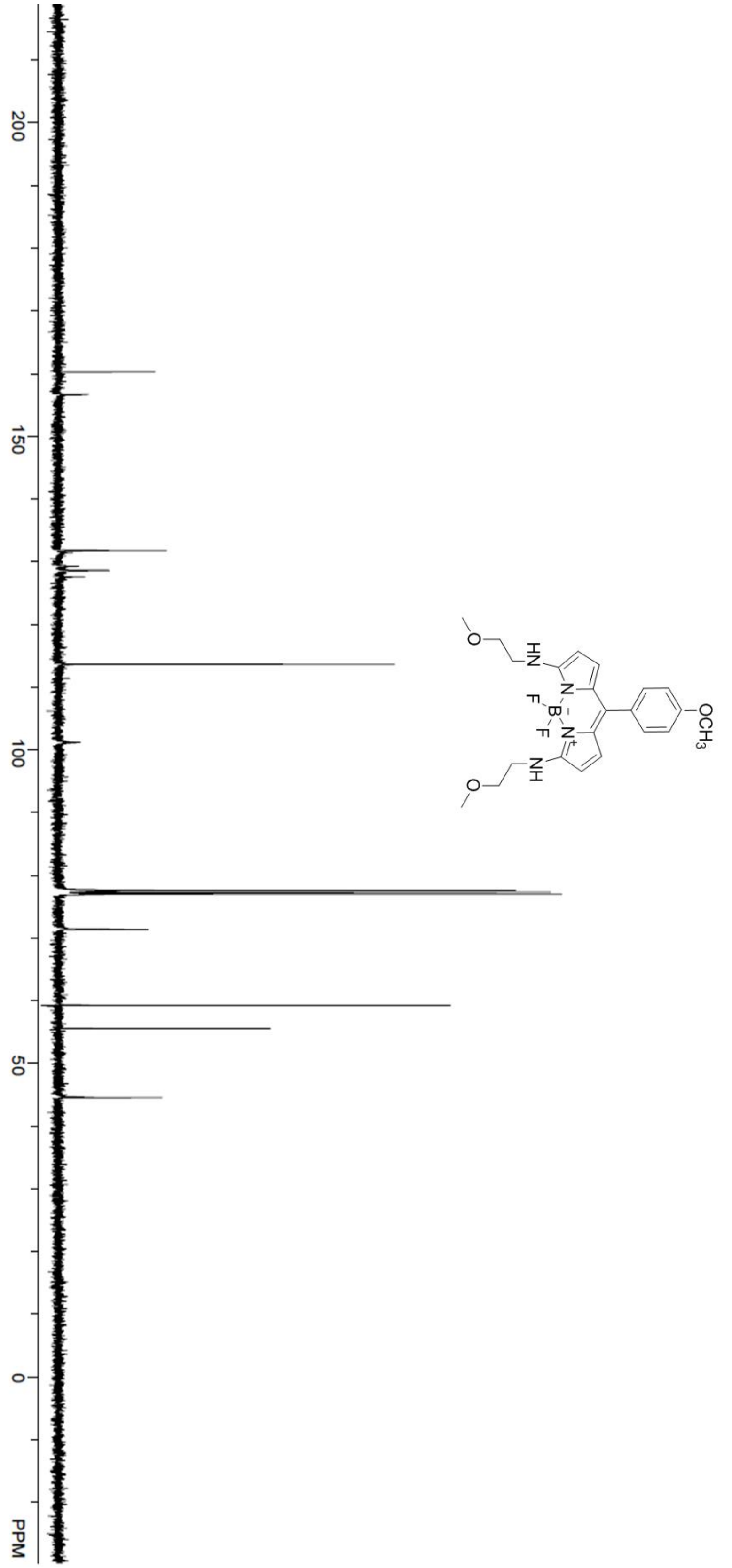

Supplementary Figure 27b ^{13}C NMR spectrum of HPsensor **2** in $CDCl_3$ solution.

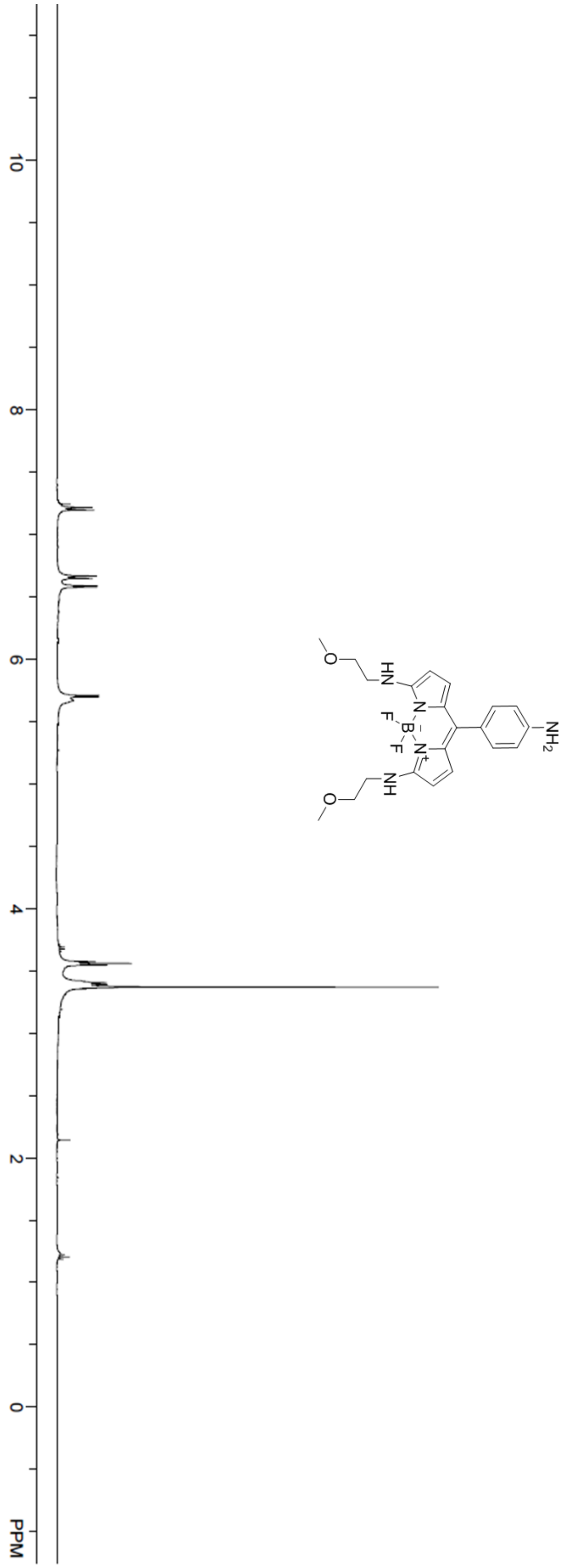

Supplementary Figure 28a ^{1}H NMR spectrum of HPsensor **3** in $CDCl_3$ solution.

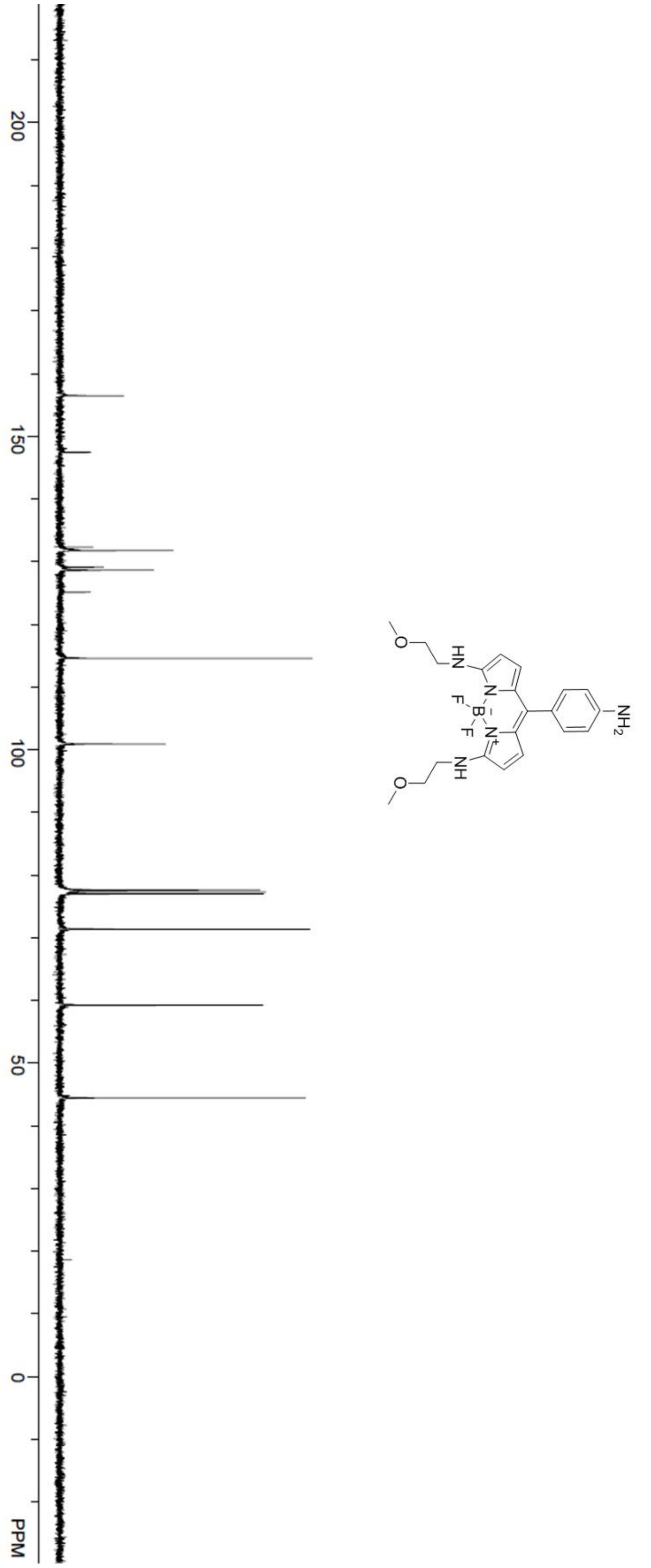

Supplementary Figure 28b ^{13}C NMR spectrum of HPsensor **3** in $CDCl_3$ solution.

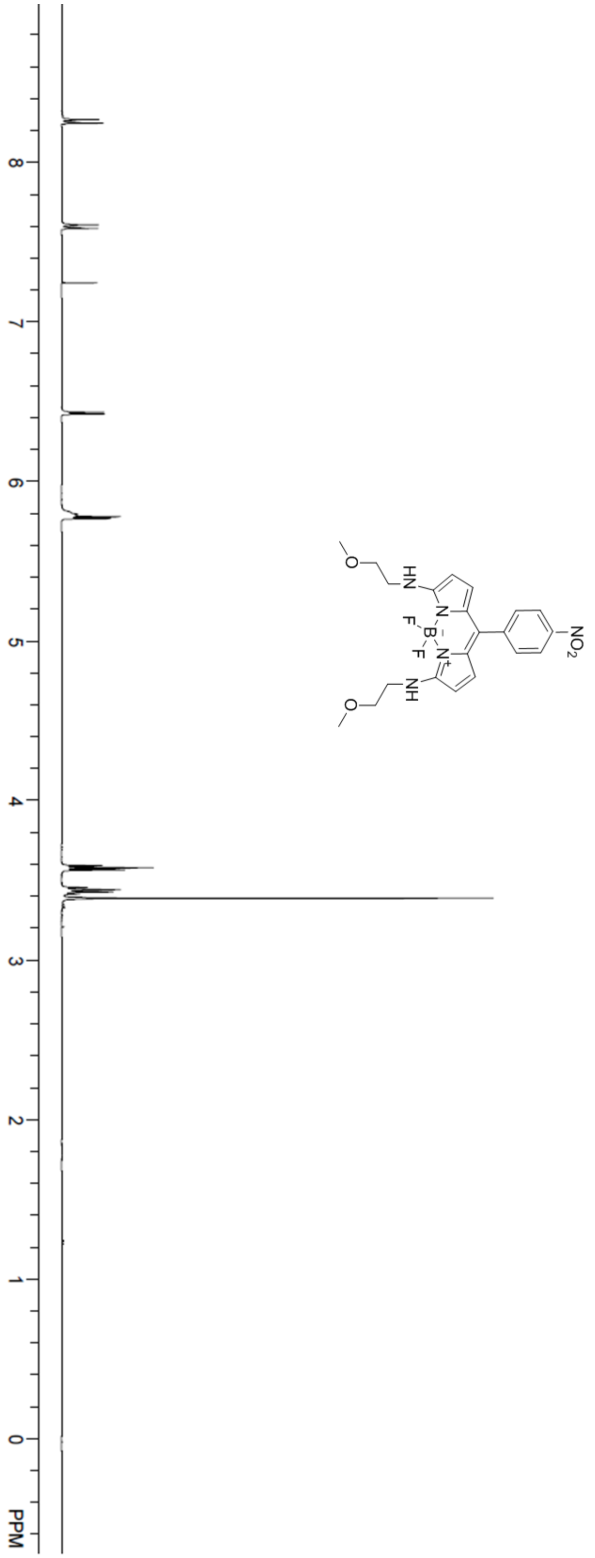

Supplementary Figure 29a ^1H NMR spectrum of dye **5** in $CDCl_3$ solution.

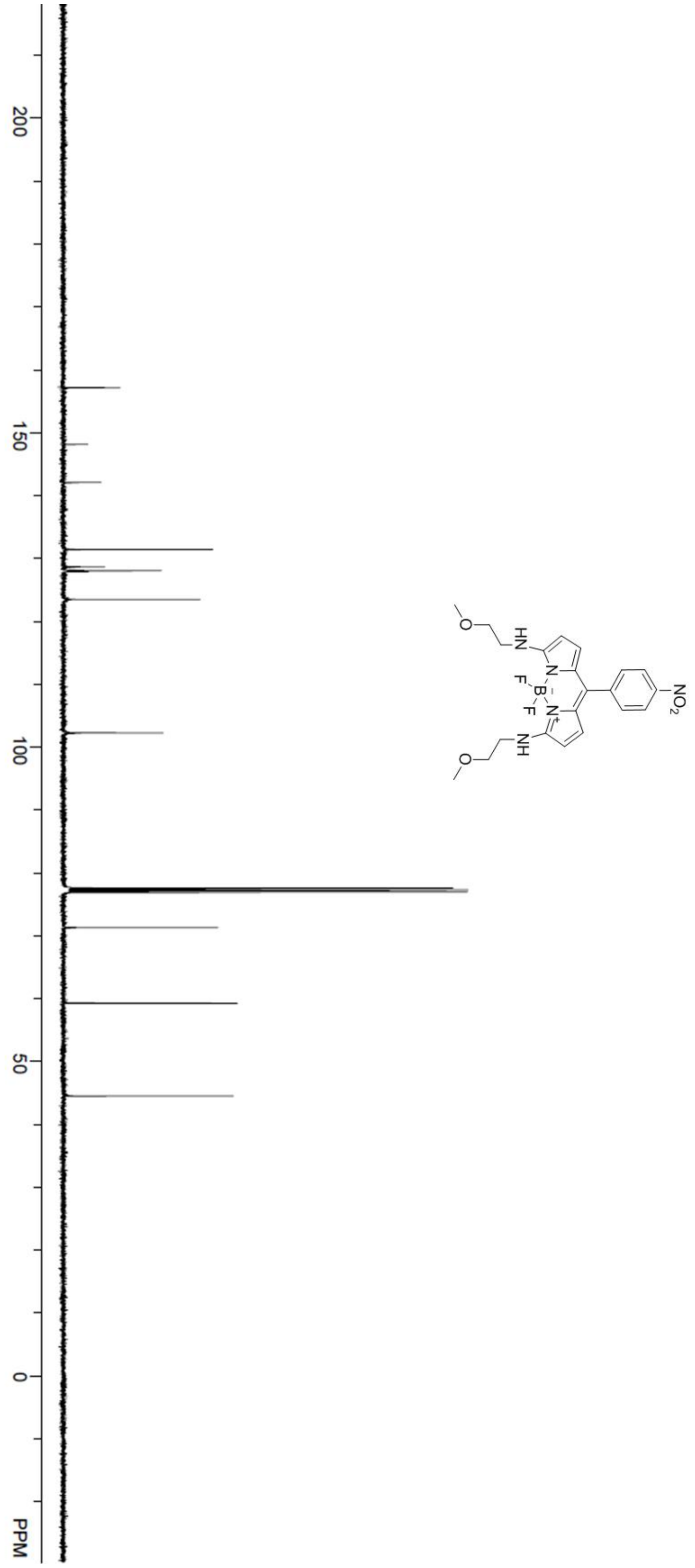

Supplementary Figure 29b ^{13}C NMR spectrum of dye **5** in $CDCl_3$ solution.

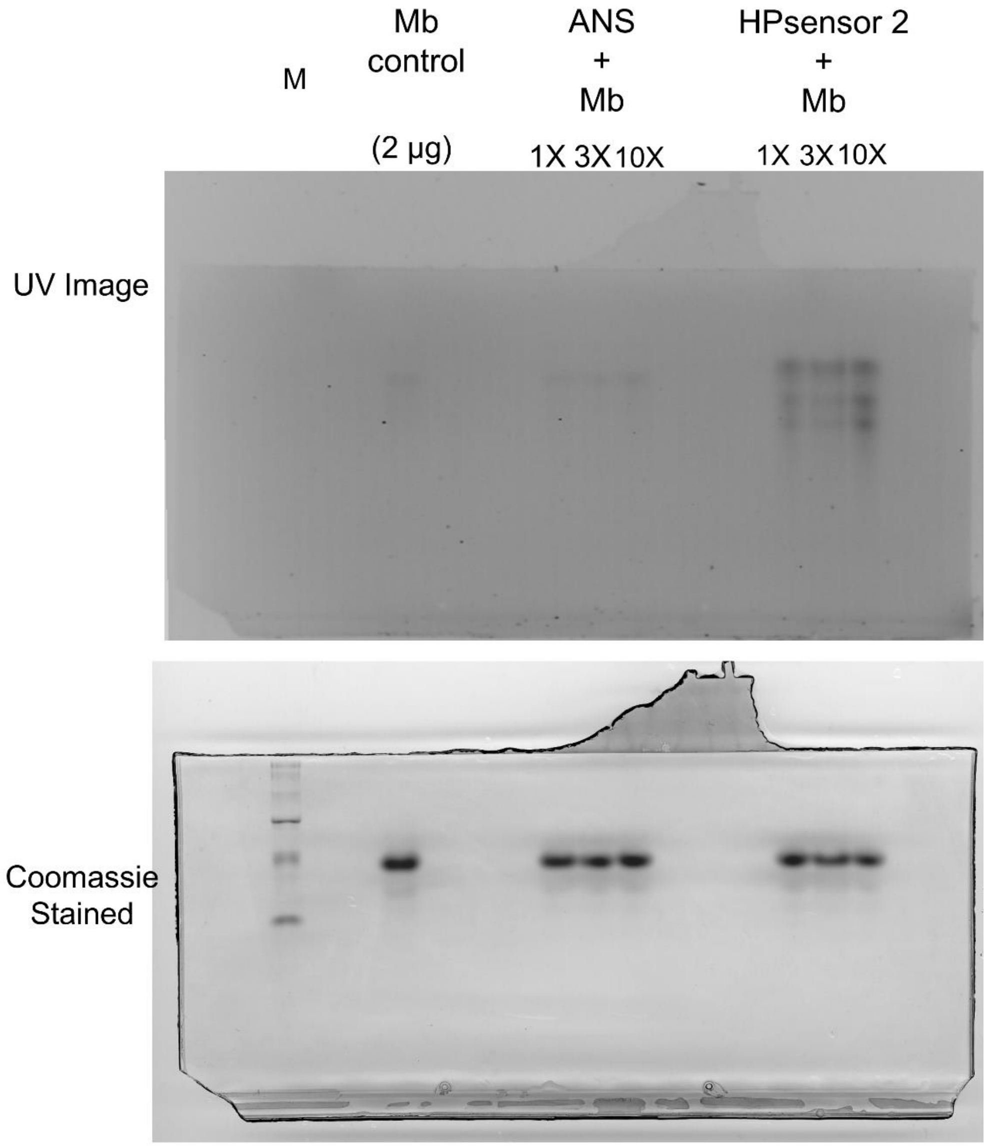

Supplementary Figure 30. Full length gel of Native PAGE of 2 µg Mb with 1X, 3X, and 10X Dye (ANS or HPsensor 2). Full gel image of 2 µg of Mb incubated with 1X, 3X, and 10X concentration of dyes (ANS or HPsensor 2) for 1 h at 25 °C. The Mb and protein was run on a 15% gel for 6 h at 80 V. M – indicates molecular weight marker.

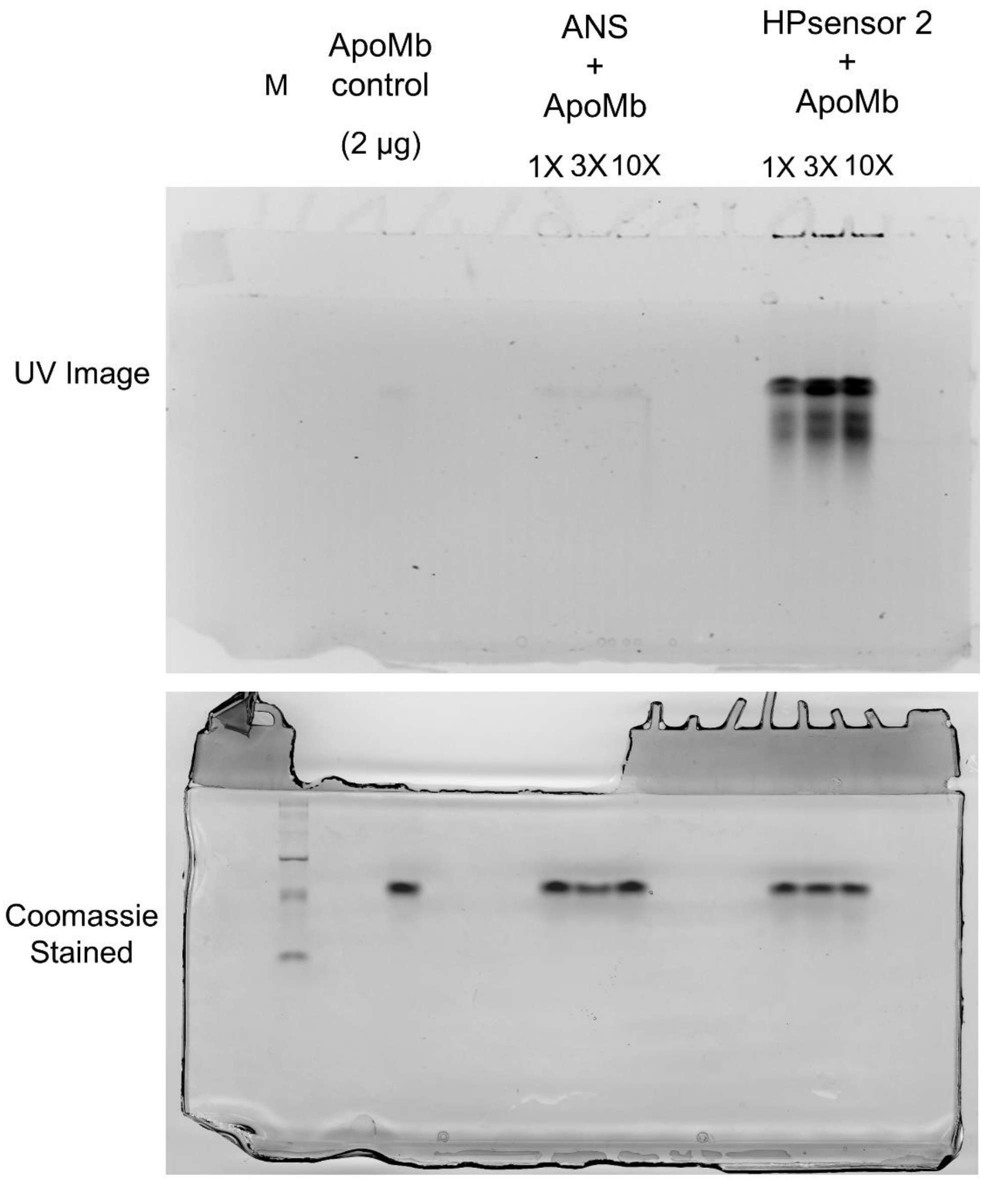

Supplementary Figure 31. Full length gel of Native PAGE of 2 µg ApoMb with 1X, 3X, and 10X Dye (ANS or HPsensor 2). Full gel image of 2 µg of ApoMb incubated with 1X, 3X, and 10X concentration of dyes (ANS or HPsensor 2) for 1 h at 25 °C. The ApoMb protein was run on a 15% gel for 6 h at 80 V. M – indicates molecular weight marker.

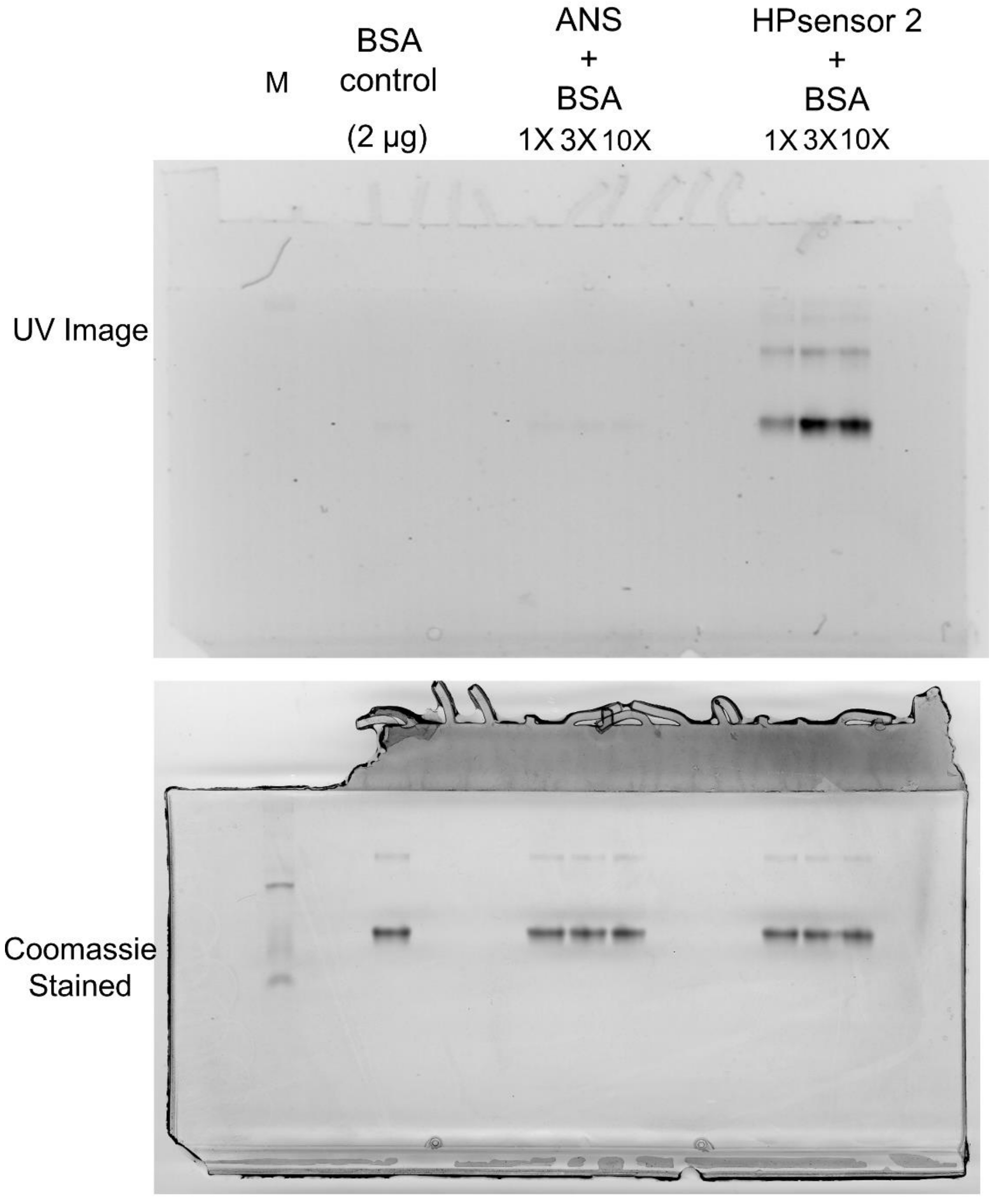

Supplementary Figure 32. Full length gel of Native PAGE of 2 µg BSA with 1X, 3X, and 10X Dye (ANS or HPsensor 2). Full gel image of 2 µg of ApoMb incubated with 1X, 3X, and 10X concentration of dyes (ANS or HPsensor 2) for 1 h at 25 °C. The BSA protein was run on a 10% gel for 3 h at 80 V. M – indicates molecular weight marker.

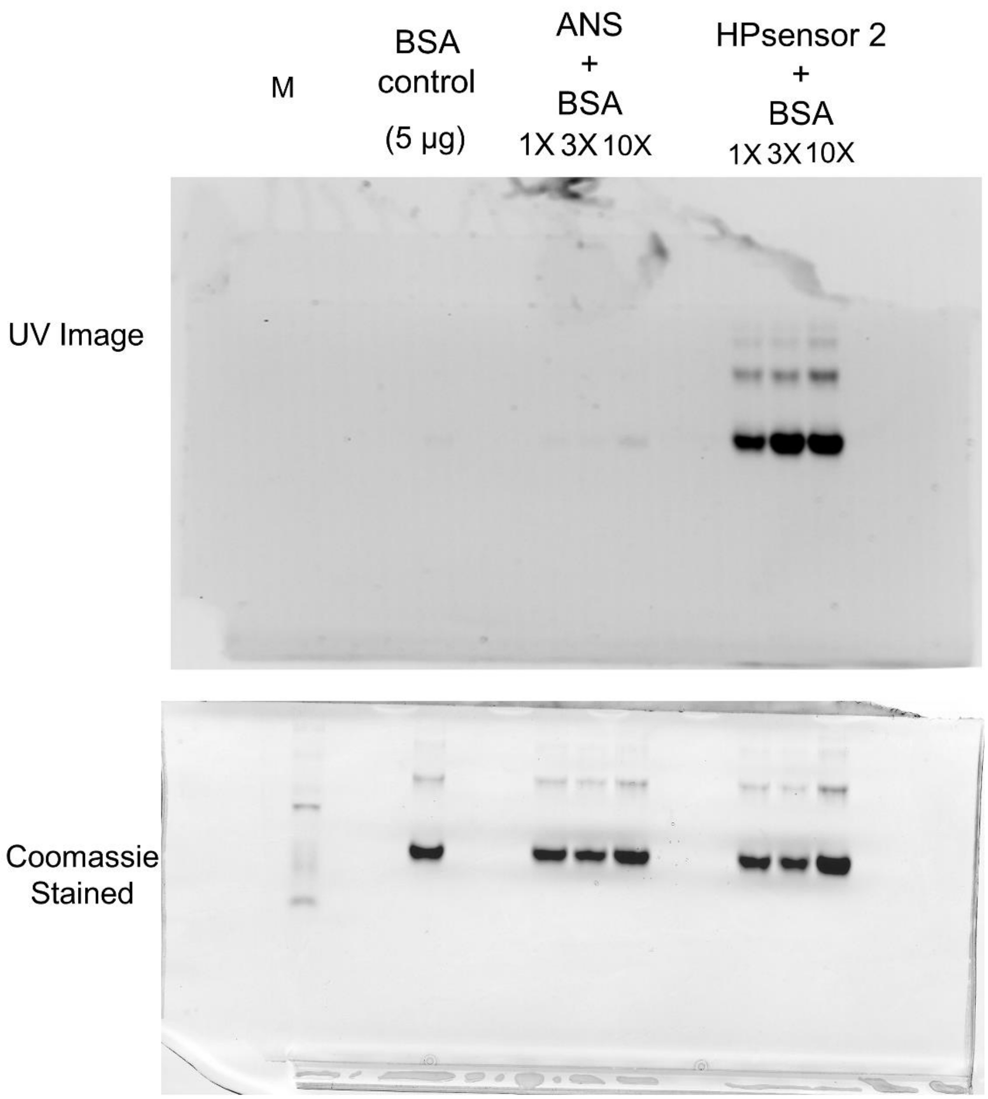

Supplementary Figure 33. **Full length gel of Native PAGE of BSA (5 µg) with ANS and HPsensor 2.** Full length gel of 5 µg of BSA incubated with dyes (ANS or HPsensor **2**) at 1X, 5X, and 25X concentration for 1 h at room temperature. BSA was then run on 10% Tris-HCl gel for 3 h at 80 V before exposure to UV light or Coomassie blue. M – indicates molecular weight marker.

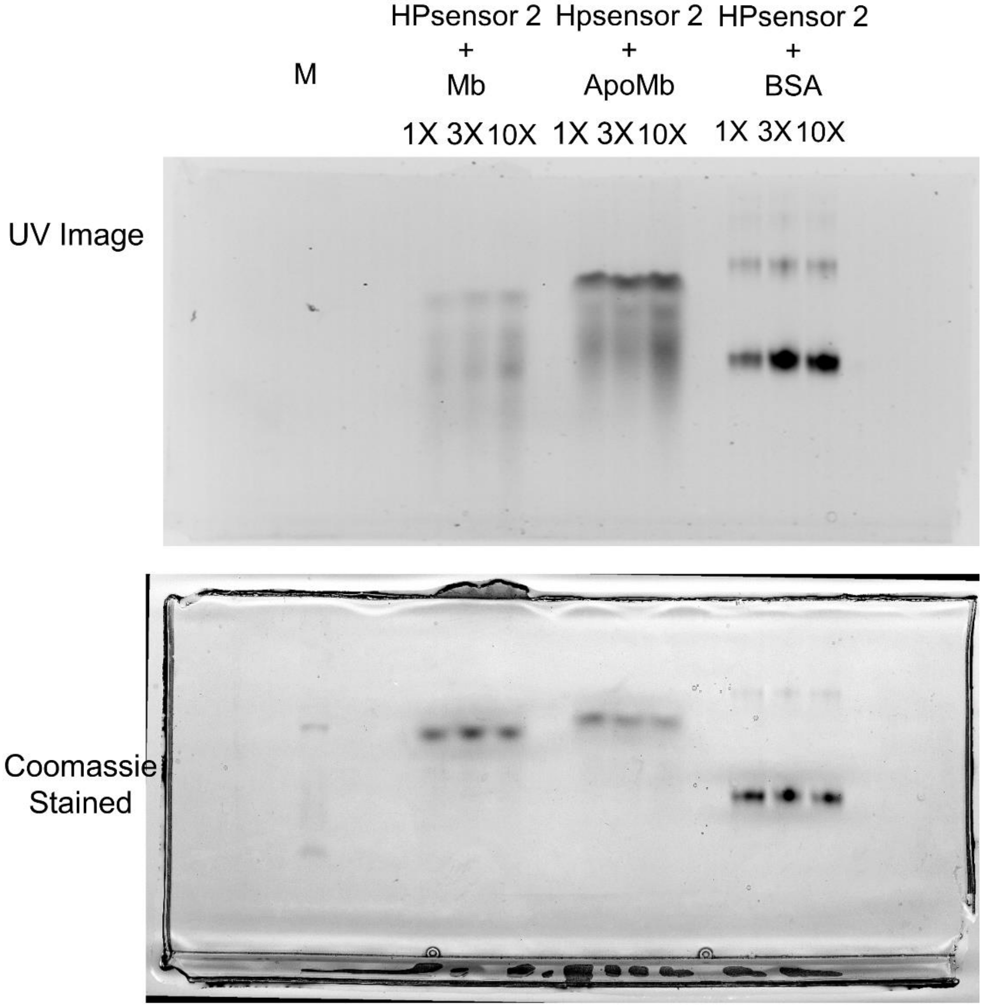

Supplementary Figure 34. **Full length gel of Native PAGE of 2 µg of Proteins [Myoglobin (Mb), Apomyoglobin (ApoMb), BSA] with HPsensor 2.** Full length gel of 2 µg of each protein incubated with HPsensor **2** at 1X, 3X, and 10X concentration for 1 h at room temperature. Proteins were then run on 10% Tris-HCl gel for 4 h at 80 V before exposure to UV light or Coomassie blue. M – indicates molecular weight marker.

References

1 Fink, A. L., Oberg, K. A. & Seshadri, S. Discrete intermediates versus molten globule models for protein folding: characterization of partially folded intermediates of apomyoglobin. *Fold. Des.* **3**, 19-25, doi:10.1016/s1359-0278(98)00005-4 (1998).

2 Zhu, S. *et al.* Highly water-soluble neutral near-infrared emissive BODIPY polymeric dyes. *J. Mater. Chem.* **22**, 2781-2790, doi:Doi 10.1039/C2jm14920f (2012).

3 Carpentieri, U., Myers, J., Thorpe, L., III, C. W. D. & Haggard, M. E. Copper, Zinc, and Iron in Normal and Leukemic Lymphocytes from Children. *Cancer Res.* **46**, 981 -984 (1986).

4 Page, M. J. & Cera, E. D. Role of Na^+ and K^+ in Enzyme Function. *Physiol. Rev.* **86** 1049–1092, doi:10.1152/physrev.00008.2006.-Metal (2006).
